# Sol–Gel-Encapsulated Biofilms on Biochar Effectively Degrade PCBs and Extend Biodegradation Activity under Environmentally Relevant Conditions

**DOI:** 10.1021/acs.est.6c02869

**Published:** 2026-09-25

**Authors:** Qin Dong, Weilun Zhao, Andres Martinez, Rachel F. Marek, Keri C. Hornbuckle, Xueshu Li, Hans-Joachim Lehmler, Gregory H. LeFevre, Timothy E. Mattes

**Affiliations:** † Department of Civil and Environmental Engineering, 4083 University of Iowa, 4105 Seamans Center, Iowa City, Iowa 52242, United States; ‡ IIHR–Hydroscience and Engineering, University of Iowa, 100 C. Maxwell Stanley Hydraulics Laboratory, Iowa City, Iowa 52242, United States; § Department of Occupational and Environmental Health, University of Iowa, Iowa City, Iowa 52242, United States

**Keywords:** sol−gel, encapsulation, PCB biodegradation, environmental condition impacts

## Abstract

Attaching aerobic, PCB-degrading cells to carbon-based sorbent materials prior to sediment application is a feasible approach for bioaugmenting contaminated sediments to improve remediation strategies. Furthermore, encapsulation of attached cells with a novel, tailored sol–gel material can protect biofilms from unfavorable environmental conditions that interfere with biofilm stability and create stress that threatens long-term biofilm viability. We systematically evaluated biodegradation and sorption of three PCB congeners (PCBs 4, 18, and 52), quantified biphenyl dioxygenase gene (*bphA*) abundance and expression, and characterized sol–gel and biofilm integrity of sol–gel-coated PCB-degrading biofilms on black carbon over 45 days under environmentally relevant conditions. Sol–gel coatings did not significantly inhibit PCB sorption to the black carbon, and sol–gel-coated biofilms achieved 72–92% PCB removal within 45 days, comparable to uncoated biofilms (>91%). Although PCB biodegradation rates in uncoated biofilms were >5-fold higher than sol–gel-coated biofilms, sol–gel-coated biofilms maintained >2-fold higher *bphA* expression levels than uncoated biofilms after 45 days of treatment. Lower temperature (10 °C) decreased PCB biodegradation extent regardless of sol–gel coating but significantly increased (10-fold higher) *bphA* expression levels in sol–gel-coated biofilms. Salinity (20 g/L) negatively impacted *bphA* expression levels (>13-fold lower after 45 days) for all treatments. Our novel sol–gel design demonstrates dual benefits: to sorb PCBs from the environment to biofilms and provide carbon sources for PCB cometabolism, thereby protecting and supporting biofilms for potential extended activity in future field applications.

## Graphical abstract

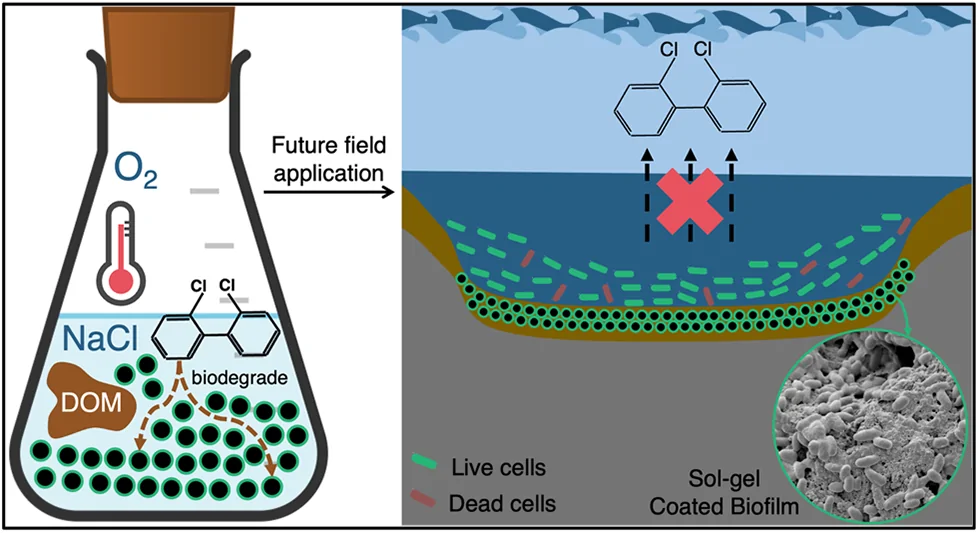

## Introduction

Lower-chlorinated polychlorinated biphenyls (LC-PCBs), defined as PCBs with four chlorines or fewer, comprise a significant fraction of commercial PCB mixtures (*e.g*., Aroclors) and can also be formed by slow anaerobic dechlorination of higher-chlorinated PCBs (HC-PCBs; Cl number >4).^1,2^ LC-PCBs more readily volatilize from sediment to air than HC-PCBs.^3–6^ Airborne LC-PCBs cause adverse health impacts (*e.g*., brain development, immune systems, and endocrine activity) for nearby communities and ecosystems,^7–10^ although sediment-bound PCB exposure may present higher cancer risk and persistence.^11^

Bioremediation is cost-effective and sustainable compared with more conventional approaches for managing PCBs in sediment (*e.g*., dredging,geosorbents).^12–14^ Bioaugmentation is an emerging bioremediation approach that introduces high cell abundance at low cost to accelerate PCB biodegradation in sediments. Bioaugmenting suspended aerobic PCB-degrading cells (*e.g*., *Paraburkholderia xenovorans* strain LB400) in sediment can oxidize up to hexachlorinated biphenyls and potentially cleave aromatic rings to yield chlorobenzoates as expected end products (depending on PCB structure and Cl position), concomitantly mitigating PCB flux to air.^15–17^ Although little is known about the rates of individual PCB congener biotransformation by suspended LB400 cells and other aerobic PCB-degrading bacteria, they are generally more rapid than anaerobic PCB dechlorination rates.^18,19^ PCB biotransformation by LB400 is mainly cometabolic through expression of biphenyl catabolic pathway (*bph*) enzymes, which are induced by biphenyl and other compounds.^20^ No internal energy storage molecules (*i.e*., polyhydroxyalkanoates) are known to drive PCB biotransformation in LB400; energy comes from oxidation of *bph* pathway inducers.^20^

Suspended cells have demonstrated transient biodegradation efficiency and short survival times in sediments due to fluctuating environmental conditions (*e.g*., transport, toxicity, nutrient availability).^21–25^ Therefore, sediment bioaugmentation approaches to deliver cells for *in situ* applications are necessary. Carbon-based sorbents with both high sorption and electron storage capacity^26–29^ have been developed for cell immobilization and tested as sediment amendments in the field.^24,30–35^ Our recent study demonstrates that corn kernel biochar can induce aerobic PCB-degrading biofilm formation on surfaces and enhance PCB biodegradation gene expression (*e.g*., *bph* genes) when growing LB400 with the biochar for 10 days with the addition of carbon substrates in liquid solution.^36^ These studies suggest that bioaugmenting biofilms on black carbon (BC) surfaces mitigates biomass loss compared to suspended cells.^22,23^

Although biofilm formation on BC surfaces induces PCB biodegradation activity, biofilm abundance and activity decrease over time following *in situ* deployment,^37–39^ suggesting that methods to further protect biofilms from adverse environmental conditions are also needed. Encapsulation has been increasingly applied to cell storage, which is reported to even increase microbial activity.^40–42^ To enhance/secure biofilm stability, we recently developed a novel method to encapsulate biofilms with sol–gel aimed at protecting biofilms against adverse environmental changes (*e.g*., high salinity and shear force). The sol–gel encapsulation process involves hydrolyzing, condensing, and aging the silica gel precursor to form a porous and physically stable sol–gel that completely covers biofilm-biochar surfaces. Sol–gel encapsulation maintains biofilm integrity and cell viability for extended time periods (over three months) compared with unencapsulated biofilms on BC surfaces (25 days).^43^ Nevertheless, the impacts of sol–gel encapsulation on PCB-degrading biofilms are poorly understood; the effect of unfavorable environmental conditions on biodegradation performance needs further investigation.

The objective of this study was to investigate the performance of sol–gel-encapsulated LB400 biofilm-enriched biochar under various environmental conditions with the long-term goal of improving *in situ* sediment applications and mitigating air flux from sediment to air. We hypothesize that the sol–gel structure allows mass transfer of hydrophobic organic compounds (*i.e*., PCBs) from the bulk liquid to the biochar surface for subsequent biodegradation by the biofilms and protects biofilm viability/activity against adverse environmental conditions. We evaluated sorption potentials of sol–gel and biochar and impacts on PCB mass transfer. Additionally, we assessed LC-PCB biodegradation performance (PCB mass changes, biphenyl dioxygenase biomarker gene abundance/expression) of encapsulated biofilm-enriched biochar under environmentally relevant conditions (emulating the conditions of a real PCB-contaminated site^44–46^ in a batch-scale test) and compared biofilm integrity and viability before/after treatment. We demonstrated that this novel encapsulation approach proved useful for protecting biofilm integrity and inducing biofilm activity over extended time periods (45-day experiment) in addition to maintaining PCB biodegradation performance.

## Methods and Materials

### Chemicals and Materials

Three LC-PCBs (defined as PCBs with ≤4 chlorines) were chosen to test biofilm degradation performance. Individual congeners PCB 4 (2,2′-dichlorobiphenyl, Lot#001) was purchased from AccuStandard, Inc. (New Haven, CT, USA), and PCB 18 (2,2′,5-trichlorobiphenyl, Lot#MP143P71) was purchased from Milestone PharmaTech USA Inc. (Metuchen, NJ, USA). Both PCB 4 and PCB 18 were authenticated by the Iowa Superfund Research Program Synthesis Core.^47–49^ PCB 52 (2,2′,5,5′-tetrachlorobiphenyl) was synthesized and authenticated as reported previously.^50^ PCB 4, PCB 18, and PCB 52 were selected for biodegradation tests because these PCBs have two, three, and four chlorines, respectively, at similar positions. LC-PCBs are more volatile than higher-chlorinated PCBs (Cl number >4), thus posing greater airborne exposure risks to the public.^5,51^ PCB 52 inhalation is reported to cause behavioral impacts on rats.^52^ Preparation of PCB master mixes in solvents can be found in S1.1. Corn kernel biochar (CKB) was selected as the biofilm support because it improved LB400 cell attachment and functional gene expression. CKB characteristics can be found in prior work.^36^ CKB was obtained from Dr. Albert Ratner’s research group.^53^ Solid-phase microextraction (SPME) fibers were acquired from Fiberguide Industries, Inc. (Stirling, NJ). Humic acid (Sigma-Aldrich) was chosen with a molecular size (21Å)^46^ within the range of sol–gel pore sizes (16–46Å), with the goal of emulating a simplified condition of natural organic matter interfering with PCB diffusion through pores.

### Experimental Design

#### Aqueous PCB Quantification by SPME Fiber Passive Samplers

SPME-water partition coefficients were determined under different environmental conditions, where method details and results are found in S1.2.1-S1.2.3 and Figures S1–S2. SPME fiber application to quantify freely dissolved PCB concentrations in the presence of sorbents (*e.g*., CKB) is described in S1.2.4 and Figure S3.

#### PCB Sorption on Plain CKB, Plain Sol–Gel, Uncoated Killed Biofilms-CKB, and Sol–Gel-Coated Killed Biofilms-CKB

Partition coefficients between sorbents and water for PCBs (*K*_d_, L/kg) were determined to indicate the sorption potential of PCB congeners on target sorbents and are described in S1.3 and Figure S4. Freely dissolved PCB concentrations in the presence of target sorbents (plain CKB, plain sol–gel, uncoated killed biofilms-CKB, and sol–gel-coated killed biofilms-CKB) were measured with SPME fiber passive samplers.

#### Preparation of Biofilm-CKB and Sol–Gel-Coated Biofilms

Method details of biofilm growth on CKB are found in our prior work^36^ and are summarized in S1.4 and S1.5. Biofilms were grown for 10 days in four parallel flasks (2 g CKB for each flask) to improve cell attachment and then combined to avoid growth variances among different batches. Initial experiments indicated that growing cells on a higher CKB mass (*e.g*., 7.5 g) in one flask would yield low cell attachment. To investigate the impacts of ancillary carbon sources on biofilm performance, biofilm-CKB was separated into two aliquots: one aliquot was soaked with 66 mM benzoate prior to sol–gel coating, while the other aliquot was not soaked (S1.4). Soaking biofilm-CKB with benzoate prior to sol–gel encapsulation is a strategy to load the PCB biodegradation (*bph* pathway) inducer onto CKB to potentially improve biofilm activity. Soaked and unsoaked biofilms were then distributed into aliquots for sol–gel coating. Each aliquot contained 0.12–0.13g of biofilm-CKB (wet weight).

Method details, including a schematic of sol–gel coating biofilm-CKB, are found in our prior work.^43^ Briefly, the sol solution was mixed with methyltriethoxysilane/tetraethyl orthosilicate/deionized water/poly­(vinylpyrrolidone) (MTES/TEOS/DI/PVP, molar ratio = 0.5:0.5:20:7.7 × 10^–6^) at pH = 1 for 6–7 h until hydrolysis was complete and residual ethanol evaporated. Glycerol or PEG400 (20% (v/v)) was then added to the sol solution to improve sol–gel coating completeness, control sol–gel pore size, and increase biocompatibility, followed by 30 s vortexing and potassium hydroxide (KOH) neutralization to pH = 7. Biofilm-CKB was mixed in neutral sol solution, filtered through a sterile tea ball, and aged for 1 day. Remaining glycerol/PEG400 is expected to be entrapped in sol–gel due to hydrogen bonding and confinement effects, with potential trace amounts of glycerol/PEG400 dissolving when added to solution.^41,54,55^ Dissolved glycerol can be used as a carbon source for LB400, while PEG400 may be less biodegradable.^56,57^ The two sol–gel recipes herein are henceforth referred to as “sol-gel_GLY_” and “sol-gel_PEG_”.

#### Experimental Design of PCB Biodegradation with Biofilm-CKB and Sol–Gel-Coated Biofilms under Standard Conditions

The experimental setup can be found in Figure S5. PCB biodegradation experiments included two control groups and two treatment groups. Controls included: (1) uncoated biofilms with benzoate soaking (“Biofilm Only+Soak”) and (2) sol–gel_GLY_-coated biofilms without benzoate soaking (“Sol-gel_GLY_+No Soak”). Two sol–gel-coated treatments were soaked with benzoate: sol–gel_GLY_ (“Sol-gel_GLY_+Soak”) and sol–gel_PEG_ (“Sol-gel_PEG_+Soak”). “Sol-gel_GLY_+No Soak” was compared with “Sol-gel_GLY_+Soak” to test the effects of benzoate soaking on PCB biodegradation performance. A “Sol-gel_PEG_+No Soak” treatment was not included to manage experimental size and experimental needs. Four time points were tested: *T*1 = 3 days, *T*2 = 14 days, *T*3 = 32 days, and *T*4 = 45 days. At each time point, 12 test tubes were sacrificed for PCB measurement (two treatments and two controls, *n* = 3 replicates per time point). Passive samplers (∼0.5 cm SPME fibers) were deployed to measure freely dissolved (or aqueous) PCB concentrations, with fiber length adjusted to maintain negligible depletion (<5% of total PCB mass). Equilibrium partitioning accounted for fiber polydimethylsiloxane (PDMS) volume, water volume, and sorbents. All solid samples (*i.e*., sol–gel-coated and uncoated biofilms-CKB) in each tube were extracted to determine PCB mass on sorbents.

PCB biodegradation experiments were conducted in 50 mL Pyrex test tubes containing 30 mL of 5 μg/L PCB mixture solution (PCBs 4, 18, and 52 in K1 medium, S1.5) and 0.42% mass loading (∼0.126 g, wet weight) of either coated or uncoated biofilms. Tubes for imaging purposes were constructed in parallel using identical procedures for PCB quantification tubes, except SPME fibers were omitted. To improve RNA/DNA extraction efficiency, we scaled up the volume of 5 μg/L PCB mixture solution (to 95 mL) and the mass of coated and uncoated biofilms (to ∼0.4 g, wet weight) in closed conical flasks for nucleic acid extraction. To determine the initial PCB concentration, 120 mL of the PCB mixture solution was added to 250 mL ASE collection bottles (*n* = 3 replicates) containing ∼2 cm SPME fibers, and SPME fibers were collected after 1-day shaking. All test tubes were placed on a tilted platform shaker at 30 rpm at 20–21 °C, and all tubes were sampled sacrificially.

#### Experimental Design of Environmental Condition Impacts on PCB Biodegradation

The effects of relevant environmental conditions, including dissolved oxygen (DO) level (fully aerobic to <6 mg/L), salinity (20 g/L), lower temperature (10 °C), and presence of natural organic matter (2 mg/L humic acid), on PCB biodegradation performance of sol–gel-coated biofilms were evaluated and compared with standard conditions (fully aerobic, freshwater, room temperature (20–21 °C)). Salinity, temperature, and humic acid were chosen to mimic real environmental sites (*e.g*., PCB-contaminated sites) to establish relevant environmental connections. Oxygen was added to the headspace of sealed serum bottles by sterile syringe after DO levels dropped below 6 mg/L, using the estimated half-velocity coefficient for oxygen (K_s_(O_2_)) for strain LB400 (S1.6, Figure S6). DO was measured using gas chromatography with thermal conductivity detection (GC-TCD) as previously described,^58^ with details in S1.6. For these five conditions, two treatment groups and one control group were prepared (*n* = 3 replicates) and are henceforth referred to as “sol-gel_GLY_”, “sol-gel_PEG_”, and “No Coating”. For each aliquot, biofilms-CKB (0.52–0.53 g wet weight) were either uncoated or coated with sol–gel and without benzoate soaking. All groups were shaken on the platform shaker at 140 rpm for 45 days.

PCB quantification was performed in closed 250 mL conical flasks or in sealed 250 mL serum bottles (for DO control and measurement) containing 125 mL of 5 μg/L PCB mixture solution (PCBs 4, 18, and 52) dissolved in pure K1 nutrient media, 20 g/L salinity in K1, or 2 mg/L humic acid in K1 medium and 0.42% mass loading (∼0.526 g, wet weight) of either coated or uncoated biofilms-CKB. At day-45, passive samplers (∼0.5 cm SPME fibers) were collected to measure aqueous PCB concentrations. A portion of the known biofilm-CKB mass (0.02–0.09 g, dry weight) was used to extract PCB mass, normalized by biofilm-CKB mass. Remaining biofilm-CKB was reserved for RNA/DNA extraction (∼0.4 g, wet weight) and imaging (∼0.05 g, wet weight). Initial PCB concentrations were measured separately by adding nominal 0.5 cm SPME fibers (length each measured; details in S1.2) to 150 mL of PCB master mix (5 μg/L PCB mixture in K1, 20 g/L salinity in K1, and 2 mg/L humic acid in K1), and SPME fibers were collected after at least 7 days of shaking. Initial sol–gel encapsulation conditions were assessed by imaging. Sol–gel-coated and uncoated biofilm samples were stored at −80 °C after encapsulation at day-0 for RNA/DNA extraction *(bphA* expression analyses).

### Analytical Methods

#### PCB Extraction and Quantification

PCB extraction details are in S1.7 and Table S1. Briefly, PCBs in solid samples (sol–gel-coated and uncoated biofilms-CKB collected at different time points) were extracted with acetone and hexane using an accelerated solvent extractor (ASE-350). Before extraction, solid samples were separated from liquid by pouring through a steel mesh (No. 50, 450 °C oven-heated), followed by air drying for ∼1 h. Dried solid samples were ground with activated diatomaceous earth (DE), spiked with ^13^C-labeled surrogate standards, and loaded into ASE cells. The sample extract from ASE was concentrated, cleaned through a silica gel column, and reconcentrated to a GC vial. The final extract was spiked with internal standards prior to PCB quantification. PCB quantification method details and quality assurance/quality control are found in prior work^15^ and S1.7.

#### Nucleic Acid Extraction and Reverse Transcription (RT)-qPCR

Method details of nucleic acid extraction and RT-qPCR are found in S1.8 and our prior work.^36^ Briefly, solid samples collected before (day-0) and after treatment (day-45) were flash-frozen with liquid nitrogen and stored at −80 °C prior to nucleic acid extraction. RNA/DNA for RT-qPCR was extracted with a modified RNeasy PowerSoil Total RNA kit (S1.8, Table S2), followed by the RNeasy PowerSoil DNA Elution kit. Luciferase control mRNA (1 ng = 6.08 × 10^9^ transcripts) was added to RNA extracts after cell lysis as an internal standard.^59,60^ RNA extracts were purified and concentrated with the RNA Clean & Concentrator kit. RNA was reverse transcribed to cDNA in 20 μL reactions (containing 11 μL of RNA, 50 ng of random hexamer, 10 mM dNTPs) using the SuperScript IV First-Strand Synthesis System. Qubit dsDNA HS Assay kits were used to quantify DNA concentrations with the Qubit 4 fluorometer.

The *bphA* abundance (DNA) and *bphA* and luciferase transcript abundance (cDNA) were measured by RT-qPCR using primer sets *bphA* 463*f*/674*r* and *ref* forward primer/*ref* reverse primer (nucleotide positions 1691/1758), respectively (Table S3).^59,61^ Transcript abundance was normalized to gene abundance to determine the transcript per gene ratio. Detailed RT-qPCR and QA/QC procedures and results are described in S1.8 and Figures S7–S8. Additional RT-qPCR information is provided in Table S4 to follow MIQE guidelines.^62^

#### Biofilm Viability and Sol–Gel Integrity

Biofilms on CKB, both sol–gel-coated and uncoated, were stained with SYTO9 and propidium iodide (details found in our prior study^43^ and S1.9). The distribution of live and dead LB400 biofilms on CKB, both before (day 0) and after treatment (day-45), was observed by confocal laser scanning microscopy (CLSM) with a Leica SP8 STED Super Resolution Confocal (Leica Microsystems, Exton, PA). Scanning electron microscopy (SEM) was used to observe sol–gel integrity on the biofilm-CKB surface before (day 0) and after treatment (day-45) using a Hitachi S-4800 (Tokyo, Japan) (S1.9).

### Statistical Analysis

Statistical analysis was conducted in GraphPad Prism 9 and Microsoft Excel. Data (*bphA* transcripts, *bphA* abundance, and transcript per gene ratio) were log_10_-transformed before statistical analysis. The ROUT outlier test was conducted, and two statistical outliers of DNA abundance (sol–gel_GLY_ coated biofilm-CKB at day-45 under standard conditions) were removed. PCB biodegradation rates were determined with a one-phase decay model in Prism. A two-sided *t* test, one-way ANOVA, or two-way ANOVA was applied to test for differences between treatment means depending on the experimental design, with Tukey’s post hoc test (as applicable) at the 95% confidence level (α = 0.05).

## Results and Discussion

### Sol–Gel Coating Improved Long-Term PCB Biodegradation Performance under Standard Conditions

Sol–gel_PEG_-coated biofilms exhibited comparable performance in biodegrading aqueous PCBs after 45 days compared to uncoated biofilms, despite presenting a physical (*i.e*., mass transport) barrier between the biofilm and bulk aqueous phase. Aqueous-phase PCB mass decreases in biotic treatments were >99%, while those in abiotic controls were around 89% (Figure 1-aqueous, killed biofilms), in which a 10% greater PCB mass decrease in biotic treatments was indicative of aqueous-phase PCB biodegradation. Sol–gel and CKB both demonstrated high PCB sorption potential (>80% of PCBs at equilibrium; Figure 1, S2.1, Figures S9 and S10, Table S5 and S6). The aqueous PCB mass increase in sol–gel_GLY_-coated biofilms (both soak and no soak), illustrated from day-14 to day-45 (Figure 1-aqueous), demonstrated that PCB sorption processes are reversible. Potential mechanisms may be that small amounts (<2%) of PCBs sorbed on sol–gel_GLY_ did not reach the biofilms and desorbed back into the aqueous phase. By using SPME fibers to quantify aqueous-phase PCB concentrations,^15,63,64^ uncoated biofilms (biofilm only+soak) efficiently sorbed and degraded all selected LC-PCBs in solution within 2 weeks, maintained low level–nearly zero–aqueous PCB concentrations, and demonstrated >6 times higher PCB biodegradation rates (*k*, d^–1^) than sol–gel-coated biofilms (both sol–gel_GLY_ and sol–gel_PEG_)­(*p* < 0.0003; S2.2, Table S7, Figures S11–S14).

**Figure 1:**
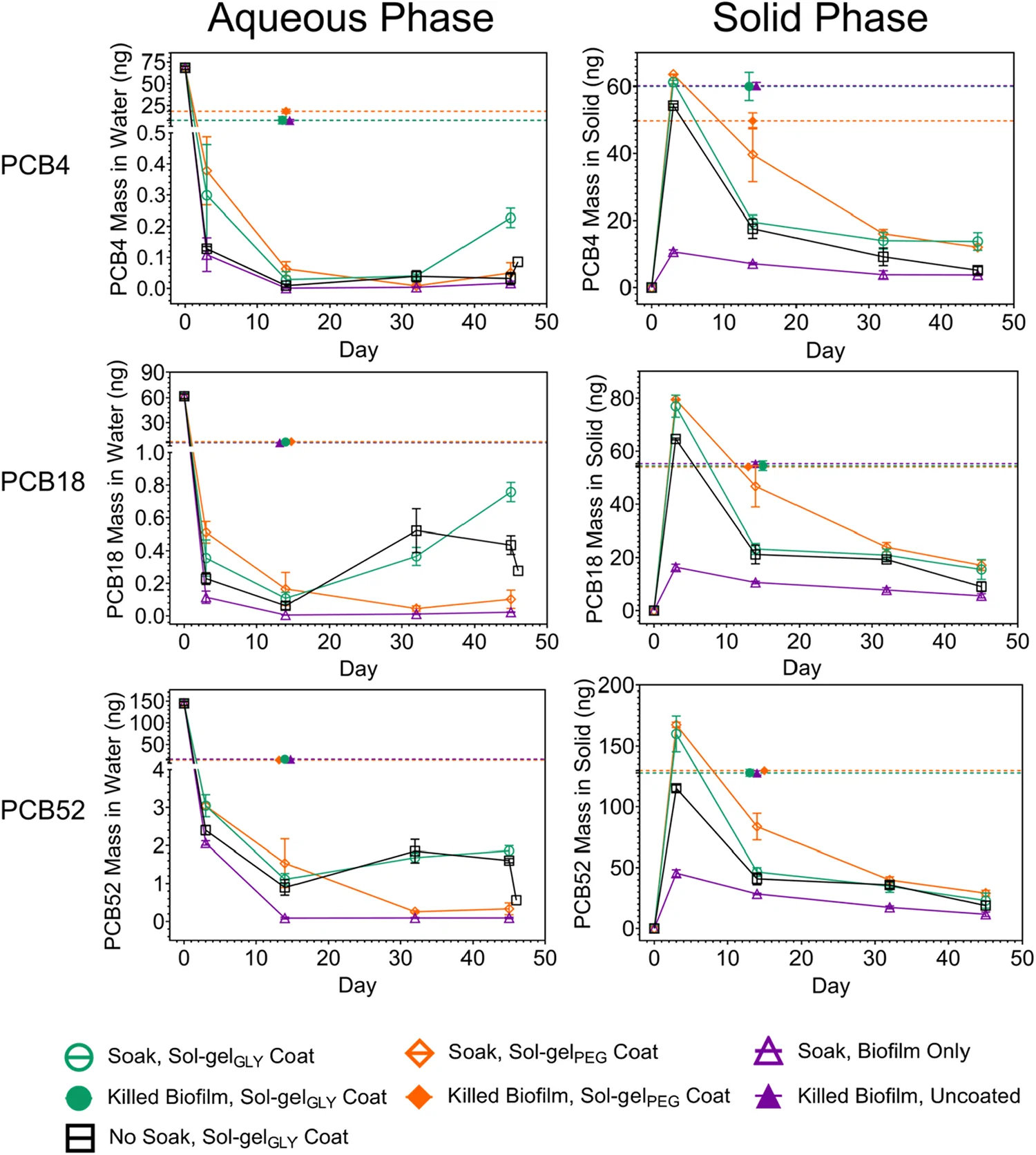
Changes in PCB congener mass over time in the aqueous (column 1) and solid (column 2) phases, in the presence of uncoated and sol–gel-coated LB400 biofilms on CKB: PCB 4 (row 1), PCB 18 (row 2), and PCB 52 (row 3). PCBs in both aqueous and solid phases were measured at each time point, where solid-phase PCBs were considered sorbed to materials (sol–gel and/or CKB). “Killed biofilm” indicates abiotic sorption controls corresponding to “benzoate soak + sol-gel_GLY_ coating/sol-gel_PEG_ coating/biofilm only”. Data points are the average of measurements from triplicate bottles (Data points are in Table S8), and the error bars are the standard error. Dashed lines in “aqueous phase” column represent remaining aqueous PCB mass in abiotic controls, and dashed lines in “solid phase” column represent sorbed PCB mass in abiotic controls, both of which were calculated based on *K*_d_ (Figure S10, Table S5) at sorption equilibrium, where *K*_d_ was determined in a separate parallel abiotic experiment in Figure S9. The differences between abiotic controls and biotic treatments were considered PCB biodegradation by biofilms. Note that some error bars are obscured by the datapoint symbols.

Compared with uncoated biofilms, sol–gel-coated biofilms (both sol–gel_GLY_ and sol–gel_PEG_) degraded LC-PCBs at lower initial rates; however, lower biodegradation rates due to mass transfer limitations from coating effects were negated over extended time periods (45-day experiment) and thus presented minimal long-term impacts on performance. Sol–gel_PEG_-coated biofilms exhibited a lower biodegradation-facilitated removal rate than sol–gel_GLY_-coated biofilms (PCB 4:0.04 d^–1^ *vs* 0.11 d^–1^, *p* = 0.0068, Table S7), potentially due to mass transfer efficiency (*i.e*., PCBs, oxygen) and capacity differences between sol–gel_GLY_ and sol–gel_PEG_ partially caused by sol–gel pore size differences between sol–gel_PEG_ and sol–gel_GLY_, but sol–gel_PEG_-coated biofilms achieved and maintained low levels of aqueous PCB mass, similar to uncoated biofilms, after 1 month (Figure 1-aqueous). Nevertheless, the total PCB mass biodegraded by sol–gel_GLY_-coated biofilms within the first 2 weeks was more than 77% higher than that observed between days 14 and 45, indicating lower PCB availability following rapid early degradation (>70% of total PCB removal) (Figure S15). Thus, there was evidence that sorption was synergistic with the biodegradation processes.

Mass removal of sorbent-associated PCB 4 and PCB 18 by both sol–gel_GLY_- and sol–gel_PEG_-coated biofilms was lower than uncoated biofilms over the entire treatment period (*p* < 0.03 for all groups except sol–gel_GLY_ with PCB 18 having *p* = 0.0516), with decreasing removal differences over time (Figure 1, solid). Sorbent-associated PCBs are defined as PCBs sorbed on materials (*i.e*., CKB and sol–gel) that were extracted and quantified as described in S1.7. Because the same initial PCB mass was used in all treatments, a higher remaining PCB mass on the sorbents indicates less sorbent-associated PCB biodegradation. Killed biofilms (coated and uncoated) were used as controls to demonstrate abiotic PCB sorption on cells, CKB, and/or sol–gel (Figure 1 solid, killed biofilms; Figures S9 and S10). Similarly, differences between sorbent-associated PCB 52 removal by sol–gel-coated (both sol–gel_GLY_ and sol–gel_PEG_) and uncoated biofilms gradually declined from 4-fold (day-3, *p* = 0.0001) to 2-fold (day-45, *p* > 0.05) (Figure 1, solid). A potential explanation is that sol–gel coating (both sol–gel_GLY_ and sol–gel_PEG_) provides mass transfer resistance to LC-PCBs from the aqueous solution to the interior coated biofilms, requiring additional time for LC-PCBs to pass through the porous sol–gel structure and reach biofilms after PCBs initially sorbed within the first 3 days. Nevertheless, when comparing total PCB sorption mass between plain CKB and combined pieces of sol–gel-coated CKB at equilibrium, we did not quantify significant differences in net sorption in batch tests (both sol–gel_GLY_ and sol–gel_PEG_; *p* > 0.17; Figure S9), likely due to small sample size. Estimating PCB sorption on individual sol–gel or CKB portions based on mass composition and *K*_d_ values also indicates that complete sol–gel coating (both sol–gel_GLY_ and sol–gel_PEG_) allows PCB transfer from the bulk liquid across the sol–gel and sorption to the CKB surface (Table S6). PCB sorption by sol–gel (both sol–gel_GLY_ and sol–gel_PEG_) was weaker than CKB (*K*_d_: plain sol–gel was 5–39% of plain CKB, Figure S10). Indeed, PCB sorption to CKB appeared to be hampered by biofilm attachment rather than by sol–gel (details in S2.1).

Uncoated biofilms without a mass transfer barrier posed by encapsulation degraded >67% of LC-PCB mass after 3 days of treatment, resulting in >91% of LC-PCB removal by day-45 (Figures 1 and S15). Nevertheless, without sol–gel protection, uncoated biofilms either lost many cells or contained mostly dead cells on the BC surface after 45-day treatment (Figure 2), which may explain the lower *bphA* expression levels compared to sol–gel-coated biofilms (Figure 3) rather than the lack of an available carbon/energy source. Thus, although uncoated biofilms demonstrated stronger LC-PCB initial biodegradation kinetics, we posit that the long-term biodegradation performance of sol–gel-coated biofilms would likely be greater based on the results presented herein on sustained biofilm cell abundance/viability and functional gene activity (*i.e*., *bphA* expression). Because cell viability often limits bioaugmentation success as a remediation strategy under environmental conditions,^15,37,38,64^ approaches to sustain cell abundance and activity may be more important than initial biodegradation kinetic limitations; however, these questions will require future research efforts at scaling in complex systems to verify.

**Figure 2:**
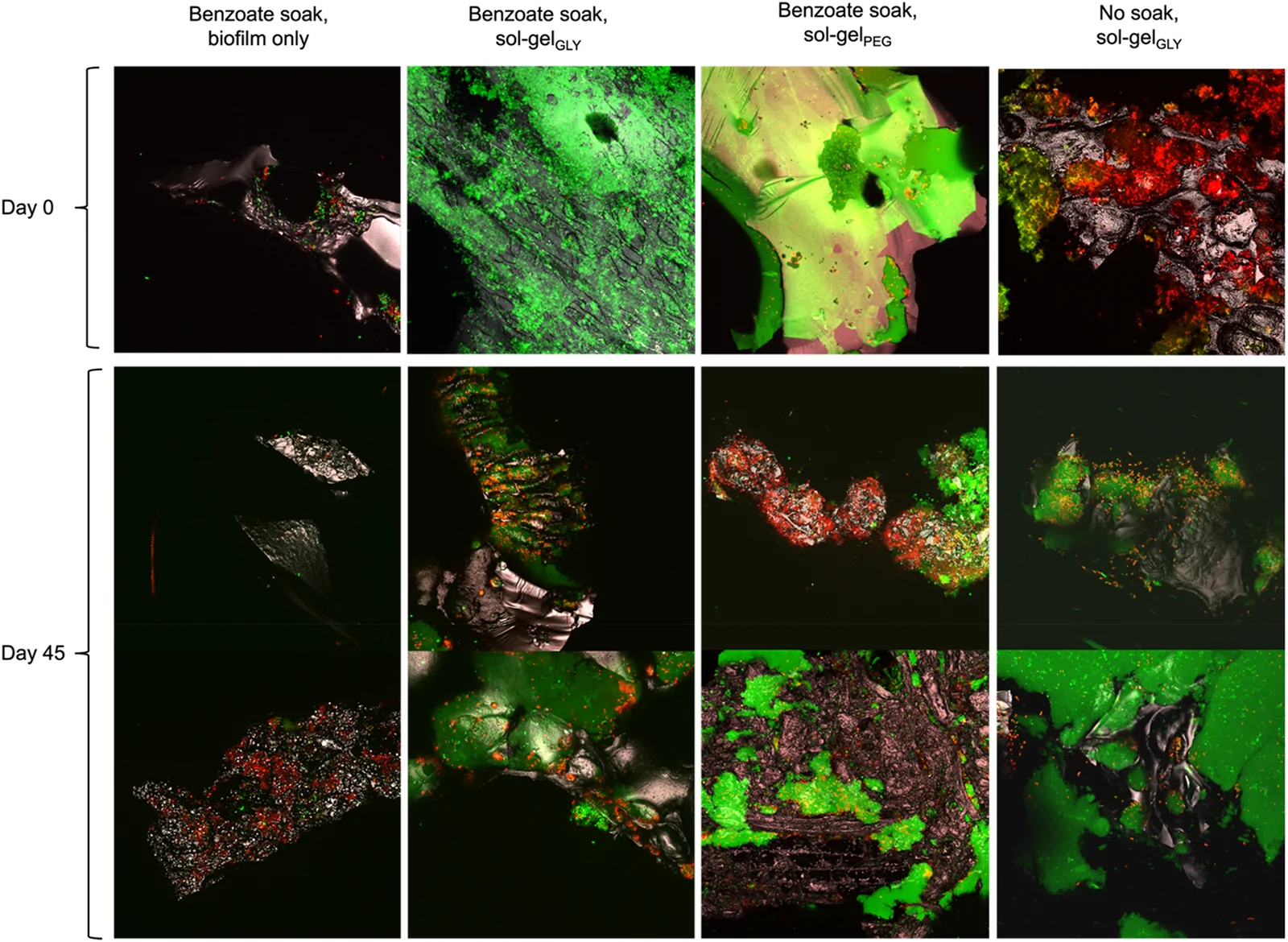
Confocal laser scanning microscopic images: surface of sol–gel-coated and uncoated biofilms before (day-0) and after shaking (day-45) in the presence of PCBs under standard conditions. Four columns represent 4 groups: 1st column represents “benzoate soak + biofilm only”; 2nd column represents “benzoate soak + sol-gel_GLY_”; 3rd column represents “benzoate soak + sol-gel_PEG_”; 4th column represents “no soak + sol-gel_GLY_”. The two rows presented for day-45 show images of biological duplicates. Red dots represent dead cells, green dots represent live cells, and the continuous green fluorescence represents the sol–gel coating. All images had the same magnification.

**Figure 3:**
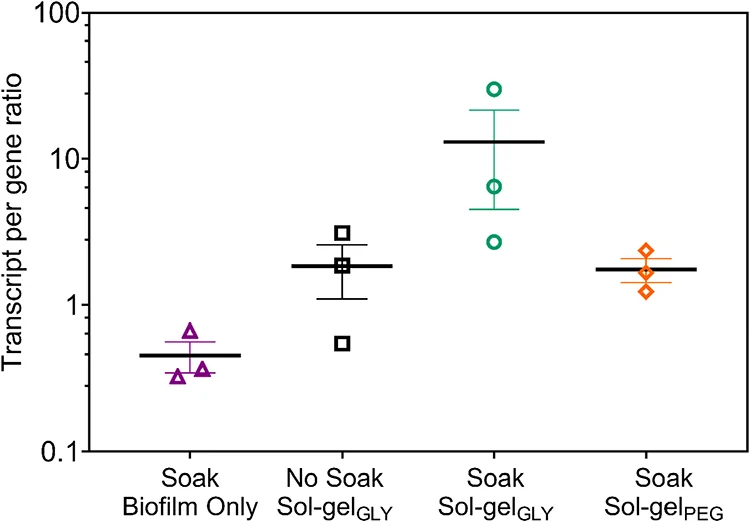
*BphA* expression levels (transcript per gene ratios = transcript abundance/gene abundance) of sol–gel-coated and uncoated biofilms under standard conditions at day-45. Purple triangles represent “benzoate soak + biofilm only” controls; black squares represent “no soak + sol-gel_GLY_” controls; green circles represent “benzoate soak + sol-gel_GLY_” treatments; and orange diamonds represent “benzoate soak + sol-gel_PEG_” treatments. Black lines represent the mean of biological replicates (*n* = 3), and error bars represent the standard error.

Sol–gel_GLY_-coated biofilms exhibited significantly more PCB congener biodegradation (*p* < 0.04 for PCB 18 and PCB 52) than sol–gel_PEG_-coated biofilms during the initial treatment time (<14 d). Specifically, 62–67% of PCB 18 (contains 3 chlorines) and PCB 52 (contains 4 chlorines) were degraded by sol–gel_GLY_-coated biofilms within 14 days, while only 24–41% were degraded by sol–gel_PEG_-coated biofilms (Figure S15). Differences were negated, however, for PCB 4 (contains 2 chlorines), where significantly higher total mass removal by sol–gel_GLY_-coated biofilms than sol–gel_PEG_-coated biofilms only occurred within 3 days (*p* = 0.009). Total PCB mass was quantified as the sum of aqueous PCBs measured by SPME fibers^15,63,64^ and sorbent-associated PCBs by sorbent extraction. We hypothesize that the temporary total PCB mass biodegradation differences within 14 days could be related to two potential mechanisms: (1) PCB sorption efficiency and capacity differences between sol–gel_GLY_-coated CKB and sol–gel_PEG_-coated CKB (Figure S10, Table S5) and (2) potential glycerol leaching from the outer surface of sol–gel_GLY_ served as a carbon source, as indicated by oxygen uptake in sol–gel_GLY_ (Figure S20). Increasing the chlorine number for PCBs increases the molecule hydrophobicity, causing greater sorption by biochar.^65^ Additionally, narrow pore sizes increase sorption onto BC;^66^ here, the sol–gel_GLY_-coated CKB (>4.6 nm radius) pore size was larger than that of sol–gel_PEG_-coated CKB (1.62 nm radius). Taken together, these aforementioned factors potentially explain why LC-PCBs with more chlorines sorbed more strongly to sol–gel_PEG_-coated CKB. Total PCB mass decrease by sol–gel_GLY_-coated and sol–gel_PEG_-coated biofilm-CKB biodegradation (Figure S15) also indicates that sorption to sol–gel and CKB would not materially interfere with PCB mass transfer to encapsulated biofilms.

From a longer-term perspective (45 days), aqueous PCB biodegradation (quantified by SPME fibers) with sol–gel_PEG_-coated biofilms was not different (*p* > 0.05) from uncoated biofilms, while sol–gel_PEG_-coated biofilms exhibited superior aqueous PCB biodegradation extent (*p* < 0.005) than sol–gel_GLY_-coated biofilms. Although 45 days is a relatively short period for *in situ* PCB bioremediation (compared to a one-year study^38,39^ ), 45 days is an environmentally relevant period in the context of relatively fast aerobic LC-PCB biodegradation. Based on Henry’s law, we estimated the amounts of PCB volatilization to headspace of test tubes (S2.4), and under the worst-case scenario, the maximum PCB volatilization was only ∼0.8% of spiked PCBs (Figure S15). The potential PCB volatilization loss during sample extraction was determined to be negligible in preliminary experiments (Table S1). Therefore, our study demonstrated that high percentages of total PCB loss were due to biofilm biodegradation rather than volatilization.

### Sol–Gel Coating Enhanced Biofilm Integrity and Viability, and Extended PCB Biodegradation Activity of LB400 Biofilms for Long-Term Treatment

Sol–gel-coated and uncoated biofilms were visualized by SEM and CLSM imaging after completing the sol–gel coating process (day-0 images) and after the PCB biodegradation assay (day-45 images). Prior to PCB exposure treatment (Figure 2, S2.3, Table S9, day-0), CLSM imaging demonstrated that sol–gel-coated biofilms (both sol–gel_GLY_ and sol–gel_PEG_ coatings) contained 3–8 times more total cells on the CKB surface than uncoated biofilms. Using SEM imaging, both sol–gel_GLY_ and sol–gel_PEG_ coatings CKB surfaces were well covered with a highly porous structure (Figure S16), while the sol–gel_PEG_ coating was less porous than the sol–gel_GLY_ coating at higher magnifications (*e.g*., 5 and 50 μm). More LB400 cells were observable on sol–gel_GLY_-coated CKB, consistent with CLSM results that sol–gel_GLY_-coated biofilms held >2 times more total cells than the sol–gel_PEG_-coated biofilms; this phenomenon may be because rougher sol–gel spheres were spread with the sol–gel_PEG_ coating (*e.g*., 400 μm scale).^67^

After 45 days of treatment under standard conditions (*i.e*., freshwater, continuous shear force by shaking at a tilted angle at 30 rpm, room temperature), both sol–gel_GLY_- and sol–gel_PEG_-coated biofilms maintained gel integrity and had higher biofilm integrity than uncoated biofilms. CLSM imaging demonstrated that cells in uncoated biofilms were mostly dead (∼2:3 live to dead cells) or had detached from the CKB at day-45 (Figure 2), potentially because the lack of physical protection did not prevent biofilm detachment and/or the lack of a carbon/energy source after a few days led to increased cell death.^43^ Sol–gel_GLY_ and sol–gel_PEG_ (+soak) treatments had 1.5–3.8 times more total cells, where most remaining cells were under the sol–gel, and more live cells (2.4:3 live to dead cells) compared to uncoated biofilms (Table S9, day-45). The sol–gel_GLY_ coating maintained higher porosity than the sol–gel_PEG_ coating after treatment, but more LB400 cells were embedded within the sol–gel_PEG_ coating (Figures S17–S18). Higher cell abundances under the sol–gel_PEG_ coating compared to sol–gel_GLY_ may relate to sol–gel hardness and roughness, where sol–gel_PEG_ had higher hardness and rougher protection compared to sol–gel_GLY_ at day-0.^67^ This observation highlights the importance of sol–gel coverage and roughness to mitigate biofilm detachment over longer periods (at least 45 days), whereas soaked benzoate was rapidly consumed and the benefit was limited.

PCB biodegradation gene (*bphA)* expression levels (*i.e*., transcripts and transcript/gene ratios) were elevated in sol–gel-coated biofilms prior to PCB treatment, compared to uncoated biofilms. At day 0, biofilms on CKB exhibited active *bphA* expression levels (*i.e*., transcript/gene ratio >1), with enhanced *bphA* expression levels in sol–gel-coated biofilms with benzoate soaking. Uncoated biofilms had two times higher *bphA* transcripts than both sol–gel-coated treatment groups (*p* < 0.0001 for both coatings; Figure S19a) and 2 orders of magnitude higher *bphA* abundance than sol–gel_PEG_-coated biofilms (*p* = 0.0096; Figure S19b). Therefore, sol–gel-coated biofilms (+soak) on average expressed at least 2 orders of magnitude more *bphA* transcripts per gene (*p* > 0.05) than uncoated biofilms (+soak)­(Figure S19c) prior to PCB treatment. For sol–gel_GLY_, soaking biofilm-CKB with benzoate had more active biofilms (over 3 orders of magnitude higher, *p* > 0.05) than no-soaking groups.

After 45 days of PCB treatment under standard conditions, both sol–gel_GLY_- and sol–gel_PEG_-coated biofilms were more active (*bphA* transcript per gene ratio >1) than uncoated biofilms (Figure 3). Sol–gel_PEG_-coated biofilms had at least 2 times higher *bphA* expression levels than uncoated biofilms (*p* = 0.0196). Based on our prior study^43^ and current estimation of sorbed benzoate on biofilm-CKB (8.5 μmol), we expect that benzoate was rapidly consumed by both sol–gel-coated and uncoated biofilms. Therefore, elevated *bphA* expression in sol–gel_PEG_-coated biofilms compared to uncoated biofilms was not due to benzoate availability but rather sol–gel protection effects. Although sol–gel_GLY_-coated biofilms had 31–93% more *bphA* transcripts and ∼10 times higher gene expression levels (transcript per gene ratio) than sol–gel_PEG_-coated and uncoated biofilms (Figure S19d,f), large variance within sol–gel_GLY_-coated biofilm groups obscured potential statistical significance. In contrast to day-0, the *bphA* transcript abundance at day-45 of both sol–gel_GLY_-coated and sol–gel_PEG_-coated biofilms was significantly higher (about 10 times) than uncoated biofilms (*p* < 0.0001; Figure S19d), while the *bphA* abundance of sol–gel_GLY_-coated and uncoated biofilms was at least 2 times lower than sol–gel_PEG_-coated biofilms (*p* < 0.03; Figure S19e). Therefore, sol–gel_PEG_ helped maintain *bphA* expression in biofilms compared to no coating. Although soaking with benzoate yielded 61% more *bphA* transcripts (*p* = 0.025) and 10 times higher expression levels than no soaking, there was no significant impact (PCBs 18 and 52, *p* > 0.05) on PCB biodegradation extent and, in one case, lower total PCB removal (PCB 4, *p* = 0.035) after 45 days (Figure 1 and Section S2.2).

By comparing each group between day-0 and day-45, an important finding is that all sol–gel-coated groups, regardless of soaking status and sol–gel recipe, had 75–95% more *bphA* transcripts after 45 days of treatment (*p* < 0.02), while uncoated biofilms had 1.5 times less *bphA* transcripts (*p* < 0.0001; Figure S19a,d). Additionally, only sol–gel_PEG_-coated biofilms demonstrated 3 orders of magnitude higher *bphA* abundance (*p* < 0.0001) at day-45, while all other groups maintained similar *bphA* abundance (*p* > 0.05) over 45 days (Figure S19b,e). Although high variance within biological replicates obscured any statistical differences in transcript per gene ratio (*p* > 0.05) before and after treatment, large changes in mean *bphA* expression level over time were still observed and important. For example, the *bphA* expression level in uncoated biofilms decreased by 20 times after 45 days. Both sol–gel_PEG_- and sol–gel_GLY_-coated biofilms (+soaking) showed at least 100-fold *bphA* expression level decreases over time, but maintained *bphA* transcript/gene ratios >1 after 45 days (Figure S19c,f). Interestingly, *bphA* gene expression levels in sol–gel_GLY_-coated biofilms (without benzoate soaking) increased over 45 days, which is consistent with the oxygen consumption observed in sol–gel_GLY_-coated biofilms (Figure S20) and further indicates that an auxiliary carbon source may not be necessary to maintain *bphA* expression in sol–gel_GLY_-coated LB400 biofilms. To summarize, sol–gel coating maintained (and possibly induced) *bphA* expression in LB400 biofilms over 45 days under freshwater and room temperature (20–21 °C) conditions. Although the *bphA* abundance of uncoated biofilms on CKB was maintained over 45 days (Figure S19b,e), uncoated biofilms could not maintain *bphA* expression for 45 days without sol–gel protection, compared to sol–gel-coated biofilms.

### Environmental Conditions and Sol–Gel Coatings Significantly Affected PCB Biodegradation and PCB-Degrading Biofilm Gene Expression

Sol–gel coating and temperature were key factors influencing sorbent-associated PCB biodegradation. Sol–gel coating type and environmental conditions (*i.e*., temperature (10 °C), variable DO (Figure S20), salinity (20 g/L), presence of humic acids (2 mg/L)) independently (*p* = 0.1) and significantly affected sorbent-associated PCB biodegradation (*p* < 0.0001). Across all tested environmental conditions, sorbent-associated PCB biodegradation was significantly higher in uncoated biofilms compared to sol–gel_PEG_-coated biofilms (2.3-fold more PCB mass remaining in sol–gel_PEG_-coated biofilms, *p* < 0.0001) but not significantly different from sol–gel_GLY_-coated biofilms (*p* = 0.175; Figure 4d–f). This result indicates that sorbent-associated PCB biodegradation by sol–gel_GLY_-coated biofilms was comparable to uncoated biofilms at all environmental conditions, while sol–gel_PEG_-coated biofilms were less effective. A potential driver for differences observed between sol–gel_PEG_-coated and sol–gel_GLY_-coated biofilms is that a small amount of glycerol leached from the outer surface of sol–gel^68^ although the majority of incorporated glycerol/PEG400 was expected to remain by hydrogen bonding and confinement effects.^54,55^ LB400 uses glycerol as a carbon energy source,^56^ but there are no reports that it can utilize PEG400. Polymeric PEG400 is biodegradable but generally microbially recalcitrant compared to microbially labile glycerol.^57,69,70^

**Figure 4:**
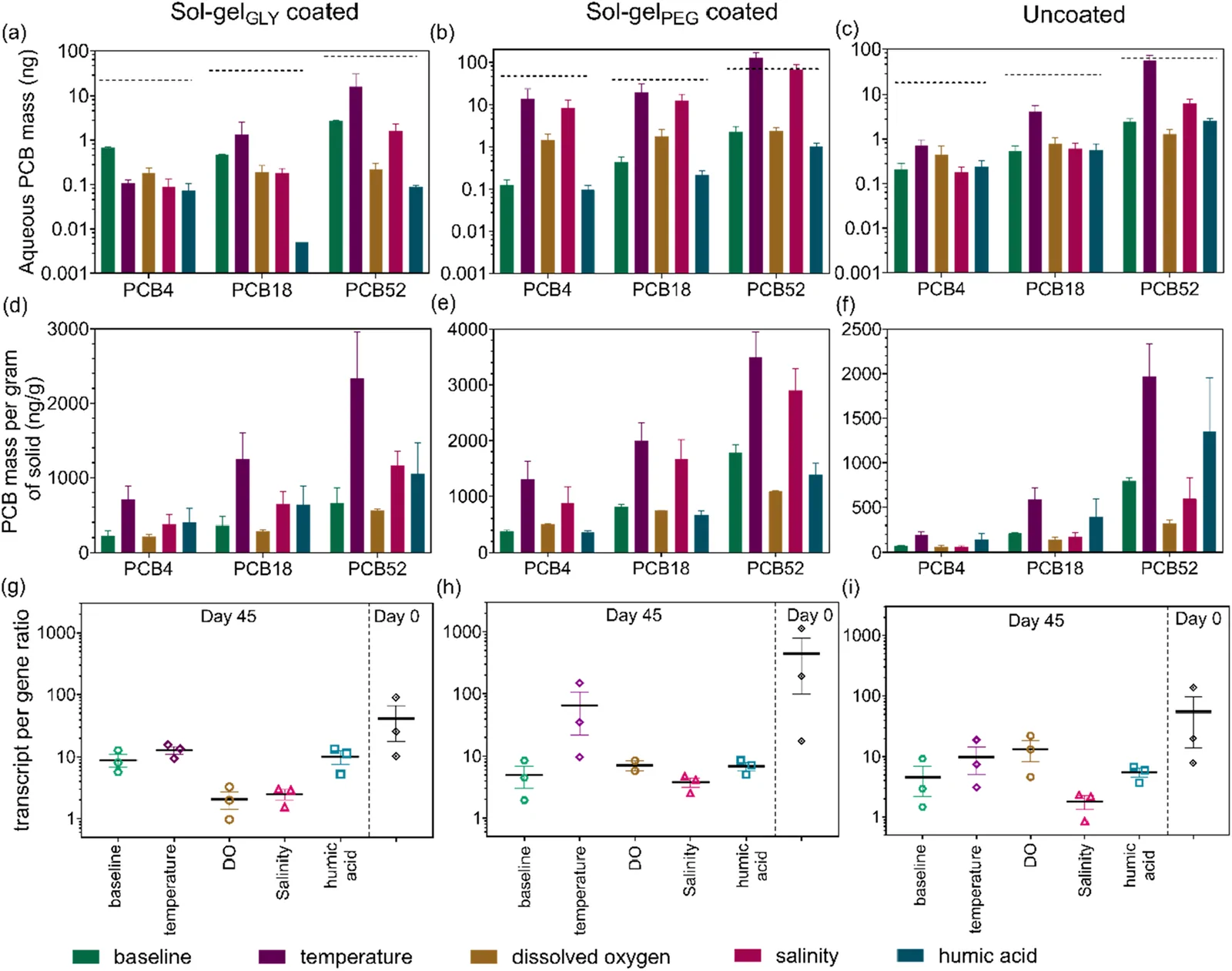
PCB (PCBs 4, 18, and 52) biodegradation performance and *bphA* expression levels under different environmental conditions. Each column represents different biofilm-CKB sol–gel coating conditions: sol–gel_GLY_-coated, sol–gel_PEG_-coated, and uncoated. (a–c) Total aqueous PCB mass (ng, note log_10_ scale) in the reactor after 45 days, and horizontal dashed lines refer to abiotic controls of sol–gel-coated or uncoated killed biofilm-CKB in Figure S9 and represent remaining PCB mass of abiotic controls in aqueous phase after sorption reached equilibrium. (d–f) Quantification of PCB mass measured on sorbents normalized by sample mass (ng of PCBs per g of solid). (g–i) *bphA* expression levels (transcript per gene ratios, log_10_ scale) of sol–gel-coated and uncoated biofilms at day-0 and day-45 under different environmental conditions. Column bars in panels (a–f) represent the mean of three biological replicates, and error bars represent the standard error. Black lines in panels (g–i) represent the mean of three biological replicates (*n* = 3), with each biological replicate also containing three technical replicates, and error bars represent the standard error. Abiotic controls with uncoated and sol–gel-coated killed biofilms can be found in Figure S21.

Temperature was the most important factor among all tested environmental conditions in negatively influencing sorbent-associated PCB biodegradation. Sorbed PCB mass remaining at 10 °C was about 3-fold higher than at the baseline temperature (20–21 °C) (*p* < 0.0001), indicating less PCB biodegradation. Sol–gel cannot protect biofilms against temperature changes, and lower temperatures could potentially decrease PCB diffusion; however, sol–gel-coated biofilms maintained activity, whereas biofilms without sol–gel protection exhibited decreased activity at 10 °C. Environmental conditions besides temperature had no significant effect on sorbent-associated PCB biodegradation compared to baseline controls (*p* > 0.20), regardless of encapsulation.

Environmental conditions and sol–gel coating significantly (*p* < 0.0042) and interactively (*p* = 0.023) affected aqueous-phase PCB biodegradation. Environmental conditions (*i.e*., humic acids, DO, and temperature) significantly affected aqueous PCB biodegradation/sorption by sol–gel_PEG_-coated and uncoated biofilms (*p* < 0.016). Specifically, lower temperature negatively impacted aqueous PCB biodegradation/sorption by sol–gel_PEG_-coated and uncoated biofilms compared to baseline conditions (*p* < 0.04; Figure 4a–c). This phenomenon is consistent with our hypothesis that lower temperature decreased PCB sorption on hydrophobic gels and the activity of uncoated biofilms. Sol–gel coatings also significantly affected aqueous PCB biodegradation/sorption at different environmental conditions (*p* < 0.02). For both variable DOs and 20 g/L salinity, sol–gel_PEG_-coated biofilms biodegraded/sorbed significantly less aqueous PCBs than uncoated biofilms (*p* < 0.027) and sol–gel_GLY_-coated biofilms (*p* < 0.018). There were 10- to 93-fold more aqueous PCBs remaining in sol–gel_PEG_-coated biofilms than sol–gel_GLY_-coated and uncoated biofilms in the 20 g/L salinity treatment (Figure 4a–4c). Aqueous PCB biodegradation with 2 mg/L humic acid was higher in sol–gel_GLY_-coated biofilms than uncoated biofilms (*p* = 0.017). These findings indicate that the sol–gel_GLY_ recipe provides more efficient biofilm protection against the impacts of aqueous components like salt and dissolved organic matter (DOM) compared to no sol–gel coating or sol–gel_PEG_, whereas the sol–gel_PEG_ recipe provided less resistance to negative impacts from salinity, causing low aqueous PCB biodegradation/sorption. Differences between abiotic controls (dashed lines in Figure 4a–4c) and biotic treatments indicated PCB biodegradation.

Environmental conditions were the main factor (*p* < 0.0001) affecting *bphA* expression levels in LB400 biofilms, which effects depended on sol–gel coating type (*p* = 0.018). Levels of *bphA* expression were significantly higher under low-temperature conditions (10 °C) compared to other environmental conditions in sol–gel-coated biofilms after 45 days (*p* < 0.03; Figure 4g-h). Sol–gel_GLY_-coated biofilms at 10 °C had 5-fold higher *bphA* expression levels than variable DOs and 20 g/L salinity (*p* < 0.034), and sol–gel_PEG_-coated biofilms at 10 °C had 12-fold higher *bphA* expression levels than baseline temperature (20–21 °C; *p* = 0.042). This finding contrasts our observations of decreased PCB biodegradation extent in sol–gel-coated biofilms at 10 °C (Figure 4a–c). It is reasonable to expect that lower temperatures will decrease biofilm activity^71^ and concomitantly decrease PCB biodegradation extent, but we suggest that sol–gel coating maintained or even induced biofilm activity at 10 °C. Our findings are consistent with other studies that Gram-negative cells (*i.e*., LB400) with immobilization (+glycerol) were resistant to low temperature (4 °C) and maintained cell viability,^72^ with even more viable cells present at lower temperatures.^73,74^ Temperature also affected encapsulant microstructure rearrangement and phase transition.^74,75^ Depending on encapsulant material chemical properties, lower temperatures could yield higher surface area (*e.g*., hydrophilic encapsulant^75^) or higher mechanical strength (*e.g*., hydrophobic organogel^74^) encapsulants. More compact structures resulted in decreased oil absorption efficiency.^74^ This potentially explains how a lower temperature led to lower PCB diffusion/sorption through our hydrophobic sol–gels.

We found that *bphA* expression levels were at least 1 order of magnitude lower in all 20 g/L salinity groups on day-45 compared to day 0 (*p* < 0.0001, Figure 4g–4i). The observed salinity effects on *bphA* expression levels are consistent with other reports of decreased PCB biodegradation efficiency and PCB metabolite decomposition at higher salt concentrations, with different PCB-degrading aerobes showing different salt tolerances.^76,77^ Increased osmolarity could possibly lead to changes in cell wall structure and components (*e.g*., relative proportions of fatty acids), which may impact cell membrane permeability.^76^ Higher salinity may also decrease polymer gel volume.^78^ Although the precursors were different from our study, it is still possible that 20 g/L salinity led to shrinkage in sol–gels containing glycerol or PEG400. Sol–gel_GLY_ had a larger porous volume than sol–gel_PEG_ (0.022 *vs* 0.008 cm^3^/g) before treatment in saline solutions; thus, sol–gel_GLY_ may be less vulnerable to shrinkage than sol–gel_PEG_. This explanation is consistent with the greater observed negative impacts of salinity on aqueous PCB biodegradation/sorption in sol–gel_PEG_-coated biofilms than those in sol–gel_GLY_-coated biofilms.

Decreased *bphA* expression levels were more pronounced in sol–gel_PEG_-coated biofilms than in sol–gel_GLY_-coated biofilms over time (Figure 4g–h), where sol–gel_PEG_-coated biofilms exhibited a > 60-fold decrease in *bphA* expression levels under all environmental conditions except temperature (*p* < 0.045), while sol–gel_GLY_-coated biofilms exhibited <20-fold decreases under variable DO changes and 20 g/L salinity (*p* < 0.0012). This difference supports our hypothesis that potential glycerol leaching from the outer surface of sol–gel_GLY_ may act as an energy source, while PEG400 was less biodegradable,^57,69,79^ although minimal glycerol leached and most of the glycerol was expected to remain within sol–gel_GLY_ because of hydrogen bonding and confinement effects.

Sol–gel coating improved long-term biofilm abundance/viability. Both sol–gel coatings helped biofilms maintain biofilm integrity compared to uncoated biofilms (*p* < 0.03, S2.6, Figures S22 and S23, day-45). Sol–gel_GLY_-coated biofilms appeared less vulnerable to environmental changes and contained more biomass and viable cells than sol–gel_PEG_-coated biofilms, as supported by *bphA* abundance estimates (Figure S22) and microscopic images (Figures S23–24). Sol–gel_PEG_-coated biofilms had significantly lower *bphA* abundance compared to baseline conditions (*p* < 0.017; Figure S22d), 10–75% less total cells compared to sol–gel_GLY_-coated biofilms (Tables S10–11), and a thinner coating than sol–gel_GLY_ (Figure S23) under all environmental conditions except 2 mg/L humic acid. Cell abundance was affected by sol–gel coverage and thickness, where a thicker sol–gel had more cells underneath than a thinner sol–gel (S2.6, Figure S23). Multiple lines of evidence (*bphA* RT-qPCR, SEM and CLSM images) demonstrate that sol–gel coating maintained or even enhanced biofilm abundance and *bphA* transcription under 2 mg/L humic acid (S2.6, Figures S22–S24, Tables S10–S11), consistent with other findings that humic substances induce enzyme activity.^80^ Low humic acid concentrations can reportedly enhance gel strength by coagulation, but higher concentrations could lead to a weaker gel.^81^ Although we used a different gel precursor, it is possible that 2 mg/L humic acid (0.0002%) could increase sol–gel strength and thus maintain high biofilm abundance. These findings demonstrate the applicability implications of sol–gel for protecting biofilms against adverse environmental conditions.

### Environmental Implications

Sol–gel coating was critical in enhancing PCB biodegradation activity potential over time periods tested, as supported by multiple lines of evidence. Under standard conditions, sol–gel coating–especially sol–gel_PEG_ with benzoate soaking–induced and maintained higher *bphA* expression levels, consistent with findings that sol–gel encapsulation can enhance enzyme activity.^41,42^ Consistent with our prior study,^43^ we demonstrated at the bench scale that sol–gel coating protected biofilm integrity for long-term application, while uncoated biofilms decreased biofilm viability and abundance. Although sol–gel coating inhibited short-term PCB biodegradation rates due to mass transfer limitations, total PCB removal by sol–gel-coated biofilms was equivalent for coated and uncoated biofilms. Indeed, the sol–gel material itself was weakly sorptive, which facilitated sorption and desorption with subsequent biodegradation of PCBs between the bulk aqueous solution and encapsulated biofilm. Biodegradable carbon sources (*e.g*., benzoate addition by soaking or glycerol leaching from sol–gel_GLY_) could act as energy sources for PCB biodegradation and could impact short-term biodegradation performance;^79^ however, these factors are expected to contribute minimally (*i.e*., sol–gel could retard compound desorption while carbon source (*e.g*., benzoate) is rapidly consumed), and are beyond the scope of this present study that focused on environmental factors tested at the lab batch scale. Sol–gel not only protected biofilm abundance and viability, but also enhanced PCB biodegradation gene expression levels through the duration of the experiments. Because extended viability of cells likely determines the success of bioaugmented organisms in the field,^37,38^ approaches that maintain bioactivity hold the potential to improve longer-term biodegradation during eventual remediation deployment; however, extended viability in the field would have to be evaluated in future work.

This study also evaluated the impacts of environmentally relevant conditions on PCB biodegradation performance of sol–gel-coated biofilms, contributing new knowledge toward long-term future understanding of *in situ* application and feasibility of the sol–gel encapsulation design. Sol–gel_GLY_ outperformed sol–gel_PEG_ in overall biodegradation performance (*e.g*., PCB mass removal, *bphA* expression, and biofilm abundance) under adverse environmental conditions. Additionally, the sol–gel coating protected biofilm activity under lower temperature and higher humic acid but was not beneficial at higher salinity. Future work should include an assessment of the impact of sediments on sol–gel-encapsulated biofilms on corn kernel biochar at batch microcosm and flow-through mesocosm scales and eventually expand toward the field scale. Our sol–gel design demonstrates the capability to induce and prolong biofilm activity and abundance for the goal of enhancing sediment deployment of aerobic PCB-degrading organisms, ultimately with the long-term outcome of improving LC-PCB bioremediation and mitigating the exposure pathway from sediment to air.

## Supplementary Material



## Data Availability

Raw and processed PCB and RT-qPCR data are available under DOI: https://iro.uiowa.edu/esploro/outputs/dataset/9985136443702771.^82^

## Notes

PCB analysis methods, chemicals, and mass balance (Sections S1.1–S1.7), SPME data processing and analysis (Figures S1–S4), experimental design (Figure S5), LB400 cell oxygen consumption (Figure S6), nucleic acid extraction and RT-qPCR (Section S1.8, Tables S2–S4, Figures S7–S8), PCB sorption and PCB biodegradation rate results (Tables S5–S8, Figures S9–S15 and S21), oxygen consumption in uncoated and sol–gel-coated biofilms (Figure S20), biofilm viability data and microscopy images (Section S1.9, Table S9–S11, Figures S16–S18, S23–S24), and *bphA* abundance and expression data (Sections S2.5-S2.6, Figures S19 and S22) (PDF)

## References

[1] Adrian L., Dudková V., Demnerová K., Bedard D. L. (2009). *Dehalococcoides*”
sp. strain CBDB1 extensively dechlorinates the commercial
polychlorinated biphenyl mixture Aroclor 1260. Appl. Environ. Microbiol..

[2] Fusi, S. C. ; Chan, A. Y. ; Kjellerup, B. V. Processes of Microbial Transformation and Physical Removal of Polychlorinated Biphenyls (PCBs) in Wastewater Treatment. In Optimization and Applicability of Bioprocesses; Springer, 2017; pp 101–113 10.1007/978-981-10-6863-8_5.

[3] Achman D. R., Hornbuckle K. C., Eisenreich S. J. (1993). Volatilization of polychlorinated
biphenyls from Green Bay, Lake Michigan. Environ.
Sci. Technol..

[4] Hoff R., Strachan W., Sweet C., Chan C., Shackleton M., Bidleman T., Brice K., Burniston D., Cussion S., Gatz D. (1996). Atmospheric deposition
of toxic chemicals to the Great Lakes: a review of data through 1994. Atmos. Environ..

[5] Hornbuckle K. C., Achman D. R., Eisenreich S. J. (1993). Over-water
and over-land polychlorinated
biphenyls in Green Bay, Lake Michigan. Environ.
Sci. Technol..

[6] Hornbuckle K. C., Jeremiason J. D., Sweet C. W., Eisenreich S. J. (1994). Seasonal
variations in air-water exchange of polychlorinated biphenyls in Lake
Superior. Environ. Sci. Technol..

[7] United States Environmental Protection Agency . Learn about Polychlorinated Biphenyls (PCBs), https://www.epa.gov/pcbs/learn-about-polychlorinated-biphenyls-pcbs (accessed May 19, 2022).

[8] Montano L., Pironti C., Pinto G., Ricciardi M., Buono A., Brogna C., Venier M., Piscopo M., Amoresano A., Motta O. (2022). Polychlorinated biphenyls
(PCBs)
in the environment: occupational and exposure events, effects on human
health and fertility. Toxics.

[9] Li X., Hefti M. M., Marek R. F., Hornbuckle K. C., Wang K., Lehmler H.-J. (2022). Assessment of polychlorinated
biphenyls
and their hydroxylated metabolites in postmortem human brain samples:
age and brain region differences. Environ. Sci.
Technol..

[10] Wang H., Adamcakova-Dodd A., Lehmler H.-J., Hornbuckle K. C., Thorne P. S. (2022). Toxicity assessment of 91-day repeated inhalation exposure
to an indoor school air mixture of PCBs. Environ.
Sci. Technol..

[11] Davis, B. ; Wade, M. Risk Assessment of Polychlorinated Biphenyls at Hazardous Waste Sites; DTSC, 2003.

[12] Michaels R.
A., Oko U. M. (2017). Excessive
PCBs in the Hudson river: attributable to
incompleteness of dredging, or to seven years of dredging?. Environ. Claims J..

[13] Rice C. P., White D. S. (1987). PCB availability
assessment of river dredging using
caged clams and fish. Environ. Toxicol. Chem..

[14] Abel S., Akkanen J. (2019). Novel, activated carbon-based material for in-situ
remediation of contaminated sediments. Environ.
Sci. Technol..

[15] Bako C. M., Martinez A., Ewald J. M., Hua J. B. X., Ramotowski D. J., Dong Q., Schnoor J. L., Mattes T. E. (2022). Aerobic bioaugmentation
to decrease polychlorinated biphenyl (PCB) emissions from contaminated
sediments to air. Environ. Sci. Technol..

[16] Bako C. M., Mattes T. E., Marek R. F., Hornbuckle K. C., Schnoor J. L. (2021). Biodegradation of PCB congeners by *Paraburkholderia
xenovorans* LB400 in presence and absence of sediment
during lab bioreactor experiments. Environ.
Pollut..

[17] Tehrani R., Lyv M. M., Kaveh R., Schnoor J. L., Van Aken B. (2012). Biodegradation
of mono-hydroxylated PCBs by Burkholderia xenovorans. Biotechnol. Lett..

[18] Needham T. P., Payne R. B., Sowers K. R., Ghosh U. (2019). Kinetics of
PCB Microbial
Dechlorination Explained by Freely Dissolved Concentration in Sediment
Microcosms. Environ. Sci. Technol..

[19] Field J. A., Sierra-Alvarez R. (2008). Microbial transformation and degradation of polychlorinated
biphenyls. Environ. Pollut..

[20] Denef V. J., Patrauchan M. A., Florizone C., Park J., Tsoi T. V., Verstraete W., Tiedje J. M., Eltis L. D. (2005). Growth Substrate-
and Phase-Specific Expression of Biphenyl, Benzoate, and C1Metabolic
Pathways in *Burkholderia xenovorans* LB400. J. Bacteriol..

[21] Perelo L. W. (2010). In situ
and bioremediation of organic pollutants in aquatic sediments. J. Hazard. Mater..

[22] Capozzi S. L., Bodenreider C., Prieto A., Payne R. B., Sowers K. R., Kjellerup B. V. (2019). Colonization
and growth of dehalorespiring biofilms
on carbonaceous sorptive amendments. Biofouling.

[23] Borja J. Q., Auresenia J. L., Gallardo S. M. (2006). Biodegradation of polychlorinated
biphenyls using biofilm grown with biphenyl as carbon source in fluidized
bed reactor. Chemosphere.

[24] Zhao L., Xiao D., Liu Y., Xu H., Nan H., Li D., Kan Y., Cao X. (2020). Biochar as
simultaneous shelter,
adsorbent, pH buffer, and substrate of *Pseudomonas
citronellolis* to promote biodegradation of high concentrations
of phenol in wastewater. Water Res..

[25] Liu Y., Chen H., Zhao L., Li Z., Yi X., Guo T., Cao X. (2021). Enhanced trichloroethylene
biodegradation: Roles of
biochar-microbial collaboration beyond adsorption. Sci. Total Environ..

[26] Pignatello J. J., Mitch W. A., Xu W. (2017). Activity and
reactivity of pyrogenic
carbonaceous matter toward organic compounds. Environ. Sci. Technol..

[27] Lian F., Xing B. (2017). Black carbon (biochar)
in water/soil environments: molecular structure,
sorption, stability, and potential risk. Environ.
Sci. Technol..

[28] Uchimiya M., Wartelle L. H., Klasson K. T., Fortier C. A., Lima I. M. (2011). Influence
of pyrolysis temperature on biochar property and function as a heavy
metal sorbent in soil. J. Agric. Food Chem..

[29] Yu L., Yuan Y., Tang J., Wang Y., Zhou S. (2015). Biochar as
an electron shuttle for reductive dechlorination of pentachlorophenol
by *Geobacter sulfurreducens*. Sci. Rep..

[30] Lou L., Huang Q., Lou Y., Lu J., Hu B., Lin Q. (2019). Adsorption and degradation in the
removal of nonylphenol from water
by cells immobilized on biochar. Chemosphere.

[31] Chen Y., Yu B., Lin J., Naidu R., Chen Z. (2016). Simultaneous adsorption
and biodegradation (SAB) of diesel oil using immobilized *Acinetobacter venetianus* on porous material. Chem. Eng. J..

[32] Wu B., Wang Z., Zhao Y., Gu Y., Wang Y., Yu J., Xu H. (2019). The performance of biochar-microbe multiple biochemical
material on bioremediation and soil micro-ecology in the cadmium aged
soil. Sci. Total Environ..

[33] McDonough K. M., Fairey J. L., Lowry G. V. (2008). Adsorption
of polychlorinated biphenyls
to activated carbon: Equilibrium isotherms and a preliminary assessment
of the effect of dissolved organic matter and biofilm loadings. Water Res..

[34] Cho Y.-M., Werner D., Choi Y., Luthy R. G. (2012). Long-term
monitoring
and modeling of the mass transfer of polychlorinated biphenyls in
sediment following pilot-scale in-situ amendment with activated carbon. J. Contam. Hydrol..

[35] Kirtay V., Conder J., Rosen G., Magar V., Grover M., Arblaster J., Fetters K., Chadwick B. (2018). Performance
of an in
situ activated carbon treatment to reduce PCB availability in an active
harbor. Environ. Toxicol. Chem..

[36] Dong Q., LeFevre G. H., Mattes T. E. (2024). Black Carbon
Impacts on *Paraburkholderia xenovorans* Strain LB400 Cell Enrichment
and Activity: Implications toward Lower-Chlorinated Polychlorinated
Biphenyls Biodegradation Potential. Environ.
Sci. Technol..

[37] Payne R. B., Ghosh U., May H. D., Marshall C. W., Sowers K. R. (2017). Mesocosm
studies on the efficacy of bioamended activated carbon for treating
PCB-impacted sediment. Environ. Sci. Technol..

[38] Payne R. B., Ghosh U., May H. D., Marshall C. W., Sowers K. R. (2019). A pilot-scale
field study: in situ treatment of PCB-impacted sediments with bioamended
activated carbon. Environ. Sci. Technol..

[39] Payne R. B., Fagervold S. K., May H. D., Sowers K. R. (2013). Remediation of polychlorinated
biphenyl impacted sediment by concurrent bioaugmentation with anaerobic
halorespiring and aerobic degrading bacteria. Environ. Sci. Technol..

[40] Mukerjee-Dhar G., Shimura M., Kimbara K. (1998). Degradation
of polychlorinated biphenyl
by cells of *Rhodococcus opacus* strain TSP203 immobilized
in alginate and in solution. Enzyme Microb.
Technol..

[41] Nassif N., Bouvet O., Rager M. N., Roux C., Coradin T., Livage J. (2002). Living bacteria in silica gels. Nat. Mater..

[42] Tuttolomondo M. V., Alvarez G. S., Desimone M. F., Diaz L. E. (2014). Removal of azo dyes
from water by sol–gel immobilized *Pseudomonas* sp. J. Environ. Chem. Eng..

[43] Dong Q., Mattes T. E., LeFevre G. H. (2025). Development
of a Novel PCB-Degrading
Biofilm Enriched Biochar Encapsulated with Sol–Gel: A Protective
Layer to Sustain Biodegradation Activity. ACS
EST Eng..

[44] Battelle Memorial Institute . Water Quality Monitoring Summary Report. https://semspub.epa.gov/work/01/580306.pdf (accessed May 19, 2022).

[45] World Sea Temperature . New Bedford Water Temperature. https://www.seatemperature.org/north-america/united-states/new-bedford.htm (accessed May 19, 2022).

[46] Karanfil T., Kitis M., Kilduff J. E., Wigton A. (1999). Role of Granular Activated
Carbon Surface Chemistry on the Adsorption of Organic Compounds. 2.
Natural Organic Matter. Environ. Sci. Technol..

[47] Li, X. ; Dong, Q. ; LeFevre, G. H. ; Mattes, T. E. ; Lehmler, H.-J. Characterization and Authentication 2,2′,5-Trichlorobiphenyl (PCB18); University of Iowa, 2025.

[48] Li, X. ; Dong, Q. ; LeFevre, G. H. ; Mattes, T. E. ; Lehmler, H.-J. Characterization and Authentication 2,2′-Dichlorobiphenyl (PCB4); University of Iowa, 2025.

[49] Li X., Holland E. B., Feng W., Zheng J., Dong Y., Pessah I. N., Duffel M. W., Robertson L. W., Lehmler H.-J. (2018). Authentication of synthetic environmental contaminants
and their (bio)­transformation products in toxicology: polychlorinated
biphenyls as an example. Environ. Sci. Poll.
Res..

[50] Sethi S., Morgan R. K., Feng W., Lin Y., Li X., Luna C., Koch M., Bansal R., Duffel M. W., Puschner B., Zoeller R. T., Lehmler H.-J., Pessah I. N., Lein P. J. (2019). Comparative Analyses of the 12 Most Abundant PCB Congeners
Detected in Human Maternal Serum for Activity at the Thyroid Hormone
Receptor and Ryanodine Receptor. Environ. Sci.
Technol..

[51] Martinez A., Wang K., Hornbuckle K. C. (2010). Fate of PCB Congeners in an Industrial
Harbor of Lake Michigan. Environ. Sci. Technol..

[52] Wang H., Adamcakova-Dodd A., Lehmler H.-J., Hornbuckle K. C., Thorne P. S. (2022). Toxicity assessment
of 91-day repeated inhalation exposure
to an indoor school air mixture of PCBs. Environ.
Sci. Technol..

[53] Sharma, T. Biochar and other properties resulting from the gasification and combustion of biomass with different components; University of Iowa: Iowa City, IA, 2019.

[54] Ciriminna R., Fidalgo A., Ilharco L. M., Pagliaro M. (2015). Sol-Gel Microspheres
Doped with Glycerol: A Structural Insight in Light of Forthcoming
Applications in the Polyurethane Foam Industry. ChemistryOpen.

[55] Conroy J. F. T., Power M. E., Martin J., Earp B., Hosticka B., Daitch C. E., Norris P. M. (2000). Cells in Sol-Gels I: A Cytocompatible
Route for the Production of Macroporous Silica Gels. J. Sol–Gel Sci. Technol..

[56] Billingsley, K. A. ; Juneson, C. ; Ward, O. P. ; Backus, S. M. Comparison of the degradation patterns of polychlorinated biphenyl congeners in Aroclors by Pseudomonas strain LB400 after growth on various carbon sources Can. J. Microbiol., 43 12 1172 1179 10.1139/m97-166. 9537788

[57] Kawai, F. Biodegradation of Polyethers (Polyethylene Glycol, Polypropylene Glycol, Polytetramethylene glycol, and Others). In Biopolymers; Wiley.

[58] Richards P. M., Liang Y., Johnson R. L., Mattes T. E. (2019). Cryogenic soil coring
reveals coexistence of aerobic and anaerobic vinyl chloride degrading
bacteria in a chlorinated ethene contaminated aquifer. Water Res..

[59] Johnson D. R., Lee P. K., Holmes V. F., Alvarez-Cohen L. (2005). An internal
reference technique for accurately quantifying specific mRNAs by real-time
PCR with application to the tceA reductive dehalogenase gene. Appl. Environ. Microbiol..

[60] Liang Y., Meggo R., Hu D., Schnoor J. L., Mattes T. E. (2014). Enhanced
polychlorinated biphenyl removal in a switchgrass rhizosphere by bioaugmentation
with *Burkholderia xenovorans* LB400. Ecol. Eng..

[61] Petrić I., Hršak D., Fingler S., Udiković-Kolić N., Bru D., Martin-Laurent F. (2011). Insight in the PCB-degrading functional
community in long-term contaminated soil under bioremediation. J. Soils Sediments.

[62] Bustin S. A., Benes V., Garson J. A., Hellemans J., Huggett J., Kubista M., Mueller R., Nolan T., Pfaffl M. W., Shipley G. L., Vandesompele J., Wittwer C. T. (2009). The MIQE guidelines: minimum information for publication
of quantitative real-time PCR experiments. Clin.
Chem..

[63] Martinez A., O’Sullivan C., Reible D., Hornbuckle K. C. (2013). Sediment
pore water distribution coefficients of PCB congeners in enriched
black carbon sediment. Environ. Pollut..

[64] Ramotowski D. J., Martinez A., Marek R. F., Hornbuckle K. C., Mattes T. E. (2025). Paraburkholderia Xenovorans Strain
LB400 Significantly
Decreased Volatilization of Polychlorinated Biphenyls (PCBs) from
Freshwater and Saline Sediments. ACS EST Water.

[65] Song Q., Kong F., Liu B.-F., Song X., Ren H.-Y. (2024). Biochar-based
composites for removing chlorinated organic pollutants: Applications,
mechanisms, and perspectives. Environ. Sci.
Ecotechnol..

[66] Jonker M. T. O., Koelmans A. A. (2002). Sorption of Polycyclic Aromatic Hydrocarbons and Polychlorinated
Biphenyls to Soot and Soot-like Materials in the Aqueous Environment:
Mechanistic Considerations. Environ. Sci. Technol..

[67] Braz Á.
G., Pinto J. V., Pulcinelli S. H., Santilli C. V. (2024). The role of PEG/glycerol
ratio on the structure and corrosion protection properties of PU-silica
coating. Surf. Coat. Technol..

[68] Deshpande A. V., Rane J. R., Jathar L. V. (2009). Effect
of glycerol on the optical
and physical properties of sol–gel glass matrices. J. Sol–Gel Sci. Technol..

[69] Raghunandan K., McHunu S., Kumar A., Kumar K. S., Govender A., Permaul K., Singh S. (2014). Biodegradation
of glycerol using
bacterial isolates from soil under aerobic conditions. J. Environ. Sci. Health, Part A: Toxic/Hazard. Subst. Environ.
Eng..

[70] Watson G. K., Jones N. (1977). The biodegradation of polyethylene
glycols by sewage bacteria. Water Res..

[71] Ruan Y., Kuzyakov Y., Liu X., Zhang X., Xu Q., Guo J., Guo S., Shen Q., Yang Y., Ling N. (2023). Elevated temperature
and CO2 strongly affect the growth strategies of soil bacteria. Nat. Commun..

[72] Alvarez G. S., Desimone M. F., Diaz L. E. (2007). Immobilization of bacteria in silica
matrices using citric acid in the sol–gel process. Appl. Microbiol. Biotechnol..

[73] Desimone M. F., De Marzi M. C., Copello G. J., Fernández M. M., Malchiodi E. L., Diaz L. E. (2005). Efficient preservation
in a silicon
oxide matrix of *Escherichia coli*, producer
of recombinant proteins. Appl. Microbiol. Biotechnol..

[74] Zhang X., Wang H., Li Q., Yin Z., Qi H., Yang J., Wang X., Xiao W., Zhang L. (2022). Development
of Organogels for Live *Yarrowia lipolytica* Encapsulation. J. Am. Chem. Soc..

[75] Hoyos-Leyva J. D., Bello-Pérez L. A., Agama-Acevedo E., Alvarez-Ramirez J. (2018). Thermodynamic analysis for assessing
the physical stability
of core materials microencapsulated in taro starch spherical aggregates. Carbohydr. Polym..

[76] Egorova D. O., Pervova M., Demakov V., Plotnikova E. (2018). Specific features
of chlorinated biphenyl decomposition by *Rhodococcus
wratislaviensis* strain KT112–7 under high salt
conditions. Appl. Biochem. Microbiol..

[77] Ganesh-Kumar S., Kalimuthu K., Jebakumar S. R. (2013). A novel bacterium that degrades Aroclor-1254
and its *bphC* gene encodes an extradiol aromatic ring
cleavage dioxygenase (EARCD). Water Air Soil
Poll..

[78] Molloy P. J., Smith M. J., Cowling M. J. (2000). The effects
of salinity and temperature
on the behaviour of polyacrylamide gels. Mater.
Des..

[79] Rasmussen M. T., Saito A. M., Hyman M. R., Semprini L. (2020). Co-encapsulation
of
slow release compounds and *Rhodococcus rhodochrous* ATCC 21198 in gellan gum beads to promote the long-term aerobic
cometabolic transformation of 1,1,1-trichloroethane, cis-1,2-dichloroethene
and 1,4-dioxane. Environ. Sci. Proc. Impacts.

[80] Cudowski A., Pietryczuk A., Górniak A. (2022). Effect of Humic Acid on the Growth
and Metabolism of Candida albicans Isolated from Surface Waters in
North-Eastern Poland. Int. J. Environ. Res.
Public Health.

[81] Venezia V., Avallone P. R., Vitiello G., Silvestri B., Grizzuti N., Pasquino R., Luciani G. (2022). Adding Humic
Acids
to Gelatin Hydrogels: A Way to Tune Gelation. Biomacromolecules.

[82] Dong, Q. ; Martinez, A. ; Marek, R. F. ; Hornbuckle, K. C. ; LeFevre, G. H. ; Mattes, T. E. Dataset Describing Polychlorinated Biphenyl (PCB) Congener Biodegradation and Biphenyl Dioxygenase Gene (bphA) Expression Levels by Sol-Gel-Coated and Uncoated Biofilms on Biochar under Environmentally Relevant Conditions; Iowa Research Online (IRO), University of Iowa, 2026. 10.2580/data.008184. https://iro.uiowa.edu/esploro/outputs/dataset/9985136443702771.

