# Supplementary material for: Sol–Gel-Encapsulated Biofilms on Biochar Effectively Degrade PCBs and Extend Biodegradation Activity under Environmentally Relevant Conditions
Source: Environ Sci Technol. 2026 Sep 25;60(40):28886–98. doi: 10.1021/acs.est.6c02869 (PMC13674062; doi:10.1021/acs.est.6c02869)
Supplement: Supplementary file 1 [file es6c02869_si_001.pdf]

Supporting Information

Sol-Gel Encapsulated Biofilms on Biochar Effectively Degrade PCBs and Extend Biodegradation Activity under Environmentally Relevant Conditions

Qin Dong,[†,‡] *Weilun Zhao,*[†,‡] *Andres Martinez,*[†,‡] *Rachel F. Marek*,[†,‡] *Keri C. Hornbuckle*,[†,‡]

Xueshu Li,[††] *Hans-Joachim Lehmler,*[††] *Gregory H. LeFevre*[†,‡,*] and *Timothy E. Mattes*[†,‡,*]

[†] Department of Civil and Environmental Engineering, University of Iowa, 4105 Seamans Center, Iowa City, Iowa, 52242, United States

[‡] IIHR—Hydroscience and Engineering, University of Iowa, 100 C. Maxwell Stanley Hydraulics Laboratory, Iowa City, Iowa, 52242, United States

[††] Department of Occupational and Environmental Health, University of Iowa, Iowa City, IA 52242, USA

[*]**Corresponding Authors**: GHL: gregory-lefevre@uiowa.edu; Phone: +319 335 5655, Department of Civil and Environmental Engineering, 4106 Seamans Center for Engineering, University of Iowa, Iowa City IA, 52242, United States. TEM: tim-mattes@uiowa.edu; Phone: +319 335 5065, Department of Civil and Environmental Engineering, 4105 Seamans Center for Engineering, University of Iowa, Iowa City IA, 52242, United States

This supplemental information document includes:

Supplementary methods (Section S1.1-S1.9), Supplementary results (Section S2.1-S2.6), Figures S1-S24, Tables S1-S11. Total Pages (inclusive of this page) is 65.

S1. Supplemental Methods and Materials

S1.1. PCB Master Mix Preparation

Biphenyl (99.5+%) and sodium benzoate (98.0+%) were purchased from TCI America (Portland, OR) and used as received. Dissolved PCB master mixes were made in three concentrations (0.2 μg/L, 1.8 μg/L, and 5 μg/L for each congener), by spiking 0.09 g/L PCB mixtures (PCBs 4, 18, and 52) into a media bottle (depending on the total volume needed) and adding various solvents (DI water, K1 media, saline water, or humic acid solution), followed by equilibrating for 1-day on a platform shaker at 150 rpm. The three PCB concentrations are <20% of the water solubility of each PCB congener and thus ensure the dissolution of PCBs in water.

S1.2. SPME-Water Partition Coefficient (K_{PDMS-w})

S1.2.1 Experimental Determination of SPME-Water Equilibrium Partition Coefficients under Different Environmental Conditions

Passive sampling has been widely used to determine freely dissolved aqueous concentrations of hydrophobic organic compounds (HOC) (e.g., PCBs) in surface water and porewater of sediments and soils,[1,2] using different polymer sampling materials to accumulate target chemicals by equilibrium partitioning.[3] The partition coefficient between water and polymers was determined under different environmental conditions because temperature and salinity could affect the partitioning of chemicals to polymers.[4]

Passive PCB sampling was performed by using solid phase microextraction (SPME) fibers,[5-9] with a 210 µm inner diameter and a 10 µm-thick layer of Polydimethylsiloxane (PDMS) coating. SPME fibers were cleaned by immersing in hexane for 24 hours. The fibers were then removed from hexane, wrapped in clean aluminum foil, and stored in -20°C until ready for use. Equilibrium partition coefficients of SPME-water (hereafter referred to as K_{PDMS-w}) were measured in a 250-mL ASE collection bottle with Teflon lined caps and filled with 250 mL of PCB master mixes (PCBs 4, 18, and 52; n=3),[10-12] in which three different initial PCB concentrations as described in S1.1 (0.2 µg/L, 1.8 µg/L, and 5 µg/L) were tested. PCB solutions were combined with DI water and saline water to achieve three different salinities: 0 g/L, 20 g/L, and 30 g/L. Bottles containing freshwater (0 g/L salinity) were incubated at 5°C, 10°C, and room temperature (20-21°C), with shaking, to evaluate the impacts of temperature on K_{PDMS-w}. Bottles containing water with 20 g/L and 30 g/L salinities were shaken at room temperature (20-21°C) to test the impacts of salinities on K_{PDMS-w}.

To ensure negligible depletion during SPME sampling and to avoid disturbing PCB equilibrium (i.e., the PCB fraction sorbed to the fiber was <5% of the total PCB mass), approximately 2 cm of SPME fiber was deployed per bottle. At equilibrium, the mass of PCBs absorbed into the PDMS coating is governed by the PDMS volume, the fiber–water partition coefficient, the total water volume, and the presence of sorbents. Theoretical calculations were performed to determine the equilibrium distribution of PCBs among the fiber, aqueous, and sorbent-associated phases as a function of PDMS volume. Fiber length was adjusted to ensure that the sorbed mass did not exceed 5% of the freely dissolved PCB fraction, maintaining negligible depletion conditions. This approach follows established methods reported by other studies.[10,13-15]. To calculate the freely dissolved PCB concentrations from the PCBs that accumulated in the SPME fiber, PCBs absorbed in the fibers were desorbed with 1 day of hexane extraction. Remaining PCBs in PDMS after solvent extraction is negligible because the hexane volume (~1 mL) is in large excess relative to the PDMS (~6.9×10^{-5} mL per cm of SPME). This condition strongly favors complete PCB transfer from the fiber into the hexane phase.[16-18] Liquid samples (2 mL per bottle) were collected by analytical syringe for measuring the water concentration, placed in Pyrex test tubes, and stored at -20°C until PCB extraction and analysis. SPME fibers were retrieved by pouring the PCB solution through the steel mesh (No. 50, 450°C oven heated). PCB sorption to Pyrex test tubes and steel mesh was considered negligible according to our previously validated analytical methods.[19-23] In addition, because the bioreactor controls and treatments were handled identically, this would cancel out any minor PCB sorption effects associated with the glassware or steel mesh. SPME fibers were rinsed with Optima water, wiped with a Kimwipe, measured with a ruler, and placed in a glass gas chromatography (GC) vial insert. The GC vial with an insert was

spiked with internal standard (S1.7) and filled with hexane to facilitate PCB desorption from fibers, which desorption lasted for at least one day at -20°C.

S1.2.2 Calculate Equilibrium SPME-Water ($K_{PDMS\text{-}w}$*) Partition Coefficients*

Experimental partition coefficients of PDMS-water ($K_{PDMS\text{-}w}$) were obtained by dividing the average concentration in the PDMS passive samplers by the average concentration in the water phases (Equation S1). The passive sampler concentrations were calculated from the masses in the sampler and the length of the sampler. The water phase concentrations were calculated from the recovery-corrected masses in the water phase extracts and the volume of the PCB solution. PDMS volume was 6.9×10^{-8} L/cm-fiber. Experimental $K_{PDMS\text{-}w}$ values for each PCB congener (PCBs 4, 18, and 52) were calculated by averaging the $K_{PDMS\text{-}w}$ determined from three PCB concentrations (0.2 µg/L, 1.8 µg/L, and 5 µg/L). This approach is appropriate because, due to the partitioning process between the SPME fiber and the water phase, the water concentration does not influence the partitioning coefficient ($K_{PDMS\text{-}w}$). The use of three concentrations served to confirm that comparable $K_{PDMS\text{-}w}$ values were obtained across the tested range and to evaluate the variability and reproducibility of the method. Water phase extraction recoveries were 101% ± 6%.

Equation S1:

$$K_{\mathrm{PDMS-w}} = \frac{\frac{m_{SPME}}{V_{SPME}}}{C_{water}} = \frac{m_{SPME}}{C_{water} \times V_{SPME}} = \frac{C_{SPME}}{C_{water}}$$

S1.2.3 SPME-Water Partition Coefficient ($K_{PDMS\text{-}w}$*) - Results*

This study determined PDMS-water partition coefficients ($K_{PDMS\text{-}w}$) for a range of concentrations of the selected LC-PCB congeners (PCB4, PCB18, and PCB52) under standard

conditions (e.g., fresh water and room temperature). Based on our PCB performance test, we prepared three concentrations of PCB solution, ranging from 200 ng/L to 5 µg/L. Figure S1 demonstrates the linear relationship between $K_{PDMS\text{-}w}$ and K_{ow} of three PCB congeners with low variability ($r^2 = 0.90$), which complies to one of the criteria for best partition coefficients.[3] Additionally, all three PCB concentrations well aligned with the linearity and $K_{PDMS\text{-}w}$ from other studies.[3] We proposed our own $K_{PDMS\text{-}w}$ values (log_{10} scale) for three PCB congeners under standard conditions by averaging $K_{PDMS\text{-}w}$ values from different PCB concentration experimental tests, and they were 4.25 for PCB4, 4.99 for PCB18, and 5.29 for PCB 52.

We also determined $K_{PDMS\text{-}w}$ for PCB4, PCB18, and PCB52 at different temperatures and salinities to improve the accuracy of estimating aqueous PCB concentrations under environmentally relevant conditions. Temperature positively affected $K_{PDMS\text{-}w}$, especially PCB52 had significantly larger $K_{PDMS\text{-}w}$ at high temperature (21°C) compared to low temperature (5°C)($p=0.044$). PCBs with more chlorines had larger increases in $K_{PDMS\text{-}w}$ (PCB4: 0.1, PCB18: 0.54, PCB52: 1.2 log increase; Figure S2). Salinity impacts on $K_{PDMS\text{-}w}$ were less consistent (PCB4 $p>0.05$, PCB18&52 $p=0.0002$), where 20 g/L had the lowest $K_{PDMS\text{-}w}$ among selected salinities (0 g/L vs 20 g/L, 20 g/L vs 30 g/L, $p<0.0009$ for PCB18 and PCB52). Our findings comply to other studies that temperature had larger impacts on $K_{PDMS\text{-}w}$ than salinity,[4] but the trend of impacts was different depending on passive sampler materials.

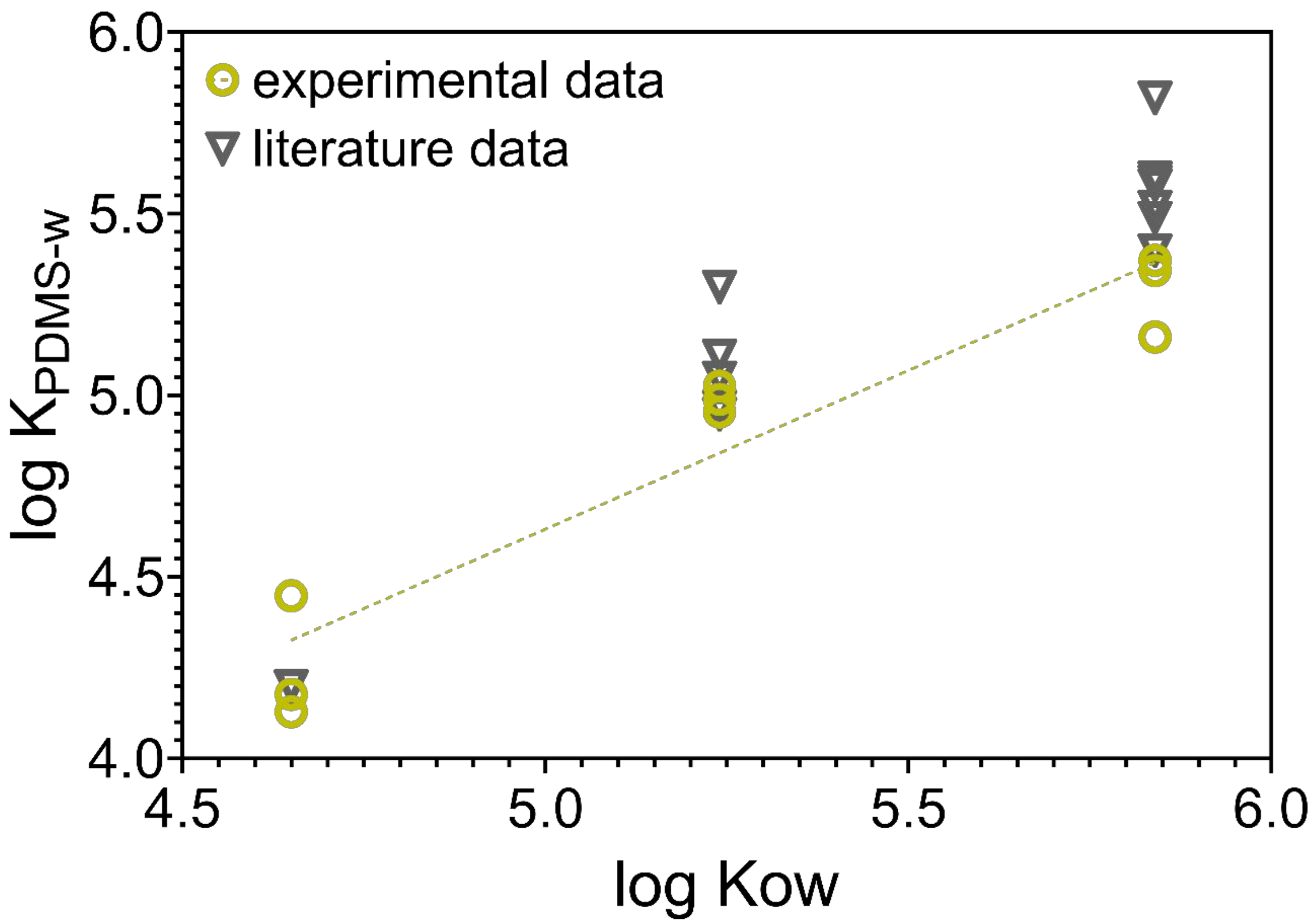

Figure S1. Correlation between log(K_{ow}) and log(K_{PDMS-w}) of three PCB congener mixtures. Green circles represent experimental results in our study, and grey triangles represent (averaged) literature values.[3] Each green circle was averaged by triplicates. Dashed line represents regression line of our experimental data.

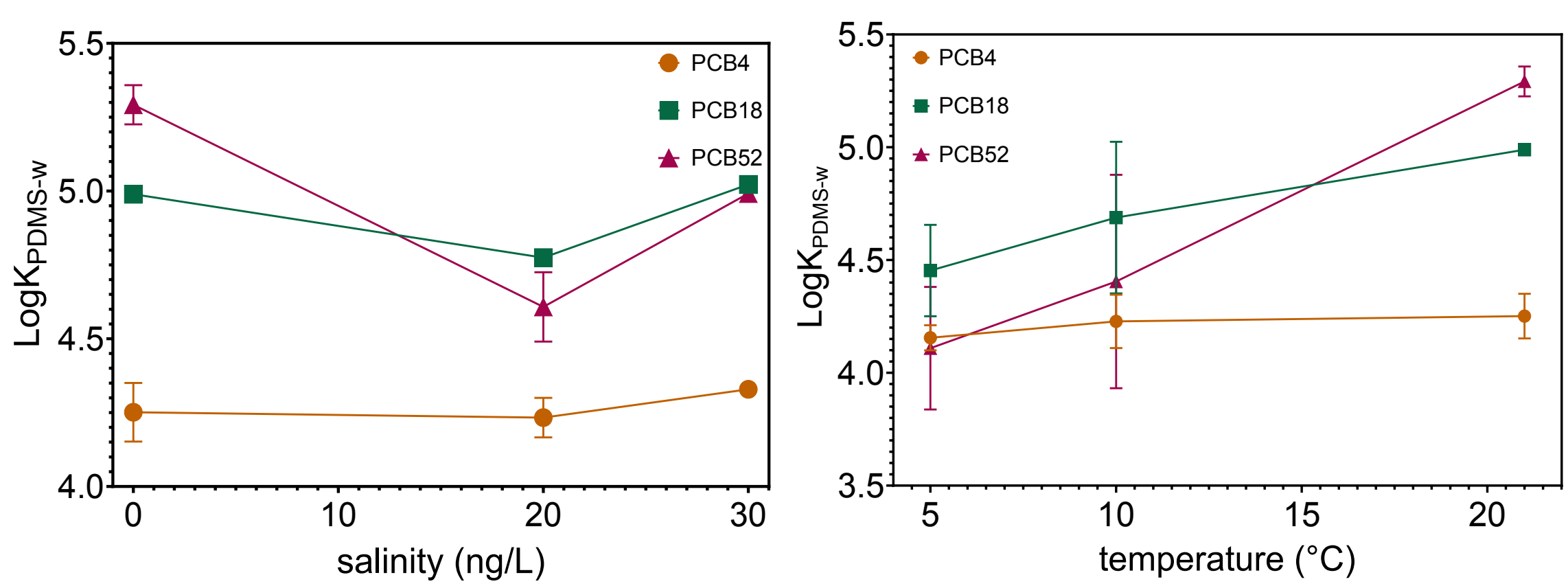

Figure S2. $K_{PDMS-w}$of selected PCB congeners at different salinities and temperatures. Salinities range from 0 g/L (fresh water), 20 g/L, to 30 g/L. Temperatures range from 5°C, 10°C, to 21°C

(room temperature). Orange circles represent $K_{PDMS\text{-}w}$ of PCB4, green squares represent $K_{PDMS\text{-}w}$ of PCB18, and red triangles represent $K_{PDMS\text{-}w}$ of PCB 52. Error bars represent standard error of biological replicates (n=3), except 10°C (n=2) and 20g/L (n=5).

S1.2.4 SPME Fiber Application to Measure Aqueous Concentration in Black Carbon Addition

Many applications of passive samplers were used on measuring bioavailability of hydrophobic organic compounds in contaminated sediment with BC addition,[24,25] but no study was found to apply passive samplers in aqueous solution with only BC. Our study is the first to evaluate the feasibility of passive samplers to measure freely dissolved PCB concentrations in the presence of corn kernel biochar (CKB). To verify $K_{PDMS\text{-}w}$ in the presence of CKBs, different CKB masses were added to evaluate potential impacts on PCB sorption on SPME. Triplicate Pyrex test tubes (50-mL) with Teflon-lined caps were filled with 30 mL of 5 µg/L PCB mixtures (PCBs 4, 18, and 52) and 0.42%, 1%, or 1.8% CKB mass loading (0.126 g, 0.3 g, and 0.54 g), and a ~0.5 cm SPME fiber was added to each tube. All test tubes were shaken on platform shaker at 30 rpm for 4-7 days. At the time of sampling, CKB and SPME were retrieved by pouring sample solution through No. 50 steel mesh (450°C oven heated overnight). Fibers were treated as described above, and CKBs were air dried in a fume hood for 1 hour prior to PCB extraction and analysis. The SPME polymer–water partition coefficient is independent of the surrounding sorbent matrices, ensuring that the determined concentrations accurately reflect the freely dissolved fraction, regardless of the extent of PCB sorption to solid phases.[26,27] Although additional sorbents such as sediment organic matter, biochar, or sol–gel modified biochar can reduce the freely dissolved PCB concentration by binding contaminants, they do not interfere with the SPME measurement.

S1.2.5 SPME Fiber Application in BC Addition Results

This study also evaluated the feasibility of our proposed $K_{PDMS\text{-}w}$ values in PCB solution with various BC additions. We added three different BC additions, ranging from 0.42% to 1.8%,

in PCB solution with the same amount of SPME addition to test PCB mass remaining in aqueous phase. For all PCB congeners, more BC mass addition resulted in lower aqueous PCB concentrations (Figure S3), and PCB concentrations and trend comply to those calculated by best available $K_{PDMS\text{-}w}$ values proposed in other studies[3], except 1.6-fold higher PCB 52 concentrations in our experiment. Theoretical concentrations were calculated based on BC sorption of 5 μg/L PCBs, and they were over 2 times larger than SPME measured PCB concentrations potentially because of large variance of PCB dissolution in water, large variance in BC sorption capacity, and minor variances in $K_{PDMS\text{-}w}$, especially at such low PCB concentrations. Our results indicate that our proposed $K_{PDMS\text{-}w}$ values were able to estimate aqueous PCB concentrations in solution with black carbon (CKB) present. This study is the first to demonstrate the feasibility of using polymers (such as SPME) to capture quantifiable contaminants in the aqueous phase with the relatively high sorption capability of BC also present. This is important not only because passive samplers are convenient techniques to quantify pollutants compared with liquid-liquid extraction, cheap, and versatile,[28] but also SPME application could address the challenges of low PCB solubility in water.

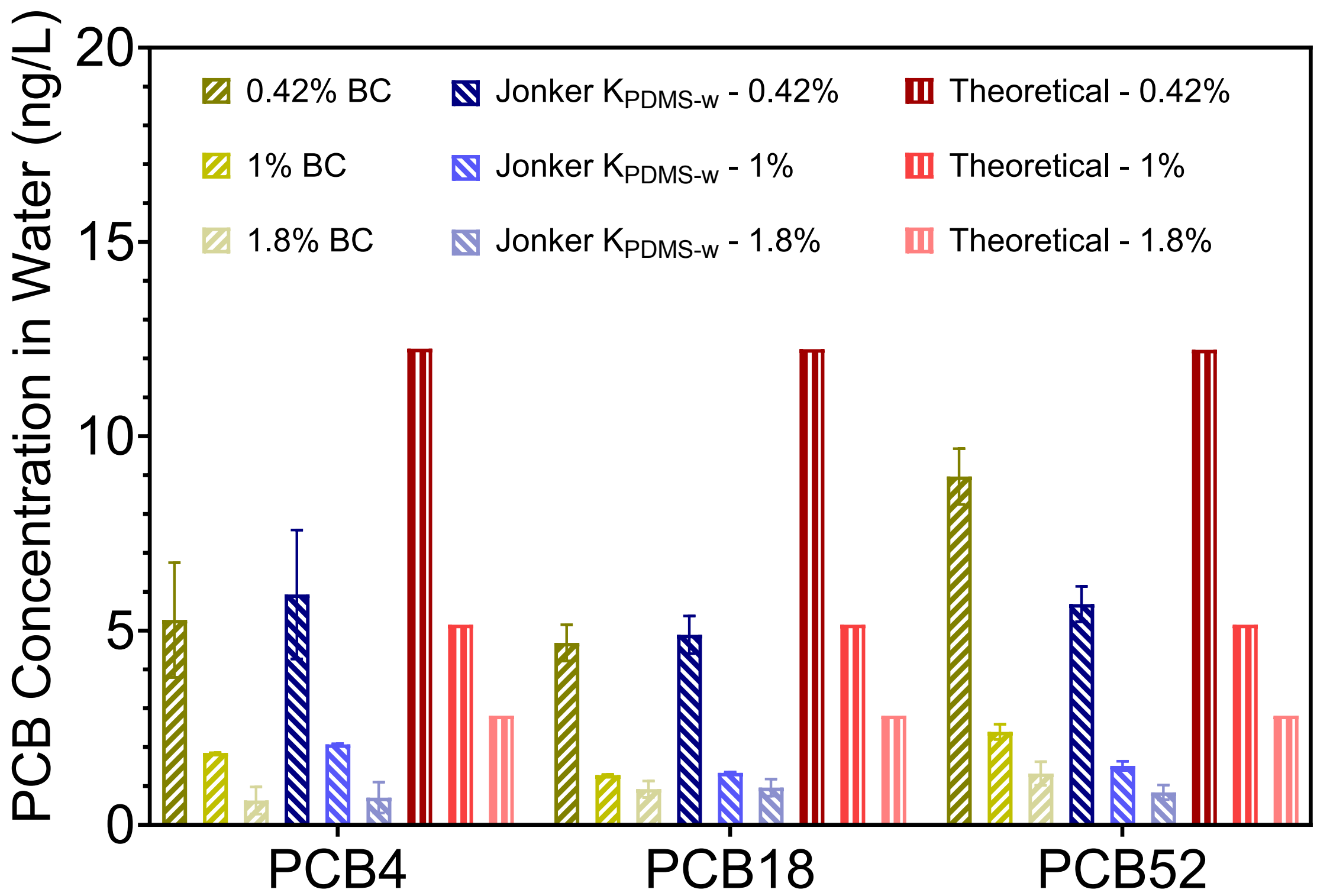

Figure S3. PCB concentrations in water measured by adding SPME fibers to various black carbon mass addition. Testing was conducted in a 50-mL test tube (n=2), filled with 30-mL of 5µg/L PCB solution (PCB4, PCB18, and PCB52), 0.42%, 1%, or 1.8% mass loading of BCs, and a piece of ~0.5cm SPME fiber. Tubes were shaken for one month, and SPME fibers were collected to quantify PCB concentrations. Yellow columns with left diagonal lines represent results calculated with our SPME data and our proposed PCB K_{PDMS-w} values; blue columns with right diagonal lines represent data calculation based on our SPME data and PCB K_{PDMS-w} values proposed in Michiel Jonker 2022[3]; red columns with straight lines represent theoretical values calculated based on BC-water partition coefficient. Error bars represent the standard error of replicate mean (n=2).

S1.3. Partition Coefficient of Sorbent and Water (K_d) for PCBs

Partition Coefficient Measurement. Silica gel is a promising sorbent on various chemicals,[29] and sorbate diffusion is highly affected by the hydrophobicity of both core materials and encapsulants.[30,31] Pure silica gel has been widely used in PCB extraction and cleanup process,[32] but few studies elaborate silica gel sorption potential on PCBs and impacts on PCB mass transfer. Therefore, we evaluated the impacts of sol-gel coating on PCB sorption in this study. Target sorbents included plain sol-gel (made with glycerol or PEG400), plain CKB, killed biofilm-CKB, and sol-gel (sol-gel$_{GLY}$ or sol-gel$_{PEG}$) coated killed biofilm-CKB. K_d was determined in 50-mL Pyrex test tubes containing 30 mL of PCB solution, with initial concentrations of 5 µg/L, 0.42% mass loading (~0.126 g) of target sorbents, and one piece of ~0.5 cm SPME fiber. Sodium azide (48.9 mM) was added to each test tube to inhibit cell contamination and microbial activity. Because sodium azide does not change cell physicochemical properties[33] its use in preparing killed controls should not affect PCB sorption on biofilms. Benzoate degradation was tested to confirm that sodium azide treated biofilms lost microbial activity. Test tubes were mixed on platform shaker at 30 rpm at room temperature (20-21°C) until pseudo-steady state. Based on preliminary experiment results in Figure S4, LC-PCB congeners reached sorption pseudo-equilibrium between sol-gel (glycerol) coated SB and water within 7-16 days, where aqueous concentration of PCBs remained ~100 ng/L at equilibrium. Therefore, 10 days were chosen as the equilibrium time in later experiment to determine sorption potential.

Partition Coefficient Calculation. K_d was calculated based on the initial PCB concentrations and aqueous PCB concentrations at equilibrium. Aqueous PCB concentrations were measured after sorption equilibrium, and sorbed concentrations were calculated through Equation S2. Pseudo-

steady state sorbed concentration (q_e, mg g^{-1}) and aqueous concentration (C_e, mg L^{-1}) were used to evaluate the K_d (Equation S3) of PCB to predict the removal efficiency on sol-gel and biofilms-CKB.

Equation S2

$$q_e = \frac{V}{M}(C_0 - C_e)$$

Equation S3:

$$K_d = \frac{q_e}{C_0}$$

Where q_e refers to pseudo-steady state sorbed concentration (mg g^{-1}), C_0 refers to initial aqueous concentration (mg L^{-1}), C_e refers to pseudo-steady state aqueous concentration (mg L^{-1}), V refers to solution volume (mL), and M refers to the mass of sorbents (g). K_d values are tabulated in Table S5 and also depicted in Figure S10.

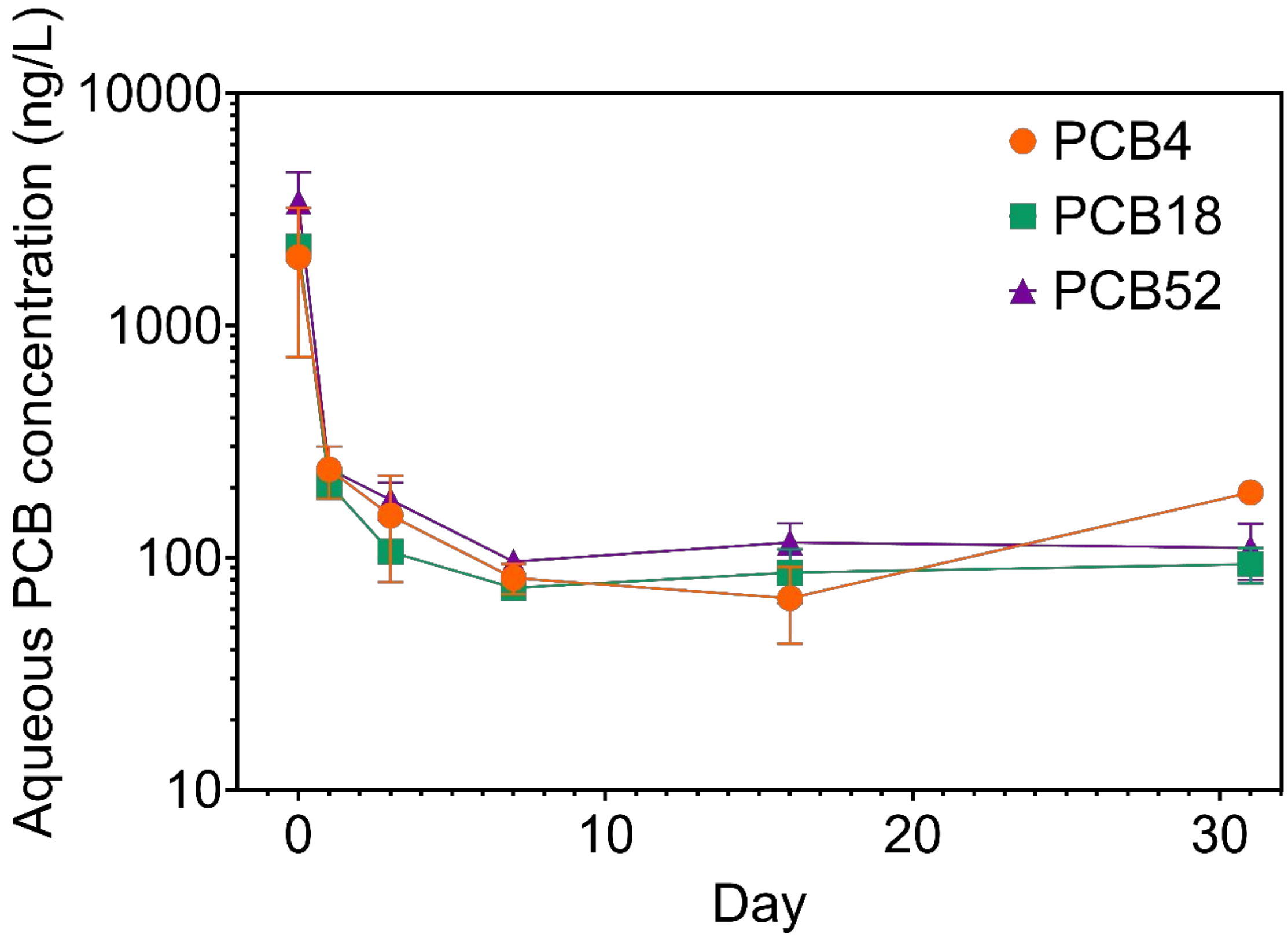

Figure S4. Preliminary experiment testing the time required to reach sorption equilibrium for sol-gel (glycerol)-coated corn kernel biochar (CKB) in PCB mixtures. Orange circles represent aqueous concentrations of PCB4; green squares represent aqueous concentrations of PCB18; purple triangles represent aqueous concentrations of PCB52. (n=2)

S1.4. Biofilm Soaking with Benzoate Prior to Sol-gel Coating

Biofilm Growth. LB400 cells were inoculated into 250mL of sterile K1 medium containing 0.19g of biphenyl crystal until cell cultures reached mid-exponential phase (OD_{600}=0.5-0.6). Washed and concentrated LB400 cells (6.7mL, OD_{600}=1.76) were added to flasks containing K1 medium (60.3mL), biphenyl crystal (50.9mg), and CKB (2g, dry weight), and incubated for 10-days at room temperature (20-21°C) with shaking at 150rpm.

Biofilm Soaking with Benzoate. Around six grams of biofilm-enriched biochar (wet weight) were added to a flask containing 180 mL of K1 medium and 66 mM benzoate, which was then "soaked" with shaking at 150 rpm for 1 hour at room temperature (20-21°C).

S1.5. PCB Biodegradation with Biofilm Attached Biochar and Sol-Gel Coated Biofilms

For appropriate cell conditions during the experiment, liquid K1 medium was used to prepare a master PCB mixture solution (SI 1.1) that was subsequently distributed into each control and treatment test tube.

K1 medium was prepared by adding 100 mL K1 10× stock solution and 20 mL Hutner mix into 880 mL sterile deionized (DI) water, as described in our prior work.[34] K1 10× stock solution was made by adding 15.9 g K_2HPO_4, 2.5 g $(NH_4)_2SO_4$, and 1.77 g $NaH_2PO_4 \cdot H_2O$ into 1000 mL DI water, autoclaving for 30 minutes at 121°C. Sterile vitamins (0.22 µm filtered) were added, including 250 µL 10 mg/mL of thiamine, nicotinic acid, nyo-inositol, and riboflavin in phosphate buffer and 25 µL 2 mg/mL of D-biotin in methanol. Hutner mix contains various salts and "Metals 44" solution. 10 grams of nitrilotriacetic acid (NTA) were added into 600 mL DI water with a magnetic stir bar on a stir plate, followed by KOH pellet addition until the cloudy NTA solution dissolved. Afterwards, 14.5 g $MgSO_4 \cdot 7H_2O$, 3.33 g $CaNO_3$, 9.25 mg $(NH_4)_6Mo_7O_{24} \cdot 24H_2O$, 99 mg $FeSO_4 \cdot 7H_2O$, and 50 mL Metals 44 were added sequentially. After all chemicals were completely mixed, an additional 400 mL DI water was added into the solution, followed by pH adjustment to 6.6-6.8. Solution was filtered through 0.22 µm filters and stored at room temperature (20-21°C). Metals 44 was made by adding the following chemicals to 400 mL DI water: 1 g EDTA, 4.4 g $ZnSO_4 \cdot 7H_2O$, 2 g $FeSO_4 \cdot 7H_2O$, 620 mg $MnSO_4 \cdot H_2O$, 119 mg $CuSO_4 \cdot 5H_2O$, 99.4 mg $Co(NO_3)_2 \cdot 6H_2O$, and 70.8 mg $Na_2B_4O_7 \cdot 10H_2O$. A few drops of H_2SO_4 were added to dissolve metals, and the solution was filtered through 0.22 µm filters and stored at room temperature (20-21°C).

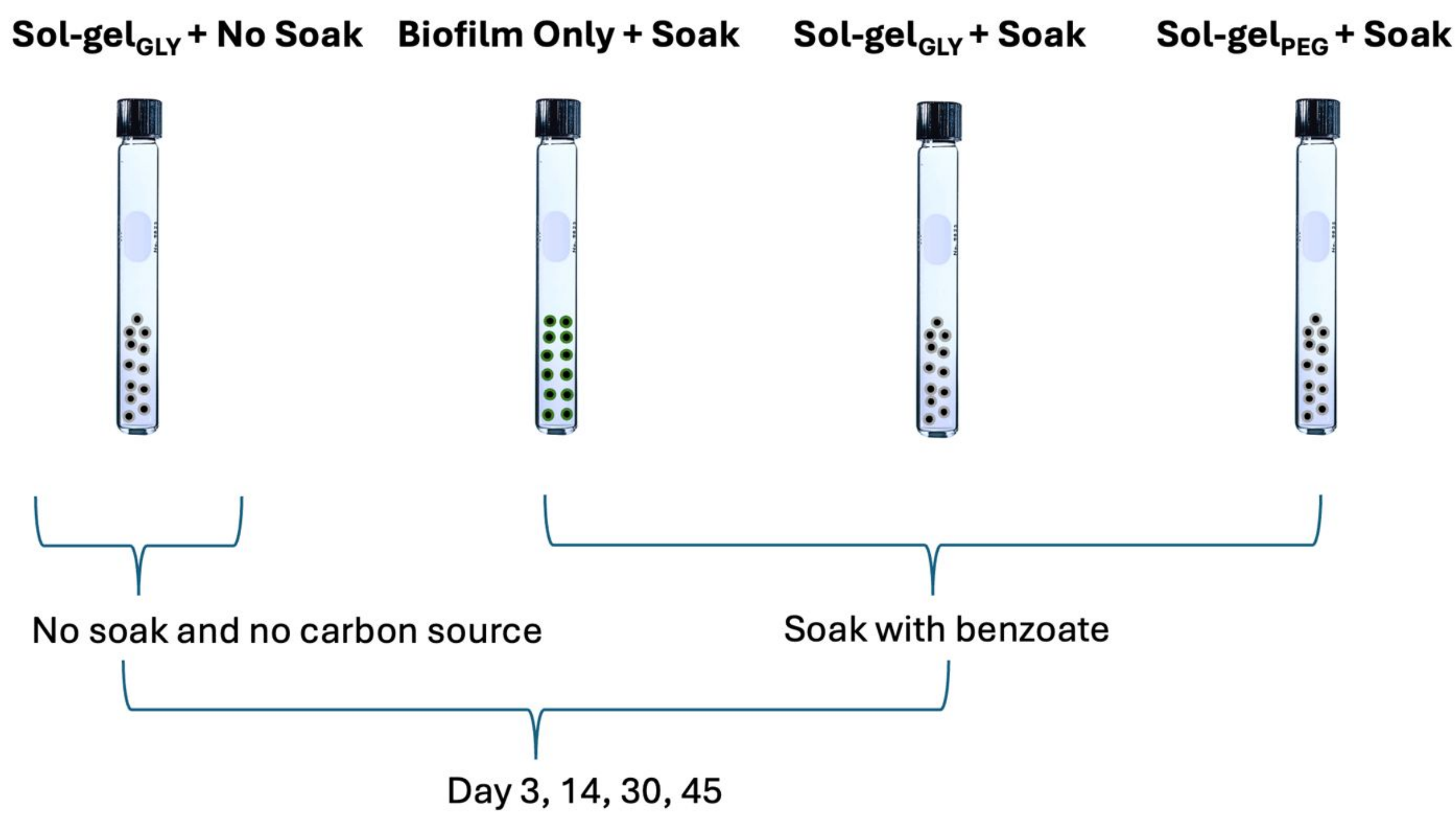

Figure S5. Experimental design of PCB biodegradation under standard conditions. It included four groups (from left to right): Sol-gel$_{GLY}$-coated biofilms without benzoate soaking (Sol-gel$_{GLY}$+No Soak), uncoated biofilms with benzoate soaking (Biofilm Only+Soak), sol-gel$_{GLY}$-coated biofilms with benzoate soaking (Sol-gel$_{GLY}$+Soak), and sol-gel$_{PEG}$-coated biofilms with benzoate soaking (Sol-gel$_{PEG}$+Soak). Each treatment and control group had one extra tube in case of unexpected situations (e.g., contamination).

S1.6. Environmental Condition Impacts on PCB Biodegradation

Oxygen consumption rate. Suspended LB400 cells (24 mL at OD_{600}=0.53) were inoculated in 26 mL of K1 medium containing 0.038 g biphenyl crystal in serum bottles sealed with rubber septa and aluminum crimp caps to measure DO levels over time (n=2). Initial DO level in serum bottles was 1.5%. The half-velocity coefficient for oxygen, $K_s(O_2)$, was determined by fitting the data with a one-phase decay model in Prism (Figure S6).

Dissolved Oxygen (DO) Measurement. DO detection and quantification procedures were performed as previously described.[35] Briefly, headspace samples (200 μL) were collected from sealed serum bottles with a gas-tight syringe and manually injected into an Agilent 6890 gas chromatograph with a thermal conductivity detector (GC-TCD). Oxygen separation was achieved with a Supelco 5A molecular sieve column (5 ft x 1/8 inch, 60/80 mesh) with ultra-high purity helium as the carrier gas (30 ml/min) and an isothermal oven temperature (70°C).

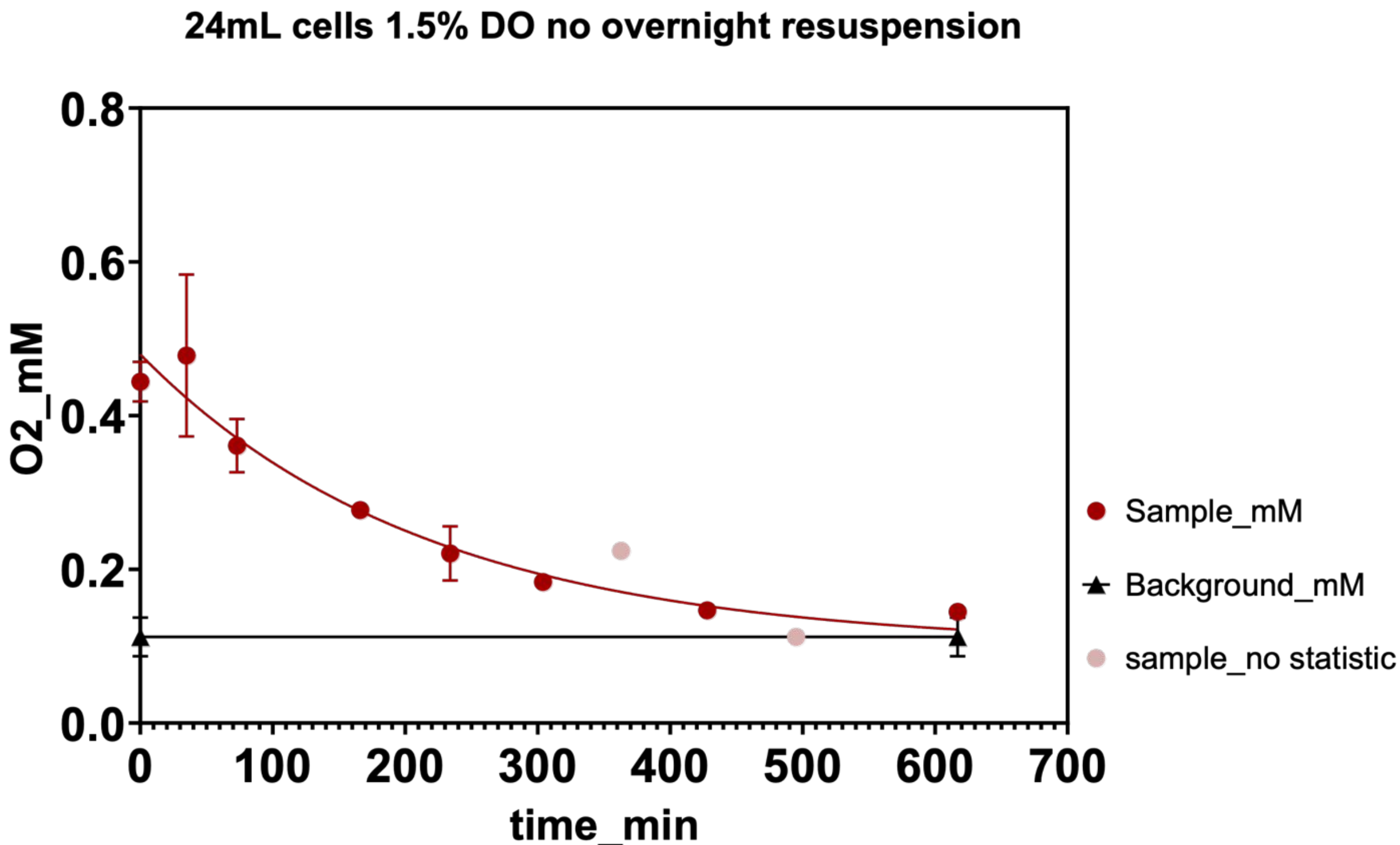

Figure S6. Oxygen consumption rate Ks(O_2). Fitting the data with a one-phase decay model in Prism indicated that the Ks (O_2) = 6 mg/L.

S1.7. PCB Extraction, Quantification, and QA/QC

PCB Extraction. PCBs were extracted from solid samples via pressurized liquid extraction (PLE) with equal parts of acetone and hexane using an accelerated solvent extractor (ASE-350). All materials used in sample extraction had either been triple rinsed with solvents methanol, acetone, and hexane or combusted overnight at 450°C to prevent background PCB contamination. Before PLE, solid samples were retrieved by pouring the PCB solution through the steel mesh (No. 50, 450°C oven heated), followed by air drying the samples for ~1 hr. Dried solid samples were ground with activated diatomaceous earth (DE) in mortar until a free-flowing powder was obtained. ASE cells were loaded with one filter paper on the bottom, ~0.5 g of combusted silica gel, solid samples with DE, and one filter paper on the top, respectively. The ASE cell was spiked with ^{13}C labeled surrogate standards PCB 15 (9.92 ng; 4,4'-dichloro($^{13}C_{12}$)biphenyl), PCB 31 (9.92 ng; 2,4',5-trichloro($^{13}C_{12}$)biphenyl), and PCB 52 (9.92 ng; 2,2',5,5'-tetrachloro($^{13}C_{12}$)biphenyl; Wellington Laboratories). The sample extract resulting from PLE was concentrated within a TurboVap collection tube to ~1 mL using a TurboVap II Concentration Workstation (Biotage, Uppsala, Sweden). The sample extract was then cleaned by sulfuric acid, copper powder, and passing through a silica gel column, respectively. The silica gel column was prepared in a Pasteur pipette filled with glass fiber wool, 0.1 g of silica gel, and 1 g of acidified silica gel (2:1 silica gel/sulfuric acid by weight), respectively. The eluted extract volume was ~10 mL hexane, and it was concentrated again to ~1 mL and transferred to a GC vial. The final extract was spiked with internal standards PCB d30 (10.0 ng; 2,4,6-tri-CB-2′,3′,4′,5′,6′-d5, deuterated) and PCB 204 (10.0 ng; 2,2',3,4,4',5,6,6'-octachlorobiphenyl; New Haven, CT, USA).

PCB Quantification. GC-MS/MS (7890B GC system, 7000D Triple Quad, 7693 autosampler; Agilent, Santa Clara, California) in multiple reaction monitoring mode (MRM) was used for identification and quantification of PCB congeners (PCB4, PCB18, and PCB52) as three chromatographic peaks. The GC was equipped with a Supelco SPB-Octyl capillary column (Poly(50% n-octyl/50% methyl siloxane), 30 m×0.25 mm ID, 0.25 μm film thickness) with ultra-high purity (UHP) helium as carrier gas (0.8 mL/min) and quench gas (2.25 mL/min). UHP nitrogen was used as the collision gas (1.5 mL/min). The GC was equipped with a multi-mode inlet (MMI) which operated in solvent vent injection mode under the following injection conditions: initial temperature 45°C for 0.06 min, ramp 600°C/min to inlet temperature 325°C at 4.4 psi. The GC oven temperature program was 45°C for 2 min, 75°C at 100°C/min and hold for 5 min, 150°C at 15°C/min and hold for 1 min, and 280°C at 2.5°C/min with a final hold for 5 min (total run time 70.86 min). The transfer line temperature was held at 280 °C. The triple quadrupole MS electron ionization source was set to 230°C, the dwell time to 60 ms, and the collision energy to 25 eV.

Quality Assurance and Quality Control. We assessed data quality by considering extraction efficiency, reproducibility, and accuracy. Extraction efficiency was assessed using surrogate standards (^{13}C-labeled PCB 15, PCB 31, and PCB 52). The mean and standard deviation percentage recoveries of surrogate standards in solid samples (sorbents) were 81% ± 11% (PCB 15), 84% ± 8.8% (PCB 31), and 88% ± 8.1% (PCB 52). Percentage recoveries of surrogate standards less than 100% were used to correct PCB congener masses as follows: ^{13}C-PCB 15 recovery was used to correct PCB 4, ^{13}C-PCB 31 recovery was used to correct PCB 18, and ^{13}C-PCB 52 was used to correct PCB 52. Our preliminary data (Table S1) indicated that PCB

volatilization during the 1hr air drying step was minimal, and quantified net PCB mass in aqueous phase and solid phase achieved 100% mass balance without surrogate standard recovery correction. Reproducibility was assessed by setting triplicates of biodegradation tubes/flasks along with laboratory blanks. Laboratory blanks were treated identically to each passive sampler (SPME) or solid sample but were not deployed in the biodegradation tubes/flasks. Mass of SPME fibers were weighed before use, and the length was measured after use. PCB mass detected in SPME blanks and DE blanks was used to determine the limit of quantification (LOQ) as the upper limit of the 95% confidence interval. Congener masses below LOQ are reported as 0. LOQ values of SPME for PCBs 4, 18, and 52 were 0.0027 ng, 0.013 ng, and 0.15 ng, respectively. LOQ values of sorbents for PCBs 4, 18, and 52 were 0.12 ng, 0.37 ng, and 2.69 ng, respectively.

Table S1. PCB mass balance data for air drying step used in the biochar PCB extraction process. Initial validation experiments were performed with two different samples (sol-gel CKB+biofilm and plain sol-gel). Measured initial PCB mass added (actual PCB spike (ng)), measured equilibrium aqueous mass of PCB in water (ng), and measured PCB mass extracted from biochar (PCB by sorption (ng)) is shown. The total PCB mass (water+sorption, ng) is provided for comparison to the actual PCB spike. All values are shown both with or without surrogate standard (SS) correction, because surrogates were added after the air drying step. The recovery % of surrogates is also provided.

Sample		PCB in water (ng)			PCB by sorption (ng)			total PCB (water+sorption, ng)			actual PCB spike (ng)		
	PCB congener	PCB4	PCB18	PCB52	PCB4	PCB18	PCB52	PCB4	PCB18	PCB52	PCB4	PCB18	PCB52
	non-SS corrected	1.48	0.74	1.01	68.14	62.53	69.78	69.62	63.28	70.79	69.39	53.28	60.35
sol-gel CKB+biofilm	SS-corrected	1.48	0.74	1.01	68.14	64.17	74.33	69.62	64.92	75.34	69.39	53.28	60.35
	recovery%				1.00	0.97	0.94						
	PCB congener	PCB4	PCB18	PCB52	PCB4	PCB18	PCB52	PCB4	PCB18	PCB52	PCB4	PCB18	PCB52
	non-SS corrected	10.83	3.68	3.74	59.88	54.53	55.17	70.71	58.21	58.91	69.39	53.28	60.35
plain sol-gel	SS-corrected	10.83	3.68	3.74	65.48	55.06	56.54	76.31	58.74	60.28	69.39	53.28	60.35
	recovery%				0.91	0.99	0.98						

S1.8. Nucleic Acid Extraction, RT-qPCR, and QA/QC

Modified Procedures for RNA extraction. Instead of vortexing the PowerBead Tube at maximum speed for 15 minutes, we increased vortexing time to 25 minutes. Other steps followed the instructions of the RNeasy PowerSoil Total RNA kit (QIAGEN, Germany). Suppliers of each kit used in RNA/DNA extraction processes can be found in Table S2.

RT-qPCR Procedures. Methods are found in our prior work.[34] Each reaction (total volume of 20 μL) contained 10 μL Power SYBR Green PCR Master Mix (Life Technologies, Warrington, UK), 0.1–1 μM primers, 0.5–2 μg bovine serum albumin (New England Biolabs, Ipswich, MA), and template DNA (Table S3). An ABI 7000 Sequence Detection System (Applied Biosystems, Grand Island, NY) was used for qPCR. The thermocycling conditions were as follows: 10 min at 95°C, 40 cycles at 95°C (15 s) and 60°C (1 min), and a PCR product dissociation step (i.e., a melt-curve). A *bphA* standard curve was prepared in triplicate (Table S4); for luciferase mRNA, standard DNA templates were gBlock fragments (Integrated DNA Technologies, Coralville, IA). Melt curve analysis indicated single peaks in standards and samples for *bphA* (85°C) and luciferase (81°C).

Table S2. Suppliers of DNA/RNA extraction and reverse transcription kits.

Processes	Kit	Supplier
RNA extraction	RNeasy PowerSoil Total RNA kit	QIAGEN, Germany
DNA extraction	RNeasy PowerSoil DNA Elution kit	QIAGEN, Germany
Luciferase mRNA control	N/A	Promega, Madison, WI
RNA purification	RNA Clean & Concentrator kit	Zymo Research, Irvine, California
Reverse transcription	SuperScript IV First-Strand Synthesis System	Invitrogen, Carlsbad, CA
Qubit 4 fluorometer	N/A	Thermo Fisher Scientific

Table S3. Primer sets used in qPCR analysis.

Target gene	Primer name	Sequences	Expected product size	Source
Luciferase mRNA	*ref* forward primer(1)	5'-TACAACACCCCAACATCTTCGA-3'	67 bp	Johnson et al., 2005[36]
	ref reverse primer(2)	5'-GGAAGTTCACCGGCGTCAT-3'		
bphA	*bphA* 463f	5'-CGCGTSGMVACCTACAARG-3'	211 bp	Petrić et al., 2011[37]
	bphA 674r	5'-GGTACATGTCRCTGCAGAAYTGC-3'		

(1) Nucleotide position 1691, (2) Nucleotide position 1758

Table S4. qPCR reagent mixture composition and parameters in accordance with MIQE guidelines.

Target gene	Primer conc (μM)	Bovine serum albumin (μg)	Template	qPCR linear range, gene copies/reaction	qPCR efficiency	Y-intercept
Luciferase mRNA	0.1	0.5	2 μL cDNA	30 - 3 × 10^7	89.01-99.42%	36.03-40.77
bphA	1	2	10 ng DNA or 2 μL cDNA	30 - 3 × 10^7	89.02-98.23%	35.72-38.22

QA/QC. The mRNA recovery efficiency (i.e., the ratio of measured luciferase cDNA abundance to luciferase transcripts added), was used to correct transcript abundance estimates for RT efficiency and mRNA losses during extraction and DNA digestion,[38] but is not accounted for absolute mRNA recovery. The mRNA recovery efficiency ranged from 0.043% to 3.5% (Figure S7-S8), where the efficiency is similar to other reports with most of the mRNA loss occurring during DNA digestion and RT.[34,38,39]

The entire RT-qPCR procedure was conducted in a biosafety cabinet (BSC). All supplies and BSC areas were wiped with RNase Away to remove RNase. All supplies were irradiated under UV light in the BSC for 20 minutes. All reagents, including Power SYBR green master mix, primers, DNA samples, and DNA standards, were thawed on ice to maintain the activity of reagents. DNA samples, which had been concentrated in previous steps, were diluted to 5-10 ng/μL using qPCR-grade water prior to analysis.

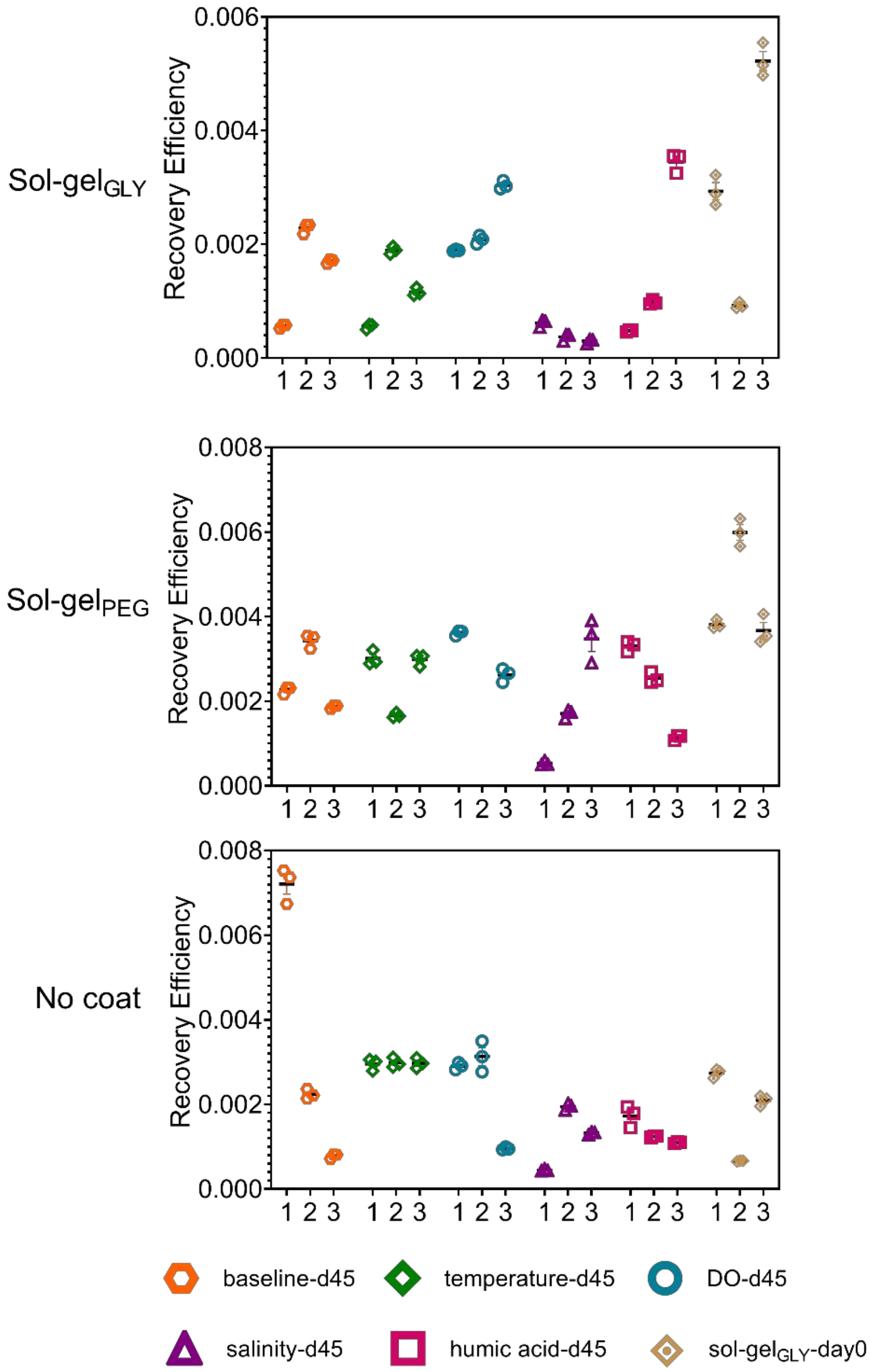

Figure S7. Recovery efficiencies of luciferase mRNA controls for sol-gel coated and uncoated biofilms at day-0 and day-45 under different environmental conditions. "Sol-gel$_{GLY}$" represents sol-gel$_{GLY}$-coated biofilms, "sol-gel$_{PEG}$" represents sol-gel$_{PEG}$-coated biofilms, and "no coat" represents uncoated biofilms. Environmental conditions include baseline conditions (fresh water, fully aerobic, room temperature, 20-21°C), temperature (10°C), dissolved oxygen (variable DO changes), salinity (20 g/L), and humic acid (2 mg/L). Error bars represent standard errors of three replicates mean (n=3).

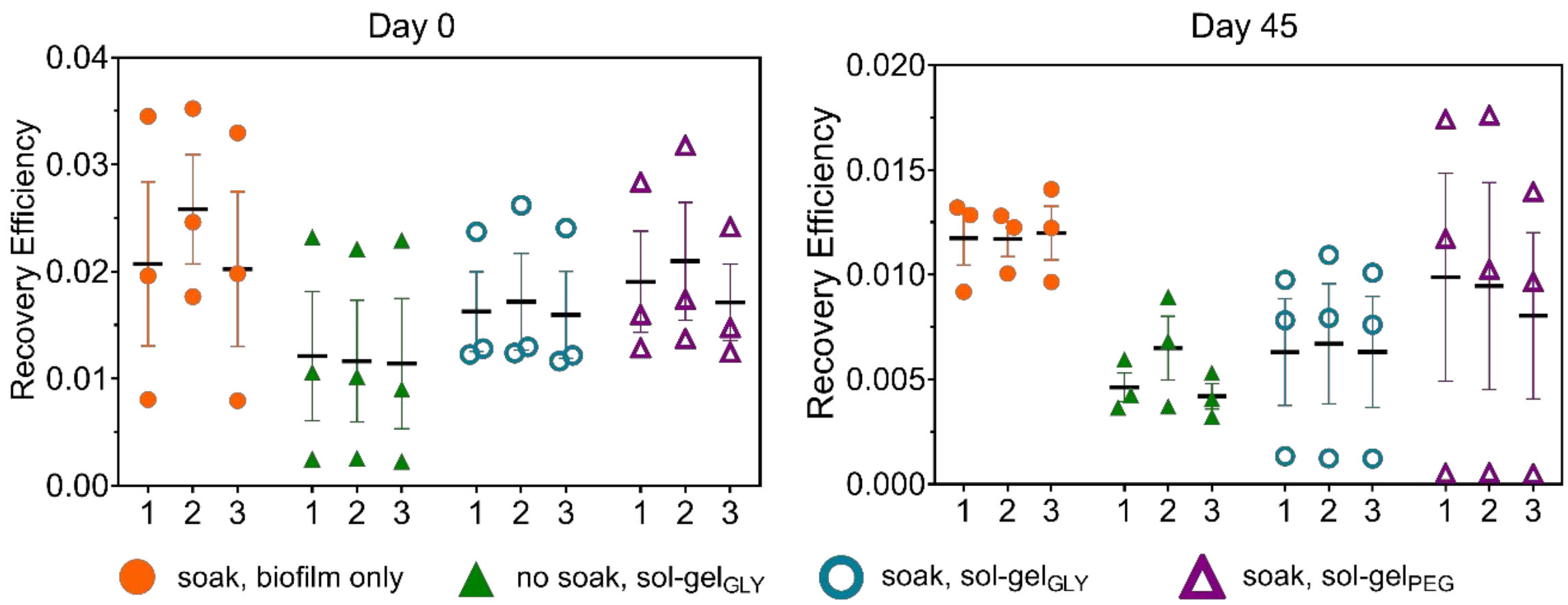

Figure S8. Recovery efficiencies of luciferase mRNA controls for sol-gel coated and uncoated, benzoate-soaked and unsoaked biofilms at day-0 and day-45 under standard conditions. Error bars represent standard errors of three replicates mean (n=3). Black lines represent mean of biological triplicates.

S1.9. Biofilm and Sol-Gel Visualization

Confocal Laser Scanning Microscopy (CLSM). Samples were stained with SYTO9 and propidium iodide for 30min, rinsed with phosphate-buffered saline (PBS), and then covered with 90% glycerol after rinsing. Reflectance and fluorescence imaging were observed under 488 nm and 499-600 nm, respectively. PBS was prepared by mixing 8 g NaCl, 0.2 g KCl, 1.44 g Na_2HPO_4, and 0.24 g KH_2PO_4 into 1 L DI water, and then adjusting the pH to 7.4.

Scanning Electron Microscopy (SEM). Dried samples (sol-gel coated and uncoated biofilms) were coated with silver solution (60% Ag in 1-methoxy-2-propanol, Electron Microscopy Sciences, Hatfield, PA) on an aluminum holder, and air dried in fume hood overnight. Before inserting samples into the SEM chamber, all samples were mounted with a conductive layer (5nm) of iridium sputter (EMS 150T ES, Quorum Technologies, Lewes, United Kingdom).

S2. Supplemental Results

S2.1. PCB Sorption

Sol-Gel and Biochar Sorbed 80% of PCBs and Sol-Gel Did Not Significantly Limit PCB Mass Transfer. PCBs with more chlorines - PCB18 (3 chlorines) and PCB52 (4 chlorines) - had more consistent K_d values (C_0 and C_e in Table S5) compared to PCB4 (2 chlorines; Figure S10). For PCB18 and PCB52, the rank of sorption potential followed: plain biochar > coated biochar > plain sol-gel > coated killed biofilm-biochar. These results indicate even though biochar (CKB) has a lower sorption capacity than granular activated carbon,[34] CKB still has relatively high PCB sorption potential. Interestingly, the sol-gel made by our recipe also had relatively high potential to sorb PCBs, where K_d of plain sol-gel was 5-39% of plain biochar K_d and increased with chlorine number. Complete sol-gel coatings on CKB could increase PCB mass transfer limitations from the bulk liquid to CKB if the sol-gel has high enough sorption potential to stop PCBs within sol-gel layers. However, there were no significant differences in PCB sorption between plain CKB and sol-gel coated CKB ($p>0.17$; Figure S9), and sol-gel had weaker sorption potential than CKB. This indicates that sol-gel did not significantly impact PCB sorption by CKB rather allowing PCB transfer from the bulk liquid across the sol-gel and to the biochar surface. Additionally, biochar inside the sol-gel had higher tendency to attract PCBs passing through sol-gel. This finding is promising that sol-gel not only helps sorb aqueous PCBs from the surrounding environment but also allows PCBs pass through sol-gel to inside layer.

Sorption potential was less dependent on glycerol/PEG400 addition in sol-gel (K_d of plain sol-gel$_{GLY}$ vs sol-gel$_{PEG}$ coating, $p>0.15$), but biofilm attachment hampered/decreased over 50% of sorption potentials of sorbents. Sol-gel$_{PEG}$-coated CKB exhibited higher sorption capacity for PCB18 (K_d: 6.32±1.50 vs 3.95±0.67, L/g) and PCB52 (K_d: 7.32±1.32 vs 3.88±0.39, L/g) but

similar sorption capacity for PCB4 (K_d: 1.95±0.32 vs 2.40±0.55, L/g) compared with sol-gel$_{GLY}$-coated CKB (Table S5). Sol-gel coated killed biofilms had significantly lower PCB sorption than plain biochar ($p<0.05$, Figure S9), especially for PCBs 18 and 52. This means biofilm attachment could hamper PCB sorption rather than sol-gel coating, which is reasonable because biofilms covered CKB and limited PCB interaction with CKB, and most PCB sorption was driven by biochar and sol-gel rather than cells.

As for PCB4, the discrepancy with PCBs 18 and 52 might be related to sorption capacity and the effectiveness of SPME to sorb PCB4, where PCBs with more chlorines had higher tendency to be on solids and SPME than less chlorines. Still, plain biochar had the highest sorption potential for PCB4, but sol-gel coating hampered 10-46% more mass transfer of PCB4 compared to PCBs 18 and 52. PEG400 coating impacts on PCB4 sorption had similar trend to that on PCB18 and PCB52, whereas gel layer of glycerol coating hampered PCB4 sorption on biochar and had low sorption on PCB4, less sorptive than biofilms. In summary, sol-gel coating had low sorption potential and minimal effects on PCB mass transfer. Although biofilm significantly interfered with PCB sorption on biochar, biofilm-CKB (sol-gel coating and no coating) could still sorb over 80% of PCBs (Figure S9).

Regarding sol-gel and CKB compositions in sol-gel coated CKB, we estimated based on dry weight of sorbents before and after sol-gel coating and concluded that the average mass composition of sol-gel$_{GLY}$ is 56.7% in sol-gel$_{GLY}$ coated CKB while the average mass composition of sol-gel$_{PEG}$ is 50.8% in sol-gel$_{PEG}$ coated CKB. Furthermore, we calculated expected sorption of PCBs on either sol-gel portion or CKB portion (Table S6) based on mass balance equations below using average K_d values of plain sol-gel$_{GLY}$, plain sol-gel$_{PEG}$, and uncoated CKB in Table S5. For example, for 40.84 ng of PCB4 in sol-gel$_{GLY}$ coated CKB (replicate 1), we expect 27.1 ng of

sorption on sol-gel and 39.4 ng of sorption on CKB. The sum of PCB sorption mass on each component, sol-gel + CKB, was not equal to the spiked PCB mass. The reason is that for this example we used average K_d values of plain sol-gel$_{GLY}$ rather than the actual K_d value for each component of combined sorbent, where the actual K_d value was not feasible to quantify but the approach was the best quantitative estimate of the PCB sorption by coatings and CKB.

For the mass balance of PCBs in the system,

$$C_0V = C_eV + q_eM$$

$$q_e = K_dC_e$$

Thus,

$$C_e = \frac{C_0V}{V + K_dM}$$

$$M_s = C_0V - C_eV = C_0V - \frac{C_0V}{V + K_dM}V$$

Where, C_0 is initial aqueous PCB concentration, C_e is equilibrium aqueous PCB concentration, q_e is equilibrium PCB concentration on sorbent, V is total volume of the system, M is mass of sorbent, and M_s is sorbed PCB mass.

For example, the amount of sorbed PCB4 mass on sol-gel$_{GLY}$ component (56.7% of sol-gel$_{GLY}$ coated CKB mass) when using average K_d values of plain sol-gel$_{GLY}$ should be the following:

$$C_0V = 1361.35ng/L \times 0.03L = 40.84ng$$

$$Kd = \frac{\frac{1.11L}{g} + \frac{0.67L}{g} + \frac{0.87L}{g}}{3} = \frac{0.883L}{g}$$

$$M_s = 40.84\,ng - \frac{40.84\,ng \times 0.03L}{0.03\,L + \frac{0.883\,L}{g} \times 0.1182\,g \times 56.7\%} = 27.1\,ng.$$

Table S5. Partition coefficients (K_d) of sorbents and water for PCBs 4, 18, and 52 after 10 days equilibration time. Raw data used to calculate K_d values for each sorbent (n=3), including sorbent mass, volume, initial PCB concentrations, and equilibrium PCB concentrations.

sample	replicate	calculated values			Measured values							
		partition coefficent, PCB4 Kd, L/g	partition coefficent, PCB18 Kd, L/g	partition coefficent, PCB52 Kd, L/g	sorbent mass M (g)	initial PCB4 concentration C0, PCB4 (ng/L)	initial PCB18 concentration C0, PCB18 (ng/L)	initial PCB52 concentration C0, PCB52 (ng/L)	equilibrium PCB4 concentration Ce, PCB4 (ng/L)	equilibrium PCB18 concentration Ce, PCB18 (ng/L)	equilibrium PCB52 concentration Ce, PCB52 (ng/L)	volume L (L)
sol-gel$_{GLY}$ coated killed biofilm	1	4.31	3.12	2.75	0.1005	1361.35	2410.22	4951.01	74.81	177.09	416.37	0.03
	2	3.74	2.43	2.05	0.104	892.25	1658.13	3524.66	82.67	214.97	524.53	0.03
	3	0.75	1.19	1.36	0.0993	1239.65	2058.39	4355.06	331.57	408.96	772.58	0.03
sol-gel$_{PEG}$ coated killed biofilm	1	0.47	1.69	2.21	0.1039	1361.35	2410.22	4951.01	439.68	294.95	490.13	0.03
	2	0.85	1.86	2.15	0.0688	892.25	1658.13	3524.66	392.23	384.56	717.4	0.03
	3	0.72	1.89	2.19	0.1449	1239.65	2058.39	4355.06	258.95	200.35	366.51	0.03
uncoated killed biofilm	1	2.32	2.65	2.13	0.1293	1361.35	2410.22	4951.01	105.16	163.14	417.4	0.03
	2	1.95	2.46	2.17	0.1258	892.25	1658.13	3524.66	126.08	178.88	421.41	0.03
	3	1.43	1.78	1.52	0.1274	1239.65	2058.39	4355.06	163.09	236.73	569.65	0.03
sol-gel$_{GLY}$ coated CKB	1	1.76	3.84	3.99	0.1182	1361.35	2410.22	4951.01	145.31	125.54	254.21	0.03
	2	2.65	3.35	3.44	0.097	892.25	1658.13	3524.66	120.51	171.22	350.16	0.03
	3	2.78	4.67	4.2	0.1159	1239.65	2058.39	4355.06	98.49	106.46	246.84	0.03
sol-gel$_{PEG}$ coated CKB	1	2.31	7.89	8.74	0.123	1361.35	2410.22	4951.01	110.22	60.7	115.29	0.03
	2	1.83	6.17	7.09	0.1304	892.25	1658.13	3524.66	129.02	72.83	133.61	0.03
	3	1.7	4.9	6.13	0.1271	1239.65	2058.39	4355.06	141	93.16	157.47	0.03
uncoated CKB	1	11.29	17.13	13.89	0.1276	1361.35	2410.22	4951.01	23.57	27.42	70.71	0.03
	2	31.59	19.49	14.2	0.1248	892.25	1658.13	3524.66	8.72	24.67	70.73	0.03
	3	5.26	10.34	9.48	0.1283	1239.65	2058.39	4355.06	49.12	44.78	102.24	0.03
plain sol-gel$_{GLY}$	1	1.11	3.57	5.66	0.2699	1361.35	2410.22	4951.01	104.9	61.22	81.85	0.03
	2	0.67	2.7	4.23	0.2398	892.25	1658.13	3524.66	181.2	89.81	122.01	0.03
	3	0.87	2.92	4.84	0.184	1239.65	2058.39	4355.06	182.89	106.95	138.51	0.03
plain sol-gel$_{PEG}$	1	1.68	3.85	5.86	0.2289	1361.35	2410.22	4951.01	83.38	66.68	92.89	0.03
	2	0.87	2.06	2.62	0.23	892.25	1658.13	3524.66	151.17	120.54	201.73	0.03
	3	1.81	3.36	4.53	0.2921	1239.65	2058.39	4355.06	62.08	60.12	94.23	0.03

Table S6. Estimated PCB mass sorbed on sol-gel coated CKB components (i.e., sol-gel only or CKB only) based on sol-gel mass composition, CKB mass composition, plain sol-gel K_d, and uncoated CKB K_d values in Table S5. The mass composition of sol-gel$_{GLY}$ in sol-gel$_{GLY}$ coated CKB is 56.7%. The mass composition of sol-gel$_{PEG}$ in sol-gel$_{PEG}$ coated CKB is 50.8%. The quantified PCB mass sorbed on sol-gel+CKB is provided for comparison.

Sample	Replicate	Quantified initial PCB4 mass (ng)	Quantified initial PCB18 mass (ng)	Quantified initial PCB52 mass (ng)	PCB4 mass (ng)			PCB18 mass (ng)			PCB52 mass (ng)		
					estimated PCB4 sorbed on sol-gel	estimated PCB4 sorbed on CKB	quantified PCB4 sorbed on sol-gel + CKB	estimated PCB18 sorbed on sol-gel	estimated PCB18 sorbed on CKB	quantified PCB18 sorbed on sol-gel + CKB	estimated PCB52 sorbed on sol-gel	estimated PCB52 sorbed on CKB	quantified PCB52 sorbed on sol-gel + CKB
sol-gel$_{GLY}$ coated CKB	1	40.84	72.31	148.53	27.10	39.40	36.48	63.09	69.70	68.54	136.12	141.89	140.90
	2	26.77	49.74	105.74	16.55	25.63	23.15	42.23	47.57	44.61	95.17	100.03	95.24
	3	37.19	61.75	130.65	24.52	35.85	34.23	53.74	59.48	58.56	119.54	124.70	123.25
sol-gel$_{PEG}$ coated CKB	1	40.84	72.31	148.53	30.70	39.58	37.53	62.58	70.02	70.49	133.73	142.65	145.07
	2	26.77	49.74	105.74	20.41	25.99	22.90	43.39	48.26	47.56	95.74	101.79	101.73
	3	37.19	61.75	130.65	28.18	36.08	32.96	53.68	59.86	58.96	118.01	125.65	125.93

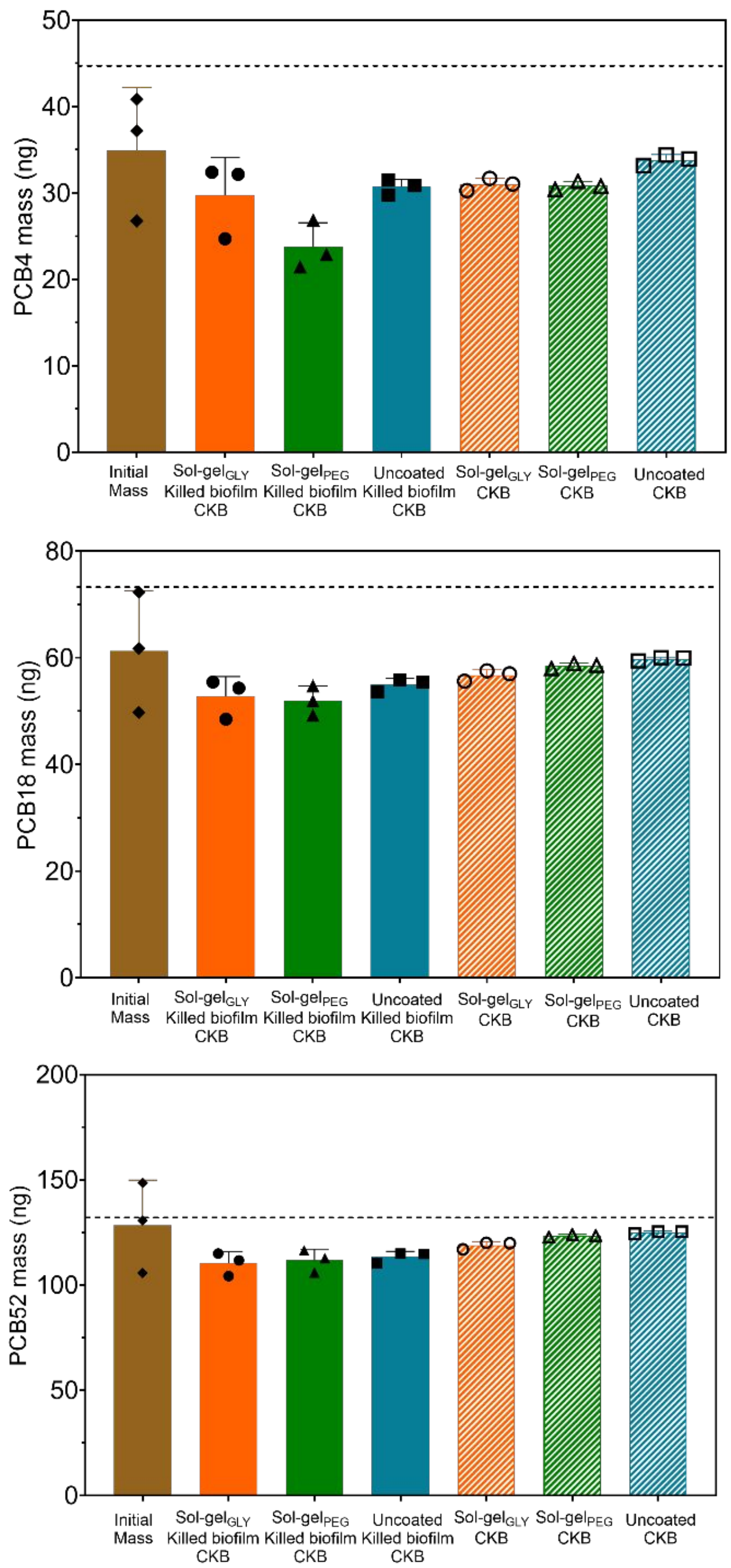

Figure S9. Mass of PCBs (PCB4, PCB18, and PCB52) sorbed on different sorbents after 10 days equilibration time. Each test tube contained the same initial PCB mass as shown by "initial mass" (brown bars). Sorbents include sol-gel$_{GLY}$+killed biofilm+CKB, sol-gel$_{PEG}$+killed biofilm+CKB, uncoated killed biofilm+CKB, sol-gel$_{GLY}$+CKB, sol-gel$_{PEG}$+CKB, and uncoated CKB. Columns represent the mean of biological triplicates. Error bars represent the standard deviation of biological triplicates (n=3).

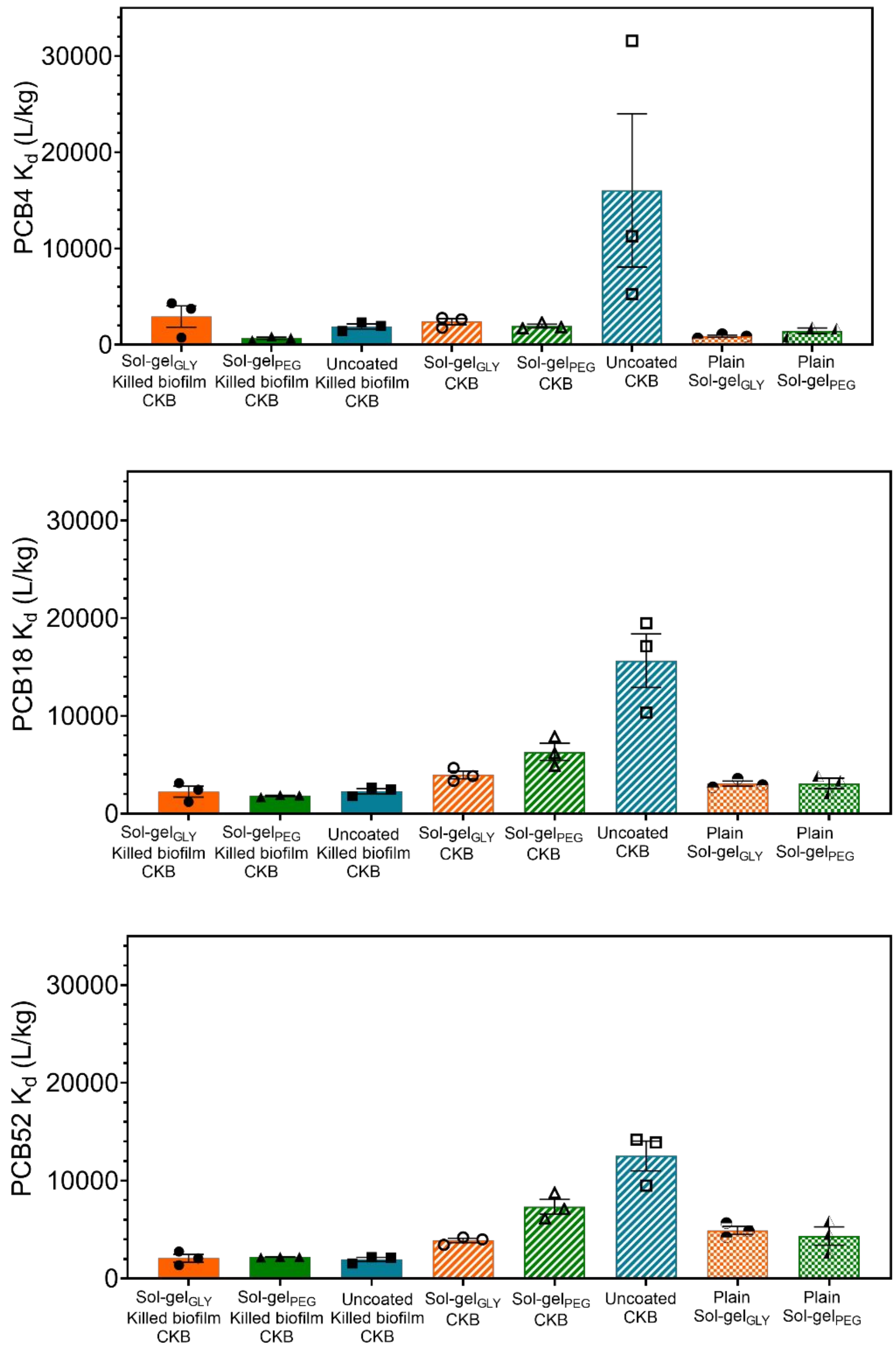

Figure S10. Partition coefficients (K_d) of PCB congeners (PCB4, PCB18, and PCB52) between each sorbent type and water. Orange columns represent sol-gel$_{GLY}$ coating, green bars represent sol-gel$_{PEG}$ coating, and blue bars represent no coating. Solid symbols with solid columns represent killed biofilms, hollow symbols with striped columns represent CKB, and half-hollow symbols with checkered columns represent plain sol-gel without biomass. Columns represent the mean of biological triplicates. Error bars represent the standard errors of biological replicates (n=3).

S2.2. PCB Biodegradation Rate

Soaking with benzoate could inhibit PCB biodegradation performance of glycerol coated biofilms. Benzoate soaking did not improve biofilm biodegradation performance and efficiency (PCB biodegradation rate between sol-gel$_{GLY}$+soak and sol-gel$_{GLY}$ + no soak was not different, $p>0.4$), and even inhibited aqueous PCB4 ($p=0.004$, day 45) and PCB18 ($p=0.012$, day 45) biodegradation over extended treatment times (Figure 1). Biofilms on benzoate-soaked CKB degraded significantly less (no soak/soak=72-88%, $p<0.04$) total PCBs (aqueous+solid phase), for each PCB congener in the initial treatment period (d=3), and degraded less PCB4 (no soak/soak=37%, $p=0.035$) by the end of treatment (d=45)(Figure 1).

Benzoate soaking was beneficial to biofilm viability before treatment but less helpful for long-term treatment. Prior to PCB biodegradation at day-0, benzoate soaking (sol-gel coating and no coating) had more live cells than dead cells (ranging from 2:1 to almost all live), whereas no soaking group was opposite (~1:2 live to dead cells). Interestingly, the sol-gel$_{GLY}$ +no soak treatment had more live cells than dead cells (2:1) and >2 times more abundant cells than uncoated biofilms and sol-gel$_{GLY}$ +soak biofilms at day-45. Benzoate soaking had no impacts on sol-gel$_{GLY}$ gel integrity (e.g., 500μm and 100μm, Figure S17-S18) and porosity.

This phenomenon may be related to the competition between benzoate and PCBs as carbon sources for biofilm consumption, where benzoate is more easily biodegraded than PCBs. The pathway LB400 cells use for benzoate is different from PCBs; thus, there might be additional energy required for pathway differences.[40] LB400 is generally not able to use PCBs as sole carbon and energy sources, except for a few LC-PCB congeners (e.g., monochlorobiphenyls and dichlorobiphenyls such as PCB4 and PCB8)[41,42], but rather cometabolize PCBs in the presence of other carbon sources (e.g., benzoate). While PCB4 can be used as a carbon and energy source by

LB400, the competition between two carbon sources could last longer. Another potential explanation is that soaking process caused LB400 abundance loss (p=0.056, Figure S19), thus the cell abundance of no soaking before treatment was 1.8-fold of soaking groups. This finding is interesting because over 85% of spiked PCB could be degraded by sol-gel$_{GLY}$-coated biofilms without extra carbon source addition. Further investigation on potential carbon sources in sol-gel will be discussed.

Table S7. PCB degradation rate of sol-gel coated and uncoated biofilms based on total PCB mass removal (aqueous + solid phase) over time. One-phase decay model (Figure S12-S14) was fitted to determine parameters.

one phase decay	no soak, sol-gel$_{GLY}$ coat			soak, biofilm only			soak, sol-gel$_{GLY}$ coat			soak, sol-gel$_{PEG}$ coat		
	PCB4	PCB18	PCB52	PCB4	PCB18	PCB52	PCB4	PCB18	PCB52	PCB4	PCB18	PCB52
Y$_0$_ng	70.19	69.29	149.20	68.40	61.75	144.70	73.40	75.46	169.00	70.68	74.69	168.20
plateau_ng	5.46	10.43	25.34	4.93	7.98	19.17	11.99	14.10	20.75	0.29	-15.19	2.44
k_1/d	0.11	0.09	0.12	0.79	0.61	0.49	0.11	0.08	0.09	0.04	0.02	0.04
half life_d	6.39	8.13	5.79	0.88	1.13	1.41	6.07	8.33	8.14	16.31	28.92	15.91
Tau_d	9.22	11.73	8.35	1.26	1.63	2.03	8.76	12.02	11.75	23.54	41.72	22.95
Span_ng	64.73	58.85	123.90	63.47	53.77	125.60	61.41	61.37	148.30	70.39	89.88	165.70
R square	0.98	0.90	0.97	0.99	0.99	0.98	0.95	0.81	0.87	0.94	0.85	0.92

Note: Y_0 represents PCB mass at day-0; plateau represents PCB mass at infinite time; k represents the first-order biodegradation rate constant; half-life = ln(2)/k; Tau: the reciprocal of k; Span: the difference between Y_0 and plateau.

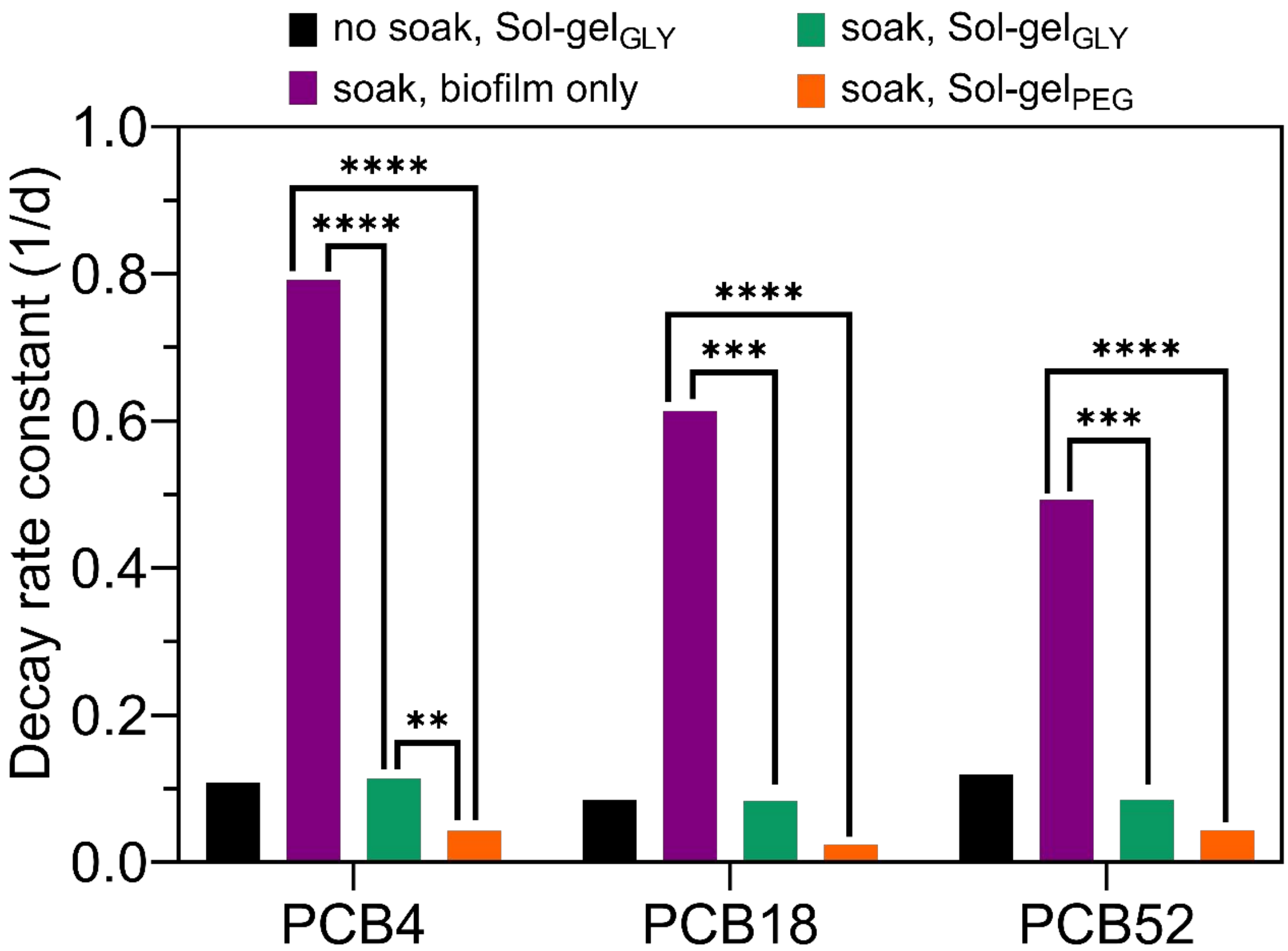

Figure S11. PCB biodegradation rate constant (1/d, one phase decay) for each PCB congener. "**" represents $p<0.01$, "***" represents $p<0.001$, "****" represents $p<0.0001$.

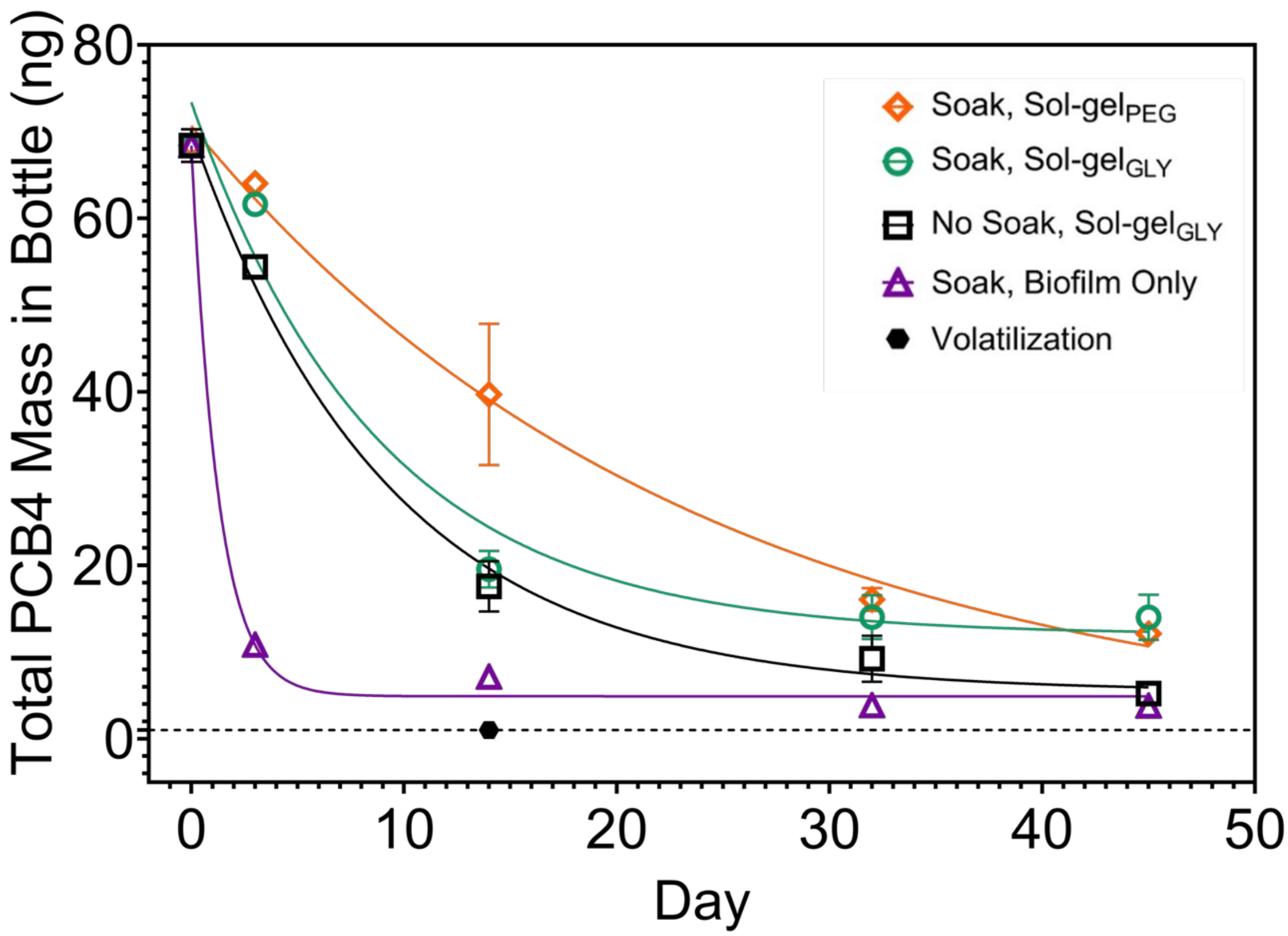

Figure S12. First order decay curve fitting of total PCB4 biodegradation under standard conditions. This figure corresponds to Figure 1 in the main manuscript.

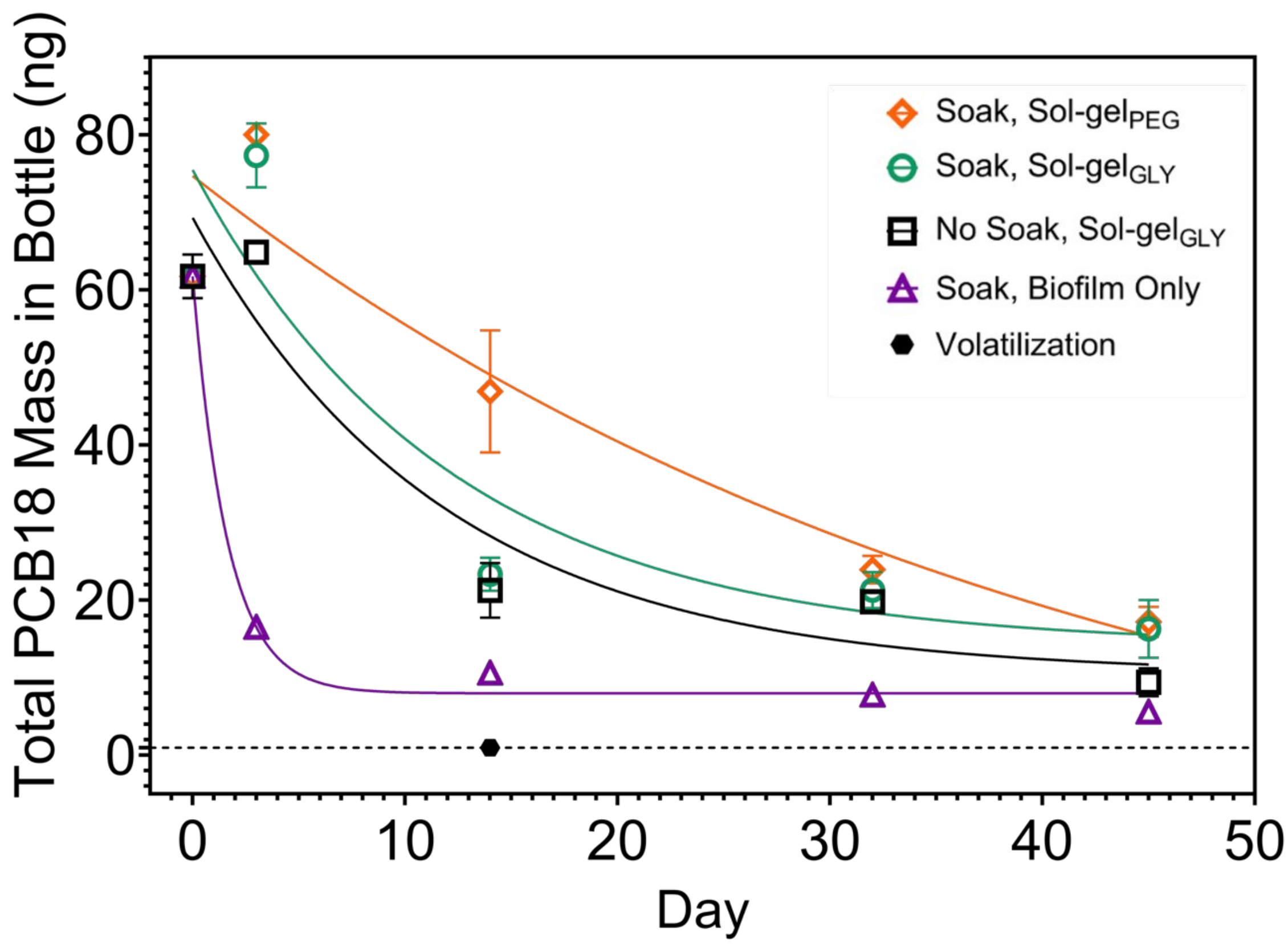

Figure S13. First order decay curve fitting of total PCB18 biodegradation under standard conditions. This figure corresponds to Figure 1 in the main manuscript.

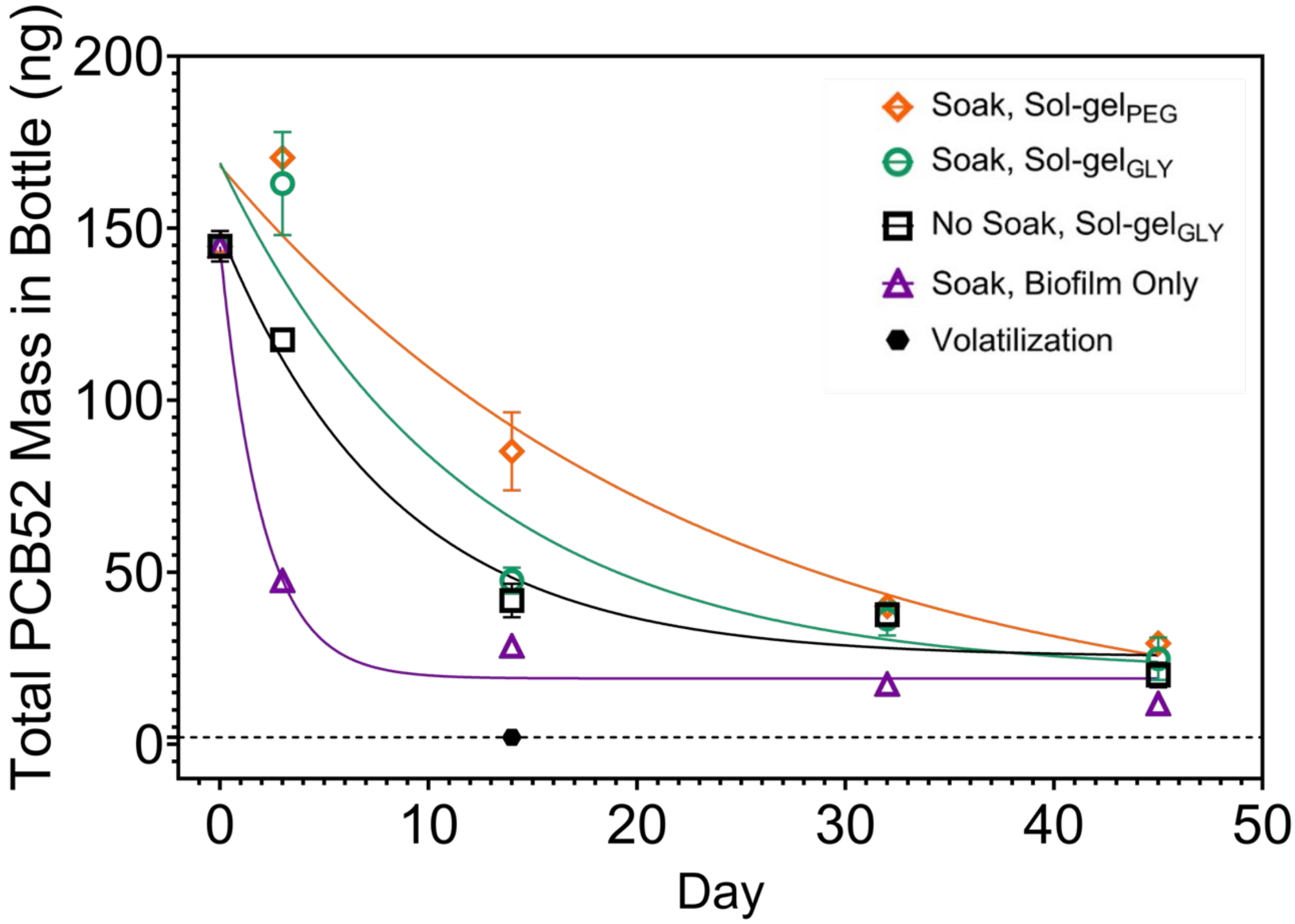

Figure S14. First order decay curve fitting of total PCB52 biodegradation under standard conditions. This figure corresponds to Figure 1 in the main manuscript.

Table S8. Average PCB mass (ng) change of biotic treatments corresponding to Figure 1 and Figure S15: aqueous phase, solid phase, and sum of aqueous and solid phases.

		no soak, sol-gelGLY			soak, biofilm only			soak, sol-gelGLY			soak, sol-gelPEG		
	Day	PCB4 (ng)	PCB18 (ng)	PCB52 (ng)	PCB4 (ng)	PCB18 (ng)	PCB52 (ng)	PCB4 (ng)	PCB18 (ng)	PCB52 (ng)	PCB4 (ng)	PCB18 (ng)	PCB52 (ng)
aqueous + solid	0	68.40	61.75	144.79	68.40	61.75	144.79	68.40	61.75	144.79	68.40	61.75	144.79
	3	54.42	64.85	117.60	10.82	16.51	47.58	61.64	77.33	163.04	64.04	80.01	170.54
	14	17.59	21.26	41.80	7.13	10.62	28.55	19.56	23.34	47.62	39.71	46.91	85.15
	32	9.23	19.80	37.72	3.85	7.77	17.45	14.05	21.28	36.44	16.07	23.95	40.18
	45	5.23	9.40	20.13	3.80	5.58	11.77	14.01	16.29	24.87	12.13	17.20	29.40
aqueous	3	0.13	0.23	2.40	0.11	0.12	2.07	0.30	0.36	3.05	0.38	0.51	3.04
	14	0.01	0.06	0.90	0.00	0.01	0.09	0.03	0.11	1.11	0.06	0.17	1.54
	32	0.04	0.52	1.85	0.00	0.01	0.09	0.04	0.37	1.68	0.01	0.05	0.26
	45	0.05	0.38	1.26	0.02	0.02	0.09	0.23	0.76	1.86	0.05	0.10	0.33
solid	3	54.29	64.62	115.20	10.71	16.39	45.51	61.34	76.98	159.99	63.66	79.49	167.50
	14	17.58	21.19	40.90	7.13	10.61	28.46	19.53	23.23	46.51	39.65	46.74	83.62
	32	9.20	19.28	35.86	3.85	7.75	17.36	14.01	20.92	34.76	16.06	23.91	39.92
	45	5.18	9.02	18.87	3.78	5.56	11.68	13.79	15.53	23.00	12.08	17.10	29.06

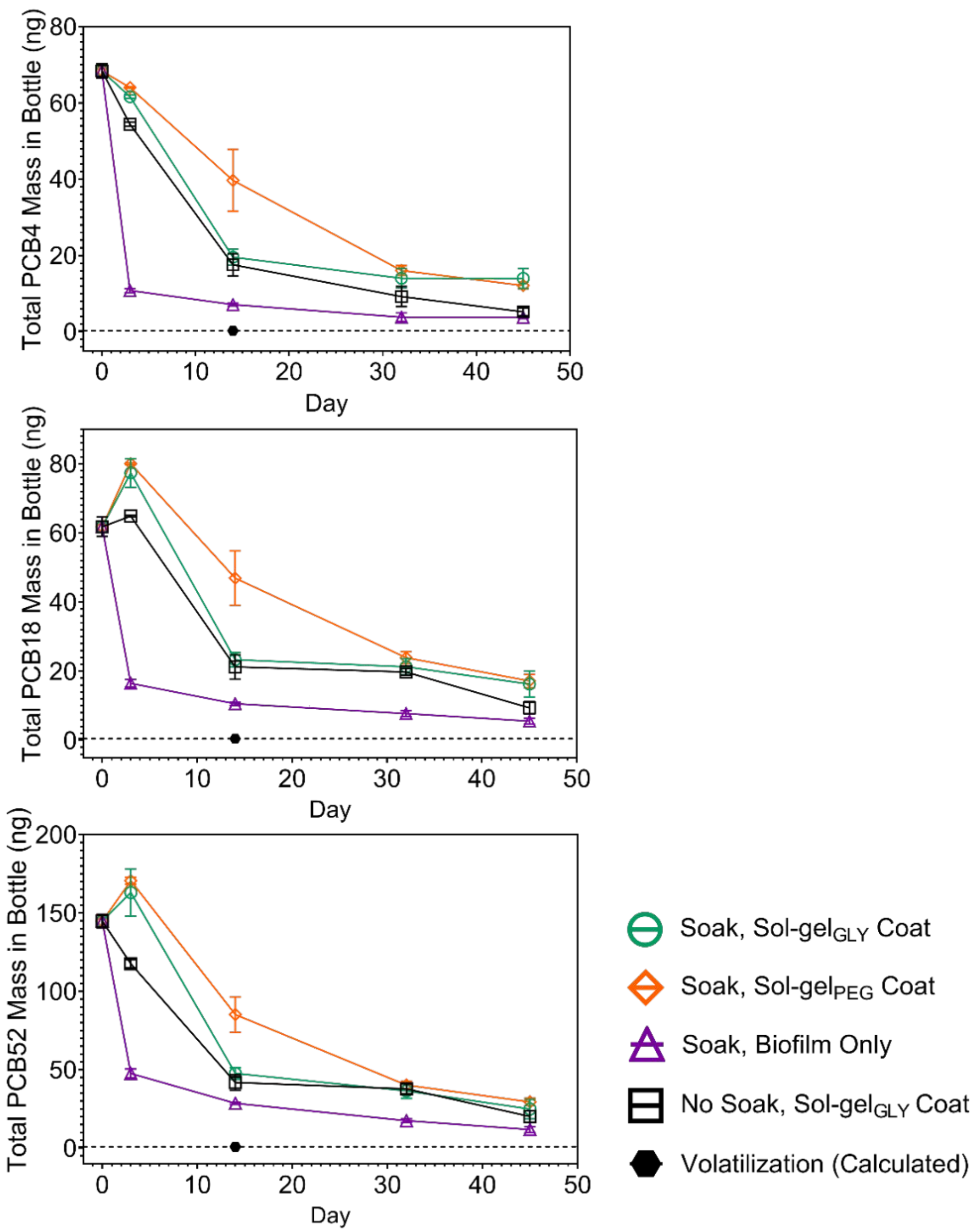

Figure S15. Changes in total PCB congener mass over time in the presence of uncoated and sol-gel coated LB400 biofilms on CKB: PCB4 (row 1), PCB 18 (row 2), and PCB52 (row 3). PCBs in total were summed by aqueous and solid phase PCB mass in Figure 1 in the main text. Data points are in Table S8. Each panel includes five groups: hollow green circles represent "benzoate soak + Sol-gel$_{GLY}$ coating"; black squares represent "no soak + Sol-gel$_{GLY}$ coating"; purple triangles represent "benzoate soak + no sol-gel coating"; hollow orange diamonds represent "benzoate soak + Sol-gel$_{PEG}$ coating"; black hexagons ("volatilization") and dash lines represent calculated PCB volatilization to head space at equilibrium based on Henry's law (S2.4).

S2.3. Biofilm viability and images of testing sol-gel coated and uncoated biofilms under standard conditions

Table S9. Cell Viability of Sol-Gel Coated and Uncoated Biofilms Under Standard Conditions at Day-0 and Day-45.

			$Sol\text{-}gel_{GLY}$ coat, soak	$Sol\text{-}gel_{PEG}$ coat, soak	$Sol\text{-}gel_{GLY}$ coat, no soak	Uncoated
day-0	live	1	830	788	159	118
		2	2738	229	966	204
	dead	1	159	359	186	48
		2	161	107	1772	100
day-45	live	1	484	290	1196	207
		2	497	721	1159	125
	dead	1	426	1944	1830	311
		2	635	2072	396	690

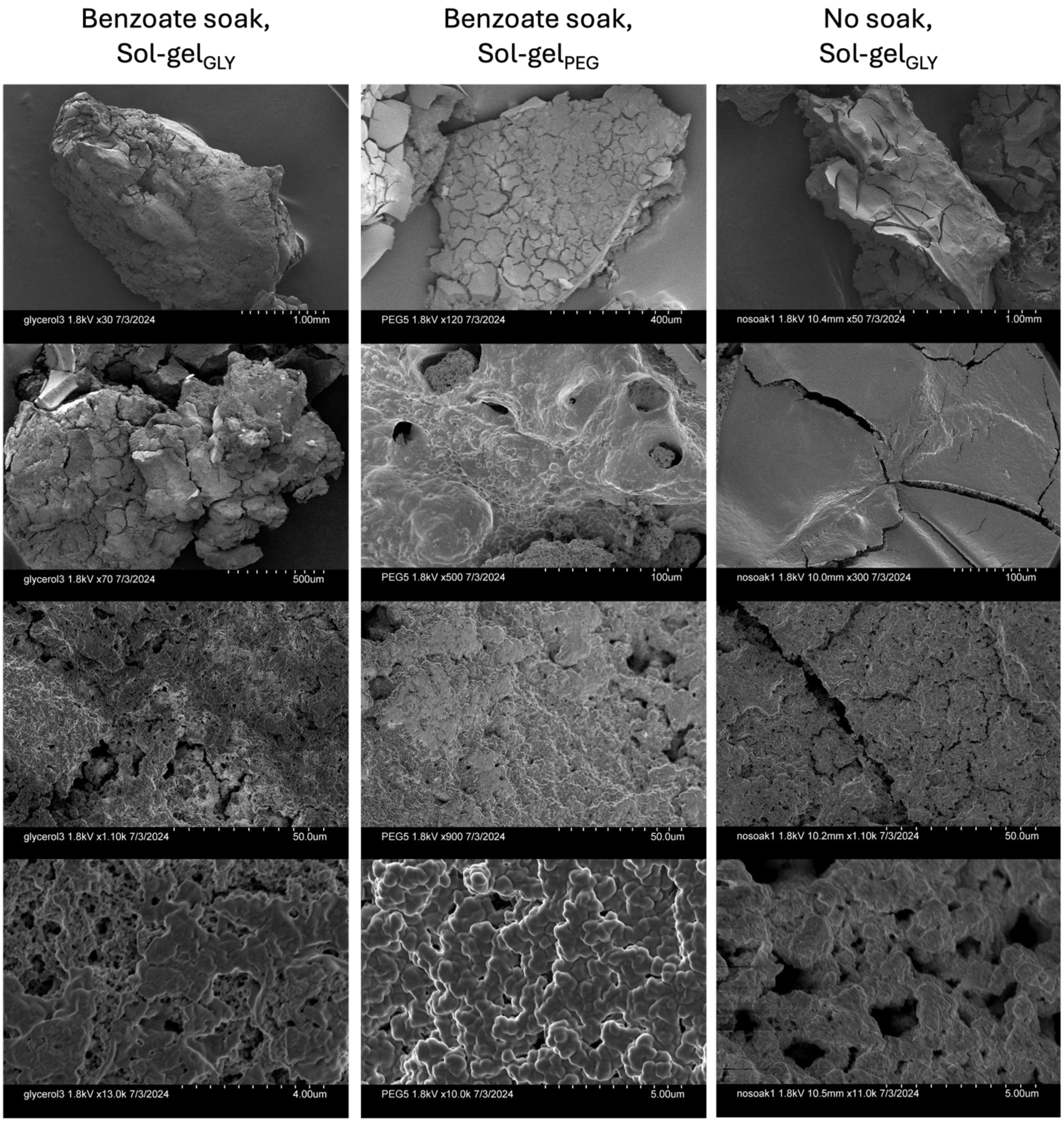

Figure S16. SEM images: surface of sol-gel coated biofilms before shaking. Three columns represent 3 groups: 1st column represents "benzoate soak + sol-gel$_{GLY}$"; 2nd column represents "benzoate soak + sol-gel$_{PEG}$"; 3rd column represents "no soak + sol-gel$_{GLY}$". Note that images had different magnifications.

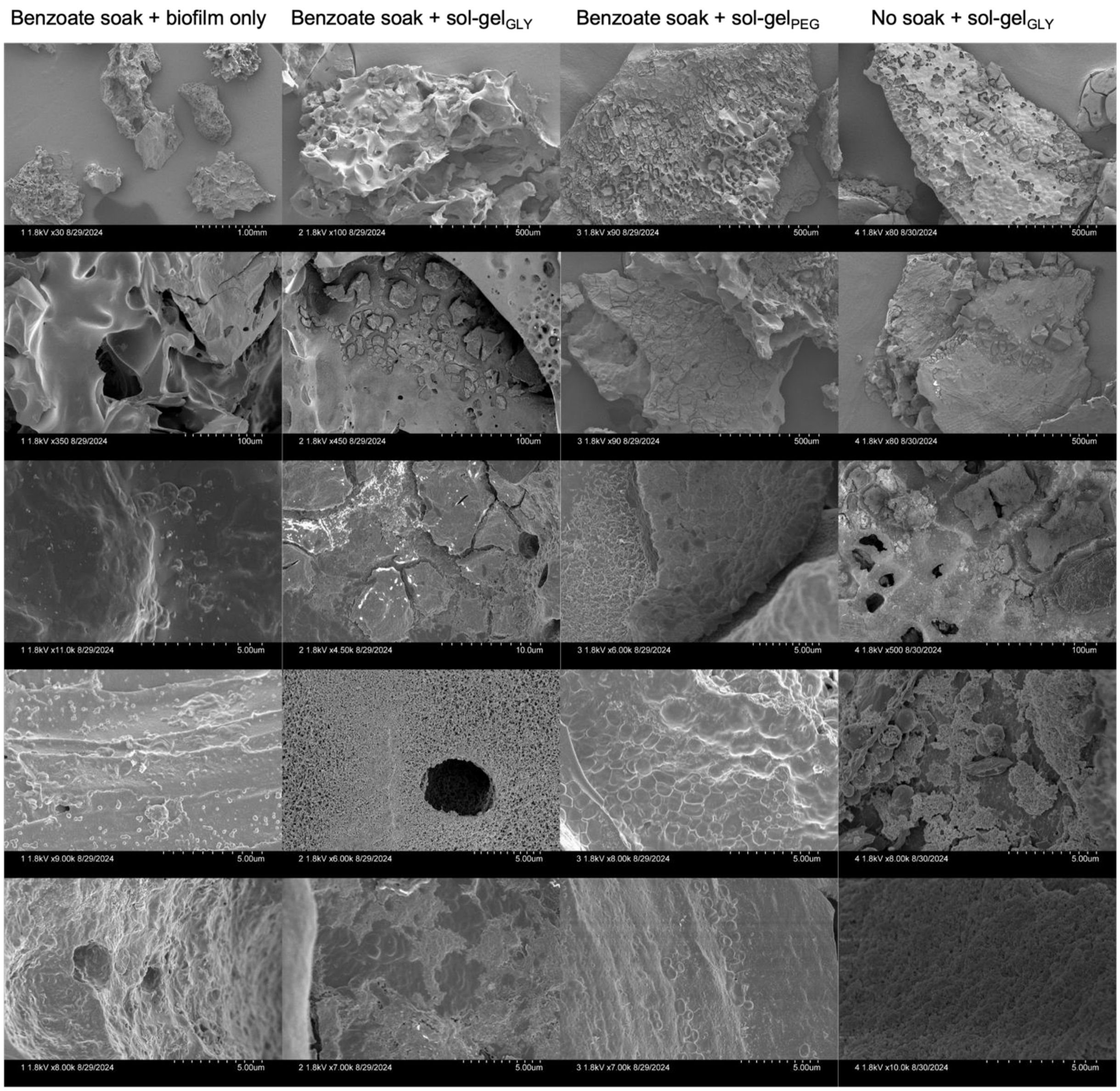

Figure S17. SEM images: surface of sol-gel coated and uncoated biofilms after shaking for 45 days in PCB solution under standard conditions. Four columns represent 4 groups: 1st column represents "benzoate soak + biofilm only"; 2nd column represents "benzoate soak + sol-gel$_{GLY}$"; 3rd column represents "benzoate soak + sol-gel$_{PEG}$"; 4th column represents "no soak + sol-gel$_{GLY}$". Note that images had different magnifications.

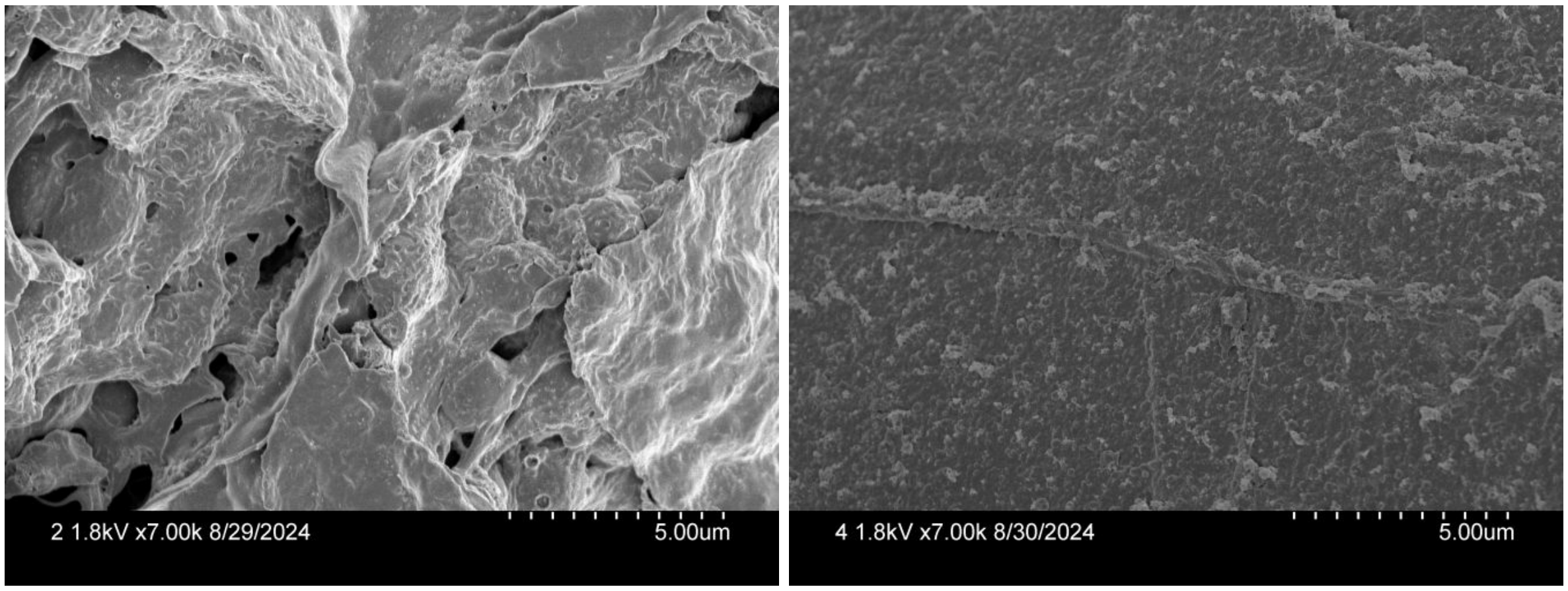

Figure S18. Suspected biochar surface with no sol-gel coverage after 45-days of treatment under standard conditions. Left: sol-gel$_{GLY}$ + benzoate soak; right: sol-gel$_{GLY}$ + no soak.

S2.4. Henry's law calculation to estimate PCB volatilization

Henry's law constant at room temperature at 20°C.[43]

$H_{PCB4} = 3.3 \times 10^{-2} mol/(m3Pa)$

$H_{PCB18} = 3.3 \times 10^{-2} mol/(m3Pa)$

$H_{PCB52} = 4.1 \times 10^{-2} mol/(m3Pa)$

Henry's law calculation to estimate the maximum amount of volatilization at 20°C.

$m_{air} = \frac{PV}{RT} = \frac{C_{water}V}{HRT}$

$m_{air,\,PCB4} = 0.290ng$

$m_{air,\,PCB18} = 0.508ng$

$m_{air,\,PCB52} = 0.856ng$

S2.5. *BphA* abundance and expression under standard condition test

Although sol-gel$_{GLY}$-coated biofilms without soaking had two times higher *bphA* transcripts than sol-gel$_{GLY}$+soak biofilms ($p<0.0001$), one of the soaked treatments had 3 orders of magnitude lower *bphA* abundance than no soaking. The high variance in *bphA* abundance resulted in over 3 orders of magnitude higher average *bphA* gene expression level ($p>0.05$) under benzoate soaking condition at day-0, while no soaking groups had less active biofilms (transcript per gene ratio <1).

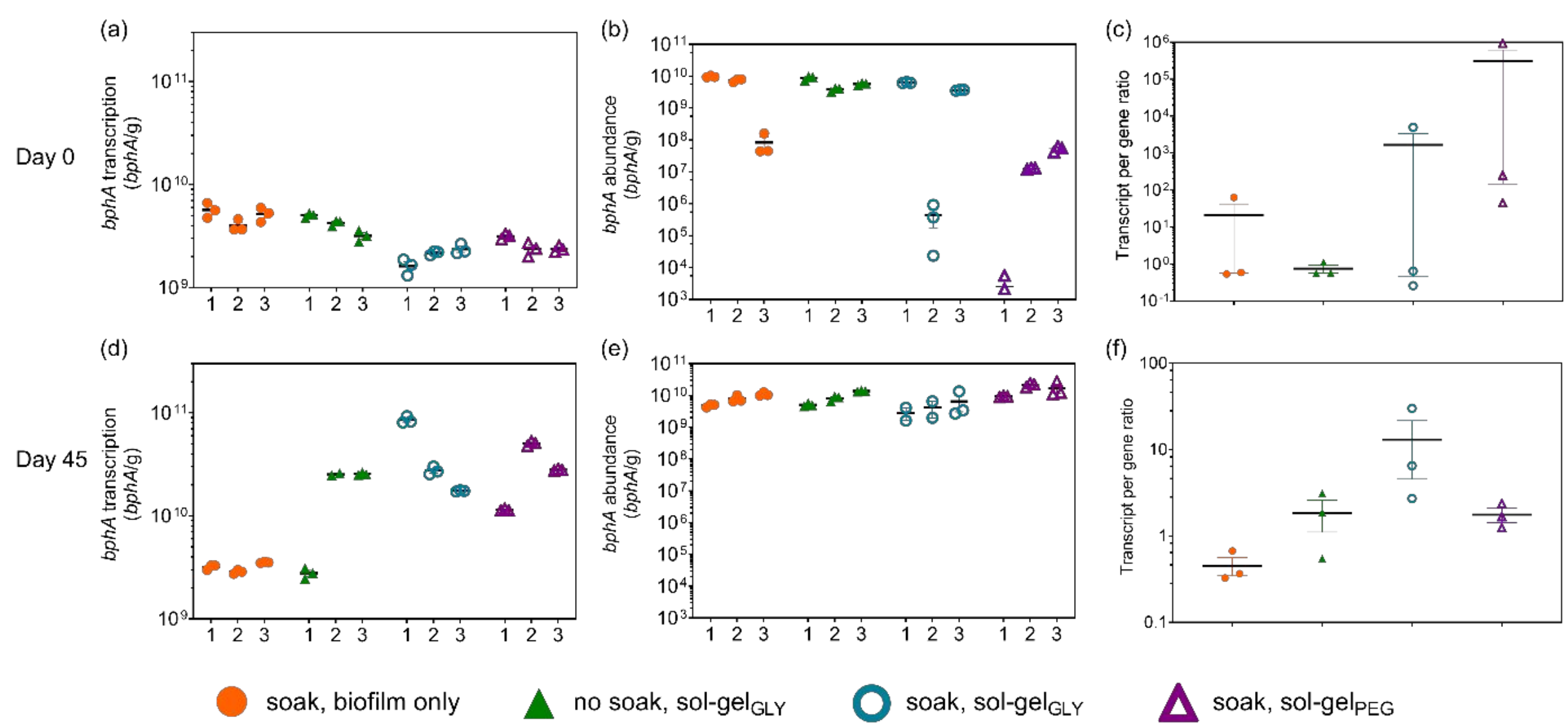

Figure S19. *BphA* expression and abundance of sol-gel coated and uncoated biofilms under standard conditions at day-0 and day-45. (a) *BphA* transcripts (per gram of sol-gel coated or uncoated biofilm-CKB) differences among four groups at day 0. (b) *BphA* abundance (per gram of sol-gel coated or uncoated biofilm-CKB) differences among four groups at day 0. (c) Gene expression level (transcript per gene ratio) differences among four groups at day 0. (d) *BphA* transcripts (per gram of sol-gel coated or uncoated biofilm-CKB) differences among four groups at day 45. (e) *BphA* abundance (per gram of sol-gel coated or uncoated biofilm-CKB) differences among four groups at day 45. (f) Gene expression level (transcript per gene ratio) differences among four groups at day 45. Solid orange circles represent “biofilm only + soak” control groups; solid green triangles represent “sol-gel$_{GLY}$ + no soak” control groups; open blue circles represent “sol-gel$_{GLY}$ + soak” treatment groups; open purple triangles represent “sol-gel$_{PEG}$ + soak” treatment groups. Outlier test was conducted to remove outliers. Error bars represent standard error of biological triplicates, and black lines represent the mean of biological triplicates.

S2.6. Environmental condition impacts on sol-gel coated biofilm performance

Sorbent-associated PCB removal was higher in uncoated biofilms (Figure 4f, under all conditions except PCBs 18 and 52 at 10°C and 2mg/L humic acid) and sol-gel_{GLY}-coated biofilms (Figure 4d, baseline and DO changes), compared to abiotic controls (killed biofilms and sol-gel_{GLY}-coated killed biofilms on CKB; Figure S21). Humic acid induced higher *bphA* expression levels on sol-gel_{GLY}-coated biofilms than DO (p=0.042)(Figure 4d), while DO induced higher *bphA* expression levels on uncoated biofilms than salinity (p=0.011)(Figure 4f). During 45-days of PCB treatment, only sol-gel_{GLY}-coated biofilms showed large DO changes, from fully aerobic to anaerobic, while sol-gel_{PEG}-coated and uncoated biofilms displayed only about 1 mg/L oxygen decreases (Figure S20). This indicates that variable DO levels could negatively affect biofilm activity in PCB biodegradation while more aerobic conditions helped maintain higher biofilm activity regardless of sol-gel coating (transcript per gene ratio >10 for both sol-gel_{PEG} and no coating).[44] There were no significant impacts of temperature on *bphA* expression levels in uncoated biofilms compared to all other environmental conditions (p>0.057)(Figure 4f).

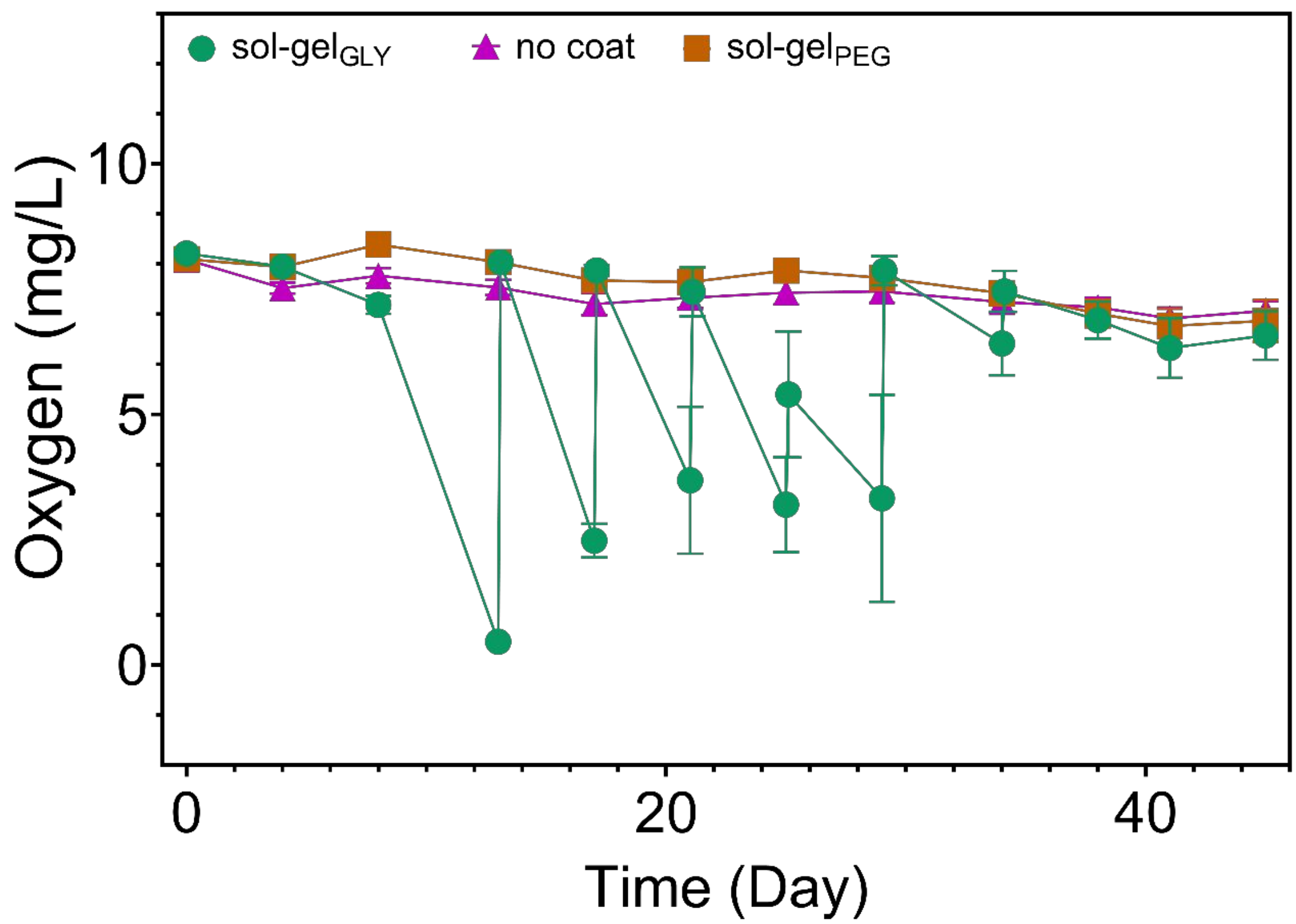

Figure S20. Dissolved oxygen changes over 45 days for biofilms with different sol-gel coatings. Three sol-gel coating conditions include sol-gel$_{GLY}$, sol-gel$_{PEG}$, and no coating. Each data point represents the mean of biological triplicates, and error bars represent standard error of biological triplicates (n=3). Oxygen was measured and filled once dissolved oxygen concentrations were below 6 mg/L. Only sol-gel$_{GLY}$-coated biofilms required oxygen replenishment, whereas sol-gel$_{PEG}$-coated biofilms and uncoated biofilms show low DO consumption.

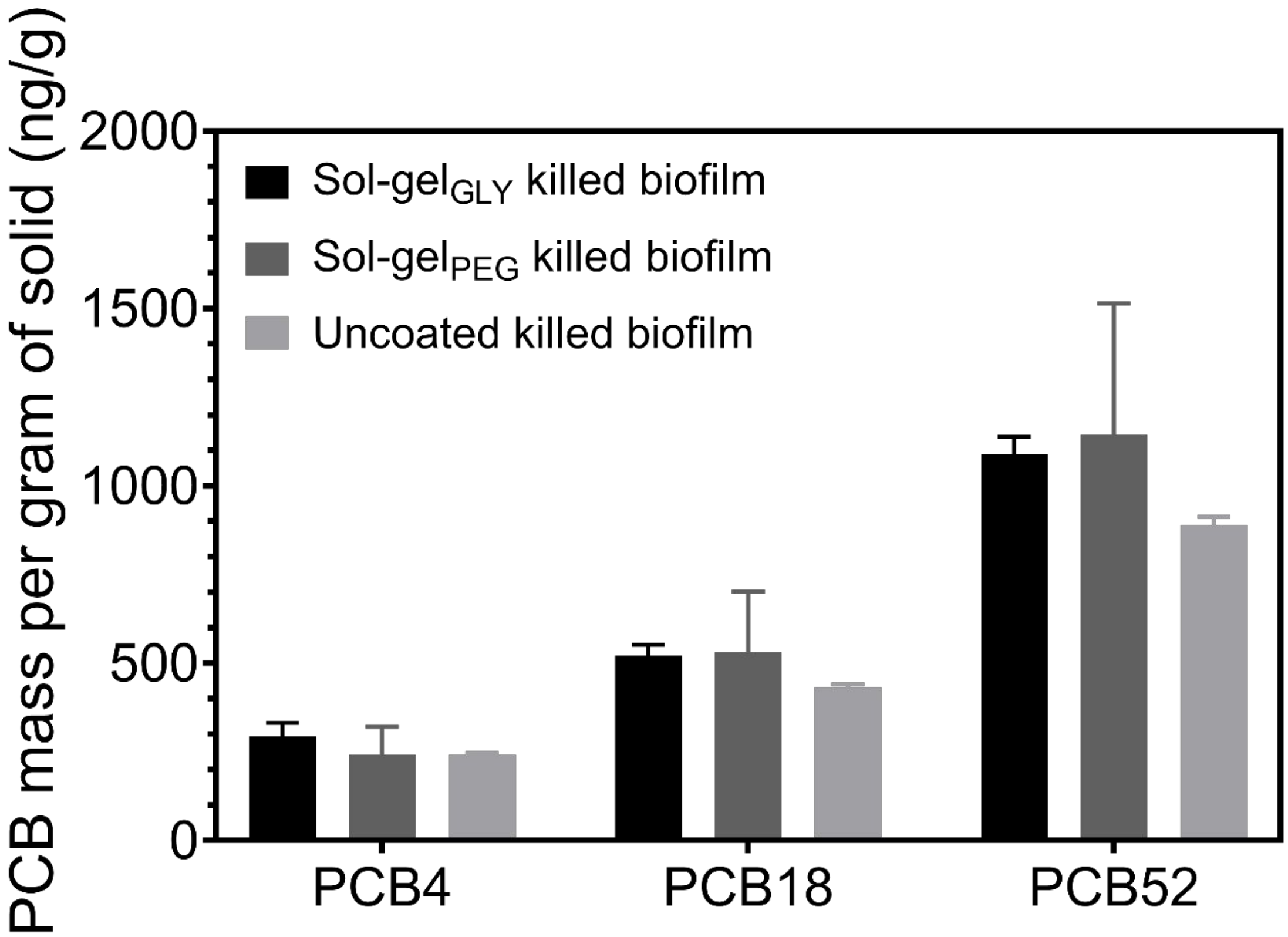

Figure S21. Abiotic controls for PCBs 4, 18, and 52 biodegradation performance under different environmental conditions. PCB mass associated with sorbents normalized by sample mass, including three sol-gel coating conditions of killed biofilm-CKB: sol-gel$_{GLY}$ coating, sol-gel$_{PEG}$ coating, and no coating. Error bars represent standard errors of three replicates mean (n=3). "Killed biofilm" also refers to abiotic controls of sol-gel coated or uncoated killed biofilm-CKB in Figure 1. These abiotic controls cross-reference the biotic treatments described in Figure 4.

<u>*Humic acid was beneficial to bphA transcription of sol-gel coated biofilms.*</u> Environmental conditions and sol-gel coating were main factors influencing *bphA* transcription ($p<0.0001$) and both factors had interactions effect ($p<0.0001$). Environmental conditions significantly affected *bphA* transcription at different sol-gel coatings ($p<0.0003$). For sol-gel$_{GLY}$-coated biofilms, higher humic acid positively induced *bphA* transcript abundance than baseline (no humic acid)($p=0.034$)(Figure S22a), and baseline conditions also had higher *bphA* transcript abundance than DO changes and higher salinity ($p<0.0001$)(Figure S22a). For sol-gel$_{PEG}$-coated biofilms, higher humic acid also positively induced *bphA* trancript abundance more than baseline ($p=0.037$), but

higher salinity had lower *bphA* transcript abundance than baseline ($p<0.0001$)(Figure S22c). For uncoated biofilms, lower temperature was the key factor which significantly induced *bphA* transcripts ($p=0.037$) than baseline (Figure S22e).

Sol-gel$_{GLY}$-coated biofilms outcompeted sol-gel$_{PEG}$-coated biofilms under environmentally relevant conditions (baseline, temperature, and salinity). Sol-gel coatings also significantly affected *bphA* transcript at different environmental conditions ($p<0.007$), which demonstrated different conclusion on *bphA* transcript per gene ratio. It is notable that for all conditions, sol-gel$_{GLY}$-coated biofilms had significantly higher *bphA* transcripts than uncoated biofilms ($p<0.007$). For conditions like baseline, temperature, and salinity, sol-gel$_{GLY}$-coated biofilms displayed higher *bphA* transcripts than sol-gel$_{PEG}$-coated biofilms ($p<0.018$). Both findings highlight the importance of sol-gel$_{GLY}$ in inducing *bphA* transcription. Sol-gel$_{PEG}$ coating effects on *bphA* transcription were inconsistent depending on environmental conditions; sol-gel$_{PEG}$-coated biofilms had higher *bphA* transcripts/g than uncoated biofilms under baseline conditions and the presence of humic acid ($p<0.0014$), but had less *bphA* transcripts than uncoated biofilms under 10 °C ($p=0.0001$).

Comparing *bphA* transcription between day-45 and day-0, most environmental conditions demonstrated negative impacts on *bphA* transcription after 45 days, regardless of coating. For example, uncoated biofilms had significantly lower *bphA* transcription at day-45 compared to day-0 under all environmental conditions except temperature ($p=0.0007$)(Figure S22). Sol-gel$_{PEG}$-coated biofilms also demonstrated lower *bphA* transcription after treatment under all conditions except humic acid ($p<0.046$). Sol-gel$_{GLY}$-coated biofilms were also negatively affected by variable DO and salinity ($p<0.0001$), but humic acid induced higher *bphA* trancription after 45-days of treatment ($p=0.0001$)(Figure S22a). *BphA* trancript changes in sol-gel coated and uncoated biofilms over time were different from *bphA* expression levels under some environmental

conditions. For example, humic acid only had negative impacts on *bphA* expression levels in sol-gel$_{PEG}$-coated biofilms but no significant impacts on sol-gel$_{GLY}$-coated and uncoated biofilms. This indicates that more *bphA* was available for both sol-gel$_{GLY}$ and sol-gel$_{PEG}$ coated biofilms and less *bphA* available for uncoated biofilms at day-45 than day-0 at 2 mg/L humic acid for transcription, which was proved in Figure S22, thus normalizing *bphA* transcription by *bphA* abundance is important to demonstrate real gene expression level.

Sol-gel coated biofilms maintained higher bphA abundance than uncoated biofilms, and sol-gel$_{GLY}$ *better protected bphA abundance against environmental conditions than* sol-gel$_{PEG}$. Sol-gel coating and environmental conditions were also main factors significantly influencing *bphA* abundance and both factors had interactions effect ($p<0.0001$). Environmental conditions significantly affected *bphA* abundance at different sol-gel coatings ($p<0.023$). Sol-gel$_{PEG}$-coated biofilms under baseline conditions had significantly higher *bphA* abundance than lower temperature, DO changes, and higher salinity ($p<0.017$)(Figure S22d). Sol-gel$_{GLY}$ coating had much less impacts on biofilm abundance than sol-gel$_{PEG}$ coating, and only temperature had significant negative impacts on *bphA* abundance compared to DO and humic acid ($p<0.038$)(Figure S22b&d). As for uncoated biofilms, *bphA* abundance under variable DO was significantly lower than baseline ($p=0.0022$)(Figure S22f). This indicates that sol-gel$_{GLY}$ coated biofilms were less vulnerable to environmental changes than sol-gel$_{PEG}$ coated biofilms potentially due to biodegradability differences between any glycerol or PEG400 that may have leached from the sol-gel, but temperature had negative impacts on biofilm integrity regardless of the sol-gel coating recipe used.

Sol-gel coatings also significantly affected *bphA* abundance at different environmental conditions ($p<0.0001$). Under humic acid, DO changes, and baseline conditions, uncoated biofilms

had significantly lower *bphA* abundance than sol-gel coated biofilms (both sol-gel$_{GLY}$ and sol-gel$_{PEG}$)($p<0.03$). At 10°C, sol-gel$_{PEG}$ coated biofilms maintained higher *bphA* abundance than sol-gel$_{GLY}$ coated biofilms and uncoated biofilms ($p<0.0003$). At 20g/L salinity, sol-gel$_{GLY}$ coating performed better to preserve *bphA* abundance than sol-gel$_{PEG}$ coating and no coating ($p<0.002$) and even no coating could maintain significantly more *bphA* abundance than sol-gel$_{PEG}$ coating ($p=0.002$). As indicated in Figure S23, sol-gel$_{GLY}$ coating maintained gel integrity better under humic acid and salinity conditions, and sol-gel$_{PEG}$ coating also maintained gel integrity under humic acid condition, both of which support long-term biofilm abundance.

Interestingly, comparing *bphA* abundance at day-45 with day-0, both sol-gel$_{GLY}$ and sol-gel$_{PEG}$ coated biofilms had significantly higher *bphA* abundance after treatment under all environmental conditions ($p<0.011$), except sol-gel$_{PEG}$coating at 10°C ($p=0.052$). However, uncoated biofilms either had lower *bphA* abundance (e.g., DO, $p=0.003$) after treatment or no significant changes (e.g., the other four conditions)(Figure S22). This emphasizes the importance of sol-gel coating in protecting or even enriching biofilm abundance.

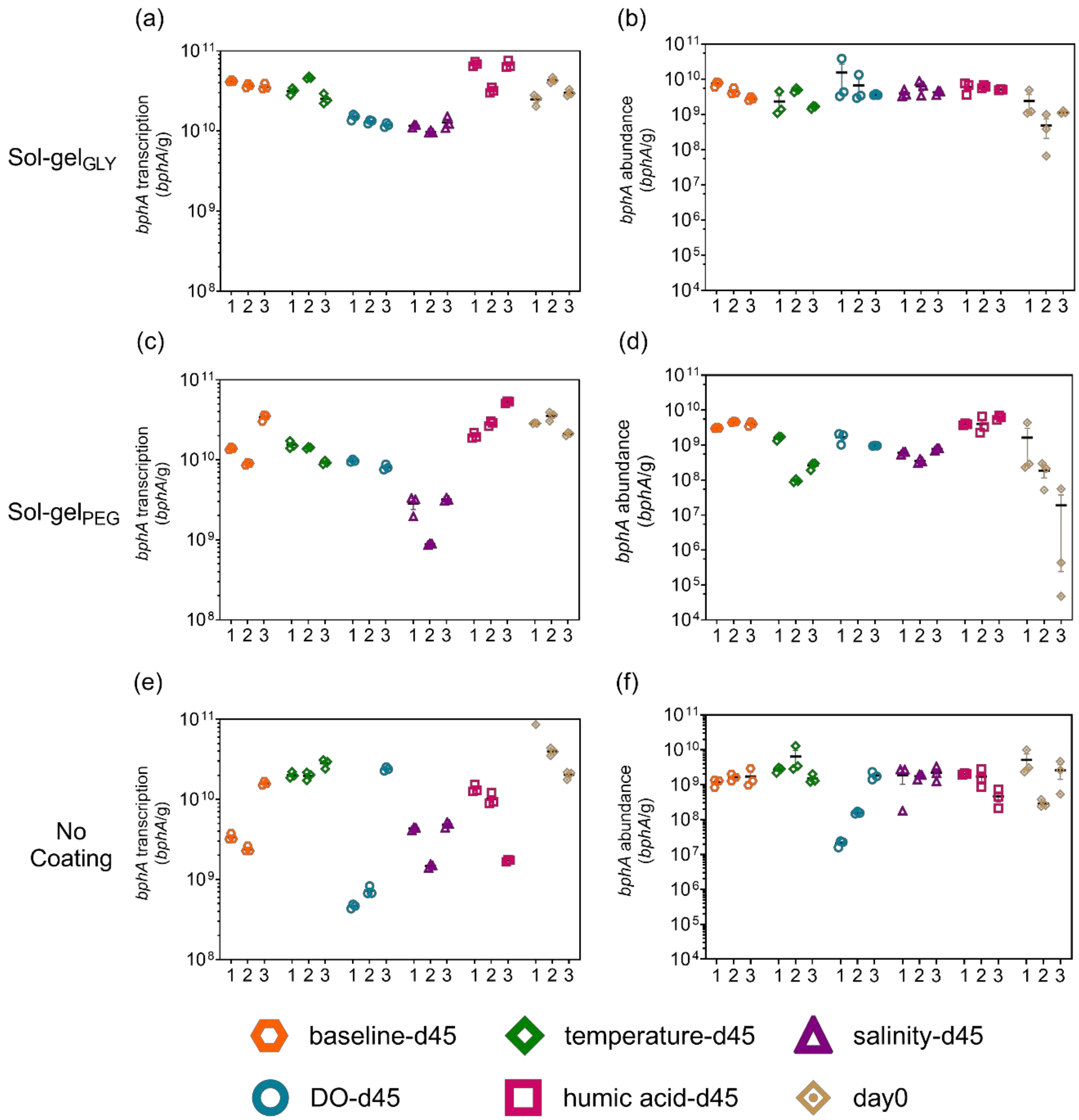

Figure S22. *BphA* transcript abundance and gene abundance under variable environmental conditions before and after 45 days of treatment. Each row represent different sol-gel coating conditions: sol-gel$_{GLY}$ coating, sol-gel$_{PEG}$ coating, and no coating. (a)(c)(e) represent *bphA* transcription of sol-gel coated and uncoated biofilms (unit: *bphA*/g) at day-0 and day-45 under different environmental conditions. (b)(d)(f) represent *bphA* abundance of sol-gel coated and uncoated biofilms (unit: *bphA*/g) at day-0 and day-45 under different environmental conditions. Environmental conditions include baseline conditions (fresh water, fully aerobic, room temperature, 20-21°C), temperature (10 °C), dissolved oxygen (variable DO changes), salinity (20 g/L), and humic acid (2 mg/L). Error bars represent standard errors of three biological replicates (n=3), and each biological replicate contains three technical replicates (n=3). Black lines represent mean of biological replicates.

Sol-gel coating protected biofilm abundance against environmental changes, especially sol-gel$_{GLY}$. Before treatment, biofilms were completely covered by sol-gel (sol-gel$_{GLY}$ or sol-gel$_{PEG}$) with rough gel morphology on LB400 cells, while no sol-gel coating had flat biochar surface with only biofilms on the top (Figure S23, day0).

After 45 days of treatment, the sol-gel$_{GLY}$ coating maintained a better gel integrity than the sol-gel$_{PEG}$ coating in general, whereas uncoated biofilms were less abundant on the biochar surface compared to sol-gel coated biofilmsunder environmental conditions (Figure S23, day 45), which is consistent with our *bphA* abundance findings described above. Specifically, sol-gel$_{GLY}$ still covered most of the biochar surface and maintained cell abundance after treatment under 10°C, 2 mg/L humic acid, and 20 g/L salinity, while baseline conditions and variable DOs had partial biochar surface left uncovered. The sol-gel$_{PEG}$ coating was more affected by environmental conditions. For all conditions other than humic acid, the sol-gel$_{PEG}$ coating was thinner than the sol-gel$_{GLY}$ coating. Cell abundance was affected by sol-gel coverage and thickness, where thicker gel had more cells underneath while thinner gel maintained less cells (e.g., DO and salinity groups). This is an important finding to demonstrate the applicability of sol-gel coating on protecting biofilms against adverse environmental conditions.

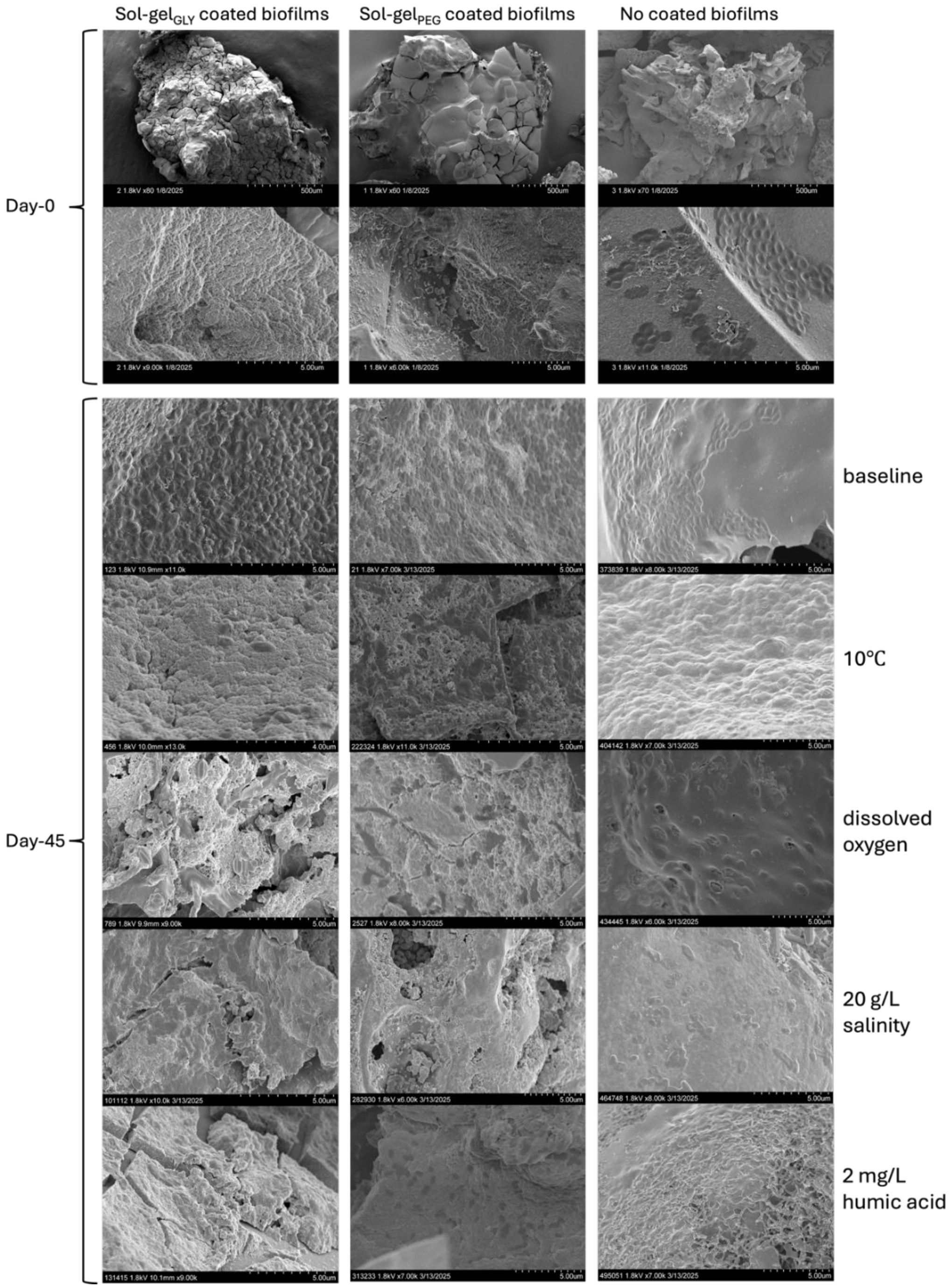

Figure S23. SEM imaging of sol-gel coated and uncoated biofilms at day-0 and day-45. Three columns represent different sol-gel coating conditions of biofilm-CKB, including sol-gel$_{GLY}$, sol-gel$_{PEG}$, and no coating. Top two rows are biofilms with different coatings at different magnifications before treatment (day-0). Bottom five rows are biofilms with different coatings after treatment (day-45) at different environmental conditions, including baseline conditions (fresh water, fully aerobic, room temperature, 20-21°C), temperature (10 °C), dissolved oxygen (variable DO changes), salinity (20 g/L), and humic acid (2 mg/L).

Sol-gel coating improved long-term biofilm abundance/viability. Before treatment at day-0, both sol-gel recipes had well covered (continuous green fluorescence) and thick gel coating on biochar with more live cell number than dead cells (live to dead cell ratio: >2:1), and no sol-gel coating also had high live to dead cell ratios on biochar surface (3.6:1) (Figure S24, Table S9).

After 45-days of treatment, environmental conditions affected cell abundance and viability at different sol-gel coatings. Comparing total cell number between day 0 and day 45, sol-gel_{GLY} protected cell abundance against baseline conditions and 20g/L salinity, and sol-gel_{PEG} protected cell abundance against humic acid (Figure S24, Table S10). For sol-gel_{GLY}-coated biofilms, 71% of remaining cells were viable under variable DO, albeit the lowest total cell number among all environmental conditions, but about half of the cells (41-52%) were dead under baseline conditions, 10°C, 20 g/L salinity, and 2 mg/L humic acid. Particularly, humic acid had much lower total cell number in sol-gel_{GLY} groups compared to other environmental conditions (18-63% of total cell number under baseline, temperature, and salinity conditions), which is controversial to *bphA* abundance results in Figure S22b. CLSM imaging has the limitation of sample heterogeneity because we can only visualize small areas with high resolution, but multiple areas (n=2) were evaluated to mitigate the limitation. For sol-gel_{PEG}, DO changes condition had over 70% of remaining cells were dead, while humic acid had ~2:1 live to dead cell ratios and >2.3 times more total cell number than other sol-gel_{PEG} groups and humic acid group of sol-gel_{GLY}. However, all tested environmental conditions interfered with cell abundance of uncoated biofilms (>25%) after 45 days except humic acid. Uncoated biofilms had over 74% of remaining cells were live under DO changes although the total cell number decreased 40% compared to day-0.

Comparing each environmental condition with baseline condition at day-45, sol-gel_{GLY} performed better than sol-gel_{PEG} to keep cell viable and abundant. Sol-gel_{PEG} had less total cell

number than sol-gel$_{GLY}$ (10-75% total cells of glycerol coating), except humic acid. Sol-gel$_{GLY}$ protected cell viability against all environmental conditions, which had >18% higher live to dead cell ratio than baseline. Sol-gel$_{PEG}$ protected cell viability under most conditions except DO changes (>70% were dead), but Sol-gel$_{PEG}$ had >15% more cell viability in low temperature and 2mg/L humic acid than baseline.

Table S10. Cell Viability for Sol-Gel Coated and Uncoated Biofilms at Day-0 for the environmental conditions experiment.

	live			dead			live/dead		
day 0	sample 1	sample 2	sample 3	sample 1	sample 2	sample 3	sample 1	sample 2	sample 3
Sol-gel$_{GLY}$ coat	187	1524	1205	150	452	121	1.25	3.37	9.96
Sol-gel$_{PEG}$ coat	534	724	520	171	413	450	3.12	1.75	1.16
Uncoated	206	336	1966	94	96	505	2.19	3.50	3.89

Table S11. Cell Viability for Sol-Gel Coated and Uncoated Biofilms under Different Environmental Conditions at Day-45.

		live		dead		live/dead	
	day 45	sample 1	sample 2	sample 1	sample 2	sample 1	sample 2
Sol-gel$_{GLY}$ coat	baseline	2237	3339	2224	3788	1.01	0.88
	10 degree C	422	1391	171	1305	2.47	1.07
	DO	612	746	133	430	4.60	1.73
	20 g/L salinity	2018	1632	1908	1428	1.06	1.14
	2 mg/L humic acid	732	486	582	281	1.26	1.73
Sol-gel$_{PEG}$ coat	baseline	170	692	225	188	0.76	3.68
	10 degree C	855	386	206	115	4.15	3.36
	DO	172	265	645	353	0.27	0.75
	20 g/L salinity	200	255	173	77	1.16	3.31
	2 mg/L humic acid	536	2786	284	1099	1.89	2.54
Uncoated	baseline	155	256	380	148	0.41	1.73
	10 degree C	397	275	431	73	0.92	3.77
	DO	545	409	142	190	3.84	2.15
	20 g/L salinity	451	383	648	130	0.70	2.95
	2 mg/L humic acid	291	1343	51	720	5.71	1.87

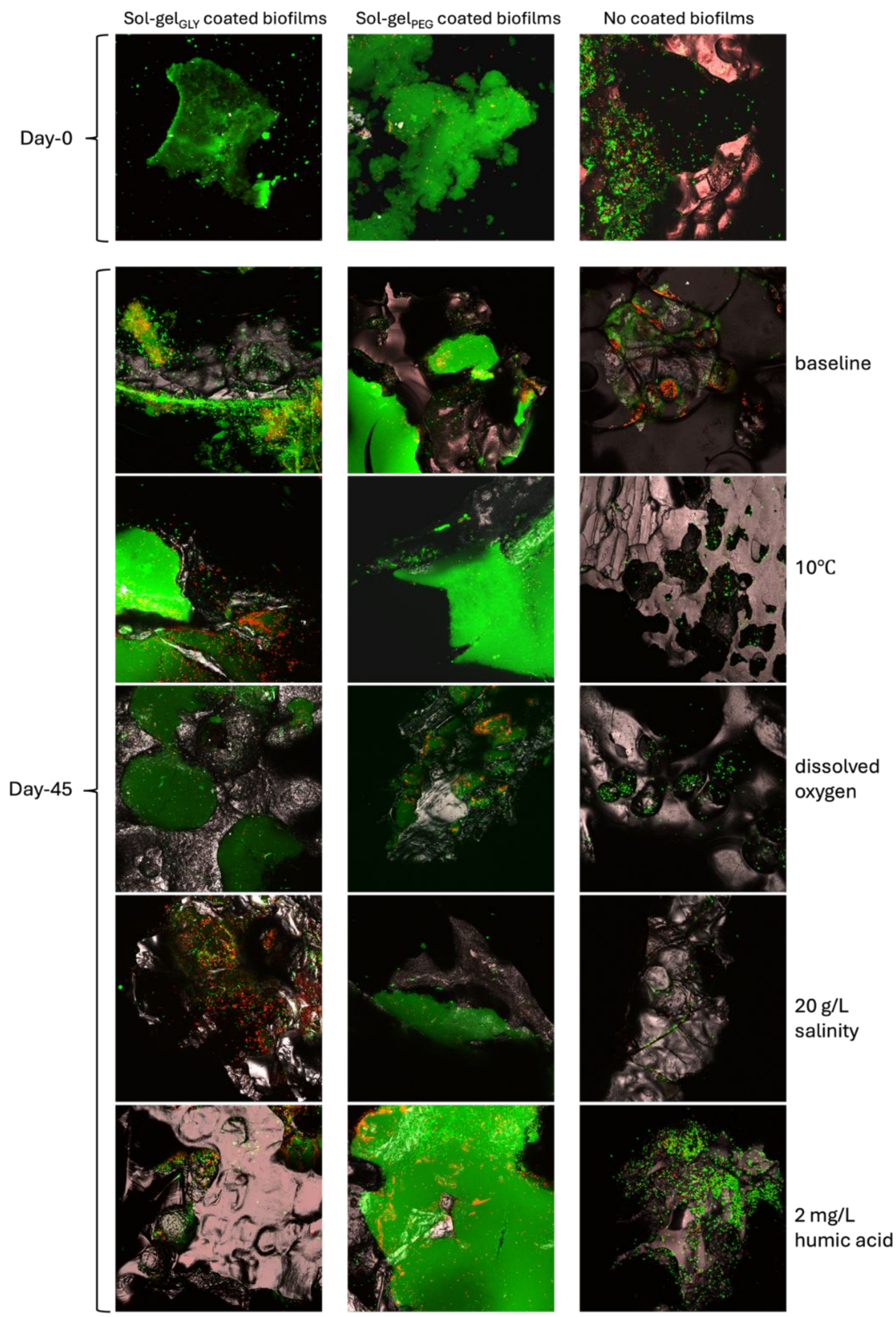

Figure S24. Confocal imaging of sol-gel coated and uncoated biofilms at day-0 and day-45. Three columns represent different sol-gel coating conditions of biofilm-CKB, including sol-gel$_{GLY}$, sol-gel$_{PEG}$, and no coating. Green dots represent live cells, and red dots represent dead cells. Green continuous fluorescence represents sol-gel. Row 1 is coated and uncoated biofilms before treatment (day-0), while row 2-6 are coated and uncoated biofilms after treatment (day-45) under different environmental conditions, including baseline conditions (fresh water, fully aerobic, room temperature, 20-21°C), temperature (10 °C), dissolved oxygen (variable DO), salinity (20 g/L), and humic acid (2 mg/L).

References

(1) Ghosh, U.; Kane Driscoll, S.; Burgess, R. M.; Jonker, M. T.; Reible, D.; Gobas, F.; Choi, Y.; Apitz, S. E.; Maruya, K. A.; Gala, W. R., Passive sampling methods for contaminated sediments: Practical guidance for selection, calibration, and implementation. *Integrated environmental assessment and management* **2014,** *10*, (2), 210-223.

(2) Adams, R. G.; Lohmann, R.; Fernandez, L. A.; MacFarlane, J. K.; Gschwend, P. M., Polyethylene devices: Passive samplers for measuring dissolved hydrophobic organic compounds in aquatic environments. *Environmental science & technology* **2007,** *41*, (4), 1317-1323.

(3) Jonker, M. T., Polyethylene–water and polydimethylsiloxane–water partition coefficients for polycyclic aromatic hydrocarbons and polychlorinated biphenyls: Influence of polymer source and proposed best available values. *Environmental Toxicology and Chemistry* **2022,** *41*, (6), 1370-1380.

(4) Jonker, M. T. O.; van der Heijden, S. A.; Kotte, M.; Smedes, F., Quantifying the Effects of Temperature and Salinity on Partitioning of Hydrophobic Organic Chemicals to Silicone Rubber Passive Samplers. *Environmental Science & Technology* **2015,** *49*, (11), 6791-6799.

(5) Gschwend, P. M.; MacFarlane, J. K.; Reible, D. D.; Lu, X.; Hawthorne, S. B.; Nakles, D. V.; Thompson, T., Comparison of polymeric samplers for accurately assessing PCBs in pore waters. *Environmental Toxicology and Chemistry* **2011,** *30*, (6), 1288-1296.

(6) Jonker, M. T. O.; Koelmans, A. A., Polyoxymethylene Solid Phase Extraction as a Partitioning Method for Hydrophobic Organic Chemicals in Sediment and Soot. *Environmental Science & Technology* **2001,** *35*, (18), 3742-3748.

(7) Lu, X.; Skwarski, A.; Drake, B.; Reible, D. D., Predicting bioavailability of PAHs and PCBs with porewater concentrations measured by solid‐phase microextraction fibers. *Environmental Toxicology and Chemistry* **2011,** *30*, (5), 1109-1116.

(8) Martinez, A.; O'Sullivan, C.; Reible, D.; Hornbuckle, K. C., Sediment pore water distribution coefficients of PCB congeners in enriched black carbon sediment. *Environmental Pollution* **2013,** *182*, 357-363.

(9) Mayer, P.; Vaes, W. H. J.; Wijnker, F.; Legierse, K. C. H. M.; Kraaij, R.; Tolls, J.; Hermens, J. L. M., Sensing Dissolved Sediment Porewater Concentrations of Persistent and Bioaccumulative Pollutants Using Disposable Solid-Phase Microextraction Fibers. *Environmental Science & Technology* **2000,** *34*, (24), 5177-5183.

(10) Mayer, P.; Vaes, W. H. J.; Hermens, J. L. M., Absorption of Hydrophobic Compounds into the Poly(dimethylsiloxane) Coating of Solid-Phase Microextraction Fibers: High Partition Coefficients and Fluorescence Microscopy Images. *Analytical Chemistry* **2000,** *72*, (3), 459-464.

(11) Yang, Z.-Y.; Zeng, E. Y.; Xia, H.; Wang, J.-Z.; Mai, B.-X.; Maruya, K. A., Application of a static solid-phase microextraction procedure combined with liquid–liquid extraction to determine poly(dimethyl)siloxane–water partition coefficients for selected polychlorinated biphenyls. *Journal of Chromatography A* **2006,** *1116*, (1), 240-247.

(12) Zeng, E. Y.; Tsukada, D.; Noblet, J. A.; Peng, J., Determination of polydimethylsiloxane–seawater distribution coefficients for polychlorinated biphenyls and chlorinated pesticides by solid-phase microextraction and gas chromatography–mass spectrometry. *Journal of Chromatography A* **2005,** *1066*, (1), 165-175.

(13) Heringa, M. B.; Hermens, J. L. M., Measurement of free concentrations using negligible depletion-solid phase microextraction (nd-SPME). *TrAC Trends in Analytical Chemistry* **2003,** *22*, (9), 575-587.
(14) Lotufo, G. R.; Michalsen, M. M.; Reible, D. D.; Gschwend, P. M.; Ghosh, U.; Kennedy, A. J.; Kerns, K. M.; Rakowska, M. I.; Odetayo, A.; MacFarlane, J. K.; Yan, S.; Bokare, M., Interlaboratory Study of Polyethylene and Polydimethylsiloxane Polymeric Samplers for Ex Situ Measurement of Freely Dissolved Hydrophobic Organic Compounds in Sediment Porewater. *Environmental Toxicology and Chemistry* **2022,** *41*, (8), 1885-1902.
(15) Heringa, M. B.; Pastor, D.; Algra, J.; Vaes, W. H. J.; Hermens, J. L. M., Negligible Depletion Solid-Phase Microextraction with Radiolabeled Analytes To Study Free Concentrations and Protein Binding: An Example with [3H]Estradiol. *Analytical Chemistry* **2002,** *74*, (23), 5993-5997.
(16) Conder, J. M.; La Point, T. W.; Lotufo, G. R.; Steevens, J. A., Nondestructive, Minimal-Disturbance, Direct-Burial Solid-Phase Microextraction Fiber Technique for Measuring TNT in Sediment. *Environmental Science & Technology* **2003,** *37*, (8), 1625-1632.
(17) Styrishave, B.; Mortensen, M.; Henning Krogh, P.; Andersen, O.; Jensen, J., Solid-Phase Microextraction (SPME) as a Tool to Predict the Bioavailability and Toxicity of Pyrene to the Springtail, Folsomia candida, under Various Soil Conditions. *Environmental Science & Technology* **2008,** *42*, (4), 1332-1336.
(18) You, J.; Landrum, P. F.; Lydy, M. J., Comparison of Chemical Approaches for Assessing Bioavailability of Sediment-Associated Contaminants. *Environmental Science & Technology* **2006,** *40*, (20), 6348-6353.
(19) Bannavti, M. K.; Jahnke, J. C.; Marek, R. F.; Just, C. L.; Hornbuckle, K. C., Room-to-Room Variability of Airborne Polychlorinated Biphenyls in Schools and the Application of Air Sampling for Targeted Source Evaluation. *Environmental Science & Technology* **2021,** *55*, (14), 9460-9468.
(20) Marek, R. F.; Thorne, P. S.; Herkert, N. J.; Awad, A. M.; Hornbuckle, K. C., Airborne PCBs and OH-PCBs Inside and Outside Urban and Rural U.S. Schools. *Environmental Science & Technology* **2017,** *51*, (14), 7853-7860.
(21) Martinez, A.; Erdman, N. R.; Rodenburg, Z. L.; Eastling, P. M.; Hornbuckle, K. C., Spatial distribution of chlordanes and PCB congeners in soil in Cedar Rapids, Iowa, USA. *Environmental Pollution* **2012,** *161*, 222-228.
(22) Martinez, A.; Norström, K.; Wang, K.; Hornbuckle, K. C., Polychlorinated biphenyls in the surficial sediment of Indiana Harbor and Ship Canal, Lake Michigan. *Environment International* **2010,** *36*, (8), 849-854.
(23) Martinez, A.; Hua, J. B. X.; Haque, E.; Hornbuckle, K. C.; Thorne, P. S., Occurrence and spatial distribution of individual polychlorinated biphenyl congeners in residential soils from East Chicago, southwest Lake Michigan. *Science of The Total Environment* **2022,** *850*, 157705.
(24) Pehkonen, S.; You, J.; Akkanen, J.; Kukkonen, J. V. K.; Lydy, M. J., Influence of black carbon and chemical planarity on bioavailability of sediment ‐ associated contaminants. *Environmental Toxicology and Chemistry* **2010,** *29*, (9), 1976-1983.
(25) Zimmerman, J. R.; Ghosh, U.; Millward, R. N.; Bridges, T. S.; Luthy, R. G., Addition of Carbon Sorbents to Reduce PCB and PAH Bioavailability in Marine Sediments: Physicochemical Tests. *Environmental Science & Technology* **2004,** *38*, (20), 5458-5464.

(26) Jahnke, A.; Mayer, P., Do complex matrices modify the sorptive properties of polydimethylsiloxane (PDMS) for non-polar organic chemicals? *Journal of Chromatography A* **2010,** *1217*, (29), 4765-4770.
(27) Urrestarazu Ramos, E.; Meijer, S. N.; Vaes, W. H. J.; Verhaar, H. J. M.; Hermens, J. L. M., Using Solid-Phase Microextraction To Determine Partition Coefficients to Humic Acids and Bioavailable Concentrations of Hydrophobic Chemicals. *Environmental Science & Technology* **1998,** *32*, (21), 3430-3435.
(28) Jaward, F. M.; Farrar, N. J.; Harner, T.; Sweetman, A. J.; Jones, K. C., Passive Air Sampling of PCBs, PBDEs, and Organochlorine Pesticides Across Europe. *Environmental Science & Technology* **2004,** *38*, (1), 34-41.
(29) Goryunova, K.; Gahramanli, Y.; Gurbanova, R., Adsorption properties of silica aerogel-based materials. *RSC advances* **2023,** *13*, (27), 18207-18216.
(30) Hoyos-Leyva, J. D.; Bello-Pérez, L. A.; Agama-Acevedo, E.; Alvarez-Ramirez, J., Thermodynamic analysis for assessing the physical stability of core materials microencapsulated in taro starch spherical aggregates. *Carbohydrate Polymers* **2018,** *197*, 431-441.
(31) Zhang, X.; Wang, H.; Li, Q.; Yin, Z.; Qi, H.; Yang, J.; Wang, X.; Xiao, W.; Zhang, L., Development of Organogels for Live Yarrowia lipolytica Encapsulation. *Journal of the American Chemical Society* **2022,** *144*, (23), 10251-10258.
(32) Altstein, M.; Aziz, O. B.; Skalka, N.; Bronshtein, A.; Chuang, J. C.; Van Emon, J. M., Development of an immunoassay and a sol–gel-based immunoaffinity cleanup method for coplanar polychlorinated biphenyls from soil and sediment samples. *Analytica Chimica Acta* **2010,** *675*, (2), 138-147.
(33) Cabrol, L.; Quéméneur, M.; Misson, B., Inhibitory effects of sodium azide on microbial growth in experimental resuspension of marine sediment. *Journal of Microbiological Methods* **2017,** *133*, 62-65.
(34) Dong, Q.; LeFevre, G. H.; Mattes, T. E., Black Carbon Impacts on Paraburkholderia xenovorans Strain LB400 Cell Enrichment and Activity: Implications toward Lower-Chlorinated Polychlorinated Biphenyls Biodegradation Potential. *Environmental Science & Technology* **2024,** *58*, (8), 3895-3907.
(35) Richards, P. M.; Liang, Y.; Johnson, R. L.; Mattes, T. E., Cryogenic soil coring reveals coexistence of aerobic and anaerobic vinyl chloride degrading bacteria in a chlorinated ethene contaminated aquifer. *Water Research* **2019,** *157*, 281-291.
(36) Johnson, D. R.; Lee, P. K.; Holmes, V. F.; Alvarez-Cohen, L., An internal reference technique for accurately quantifying specific mRNAs by real-time PCR with application to the tceA reductive dehalogenase gene. *Appl Environ Microbiol* **2005,** *71*, (7), 3866-71.
(37) Petrić, I.; Hršak, D.; Fingler, S.; Udiković-Kolić, N.; Bru, D.; Martin-Laurent, F., Insight in the PCB-degrading functional community in long-term contaminated soil under bioremediation. *Journal of soils and sediments* **2011,** *11*, 290-300.
(38) Liang, Y.; Meggo, R.; Hu, D.; Schnoor, J. L.; Mattes, T. E., Enhanced polychlorinated biphenyl removal in a switchgrass rhizosphere by bioaugmentation with *Burkholderia xenovorans* LB400. *Ecological engineering* **2014,** *71*, 215-222.
(39) Liang, Y.; Liu, X.; Singletary, M. A.; Wang, K.; Mattes, T. E., Relationships between the abundance and expression of functional genes from vinyl chloride (VC)-degrading bacteria and geochemical parameters at VC-contaminated sites. *Environmental Science & Technology* **2017,** *51*, (21), 12164-12174.

(40) Parnell, J. J.; Denef, V. J.; Park, J.; Tsoi, T.; Tiedje, J. M., Environmentally relevant parameters affecting PCB degradation: carbon source-and growth phase-mitigated effects of the expression of the biphenyl pathway and associated genes in *Burkholderia xenovorans* LB400. *Biodegradation* **2010,** *21*, 147-156.

(41) Seeger, M.; Timmis, K. N.; Hofer, B., Degradation of chlorobiphenyls catalyzed by the bph-encoded biphenyl-2, 3-dioxygenase and biphenyl-2, 3-dihydrodiol-2, 3-dehydrogenase of *Pseudomonas* sp. LB400. *FEMS microbiology letters* **1995,** *133*, (3), 259-264.

(42) Potrawfke, T.; Löhnert, T. H.; Timmis, K. N.; Wittich, R. M., Mineralization of low-chlorinated biphenyls by Burkholderia sp. strain LB400 and by a two-membered consortium upon directed interspecies transfer of chlorocatechol pathway genes. *Applied Microbiology and Biotechnology* **1998,** *50*, (4), 440-446.

(43) Sander, R., Compilation of Henry's law constants (version 5.0.0) for water as solvent. *Atmos. Chem. Phys.* **2023,** *23*, (19), 10901-12440.

(44) Harkness, M.; McDermott, J.; Abramowicz, D.; Salvo, J.; Flanagan, W.; Stephens, M.; Mondello, F.; May, R.; Lobos, J.; Carroll, K., In situ stimulation of aerobic PCB biodegradation in Hudson River sediments. *Science* **1993,** *259*, (5094), 503-507.
